# Design, Synthesis, and Antipoliferative Activities of Novel Substituted Imidazole-Thione Linked Benzotriazole Derivatives

**DOI:** 10.3390/molecules26195983

**Published:** 2021-10-02

**Authors:** Ahdab N. Khayyat, Khaled O. Mohamed, Azizah M. Malebari, Afaf El-Malah

**Affiliations:** 1 Department of Pharmaceutical Chemistry, College of Pharmacy, King Abdulaziz University, Jeddah 21589, Saudi Arabia; amelibary@kau.edu.sa (A.M.M.); aalmallah@kau.edu.sa (A.E.-M.); 2 Pharmaceutical Organic Chemistry Department, Faculty of Pharmacy, Cairo University, Cairo 11562, Egypt; khaled.mohamed@pharma.cu.edu.eg

**Keywords:** benzotriazole, anticancer, imidazol-2-thione, antiproliferative activity, breast cancer, colchicine, tubulin

## Abstract

A new series of benzotriazole moiety bearing substituted imidazol-2-thiones at N1 has been designed, synthesized and evaluated for in vitro anticancer activity against the different cancer cell lines MCF-7(breast cancer), HL-60 (Human promyelocytic leukemia), and HCT-116 (colon cancer). Most of the benzotriazole analogues exhibited promising antiproliferative activity against tested cancer cell lines. Among all the synthesized compounds, **BI9** showed potent activity against the cancer cell lines such as MCF-7, HL-60 and HCT-116 with IC_50_ 3.57, 0.40 and 2.63 µM, respectively. Compound **BI9** was taken up for elaborate biological studies and the HL-60 cells in the cell cycle were arrested in G_2_/M phase. Compound **BI9** showed remarkable inhibition of tubulin polymerization with the colchicine binding site of tubulin. In addition, compound **BI9** promoted apoptosis by regulating the expression of pro-apoptotic protein BAX and anti-apoptotic proteins Bcl-2. These results provide guidance for further rational development of potent tubulin polymerization inhibitors for the treatment of cancer.

## 1. Introduction

Microtubules are chief constituents of the cytoskeleton with a vital role in biological functions of all eukaryotic cells. They are essential for cell proliferation, shape maintenance, intracellular transportation, cell motility, cell division and mitosis. Chemically, they combined the two types of protein subunits, α- and β-tubulin heterodimers [1,2]. Targeting microtubules appeared as an effective strategy for anticancer agents, as those agents interfere with microtubule dynamic equilibrium of the reversible assembly- disassembly [3]. This interference is categorized to twin core groups: microtubule-stabilizing agents and microtubule-destabilizing agents that fasten to the tubulin polymer to secure microtubules (taxane) or to the tubulin dimers and destabilize microtubules (vinca alkaloid or colchicine), respectively [4].

Recently, a great effort has been given to picking out innovative microtubule-targeting agents as chemotherapeutic agents and specifically aiming them at the colchicine binding site [5,6,7,8]. Inhibitors binding to the colchicine binding site have numerous benefits to fulfill the criteria required for an ideal chemotherapeutic agent, including uncomplicated structures, enhanced hydrophilicity, scaled down toxicity, powerful anti-vascular activity, and considerable multidrug resistance (MDR) effects [9,10].

**Figure 1:**
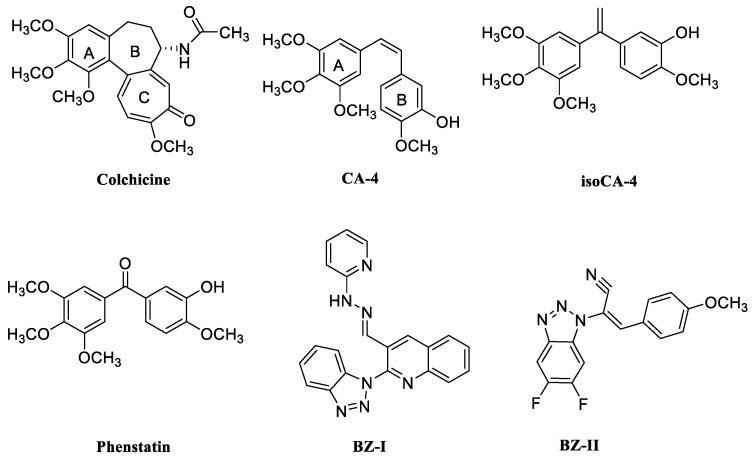
Chemical structure of Colchicine, CA-4 and its analogues and tubulin targeting agents bearing benzotriazole moiety (BZI & BZII).

Combretastatin A-4 (CA-4, 1, Figure 1) is a descriptive colchicine binding site in the tubulin which acts by inhibition of the microtubule polymerization. This natural phenol originated in the bark of Combretum caffrum, which is known as the African bush willow. [11]. CA-4 is an excellent candidate that has exhibited potent anti-vascular and anticancer activity along with its structural features, which stimulate its eligibility to become a lead molecule for designing new analogs [12]. However, the stilbenoid backbone of CA4 is easily isomerized during stock piling, administration and the breaking down to an E isomer which is thermodynamically way out stable but less potent, which reflects the critical chemical instability feature. Afterwards, an innovative chain of combretastatin spin-offs have been synthesized to bypass this stability drawback, just like isoCA-4 [7,13] and Phenstatin (Figure 1). Structural alterations of the combretastatin stilbene have been explored by joining the 1,2-diarylalkene link to a carbocyclic or heterocyclic ring system to obtain conformationally restricted analogues [14,15]. These hetero-combretastatin derivatives have been synthesized and inspired from several five-membered aromatic heterocyclic rings including tetrazole, pyrazole, imidazole, triazole, isoxazole, thiazole, oxadiazole-2-thione, thiazolidinone, and thiophene rings [16,17,18,19,20,21,22,23,24,25,26].

Benzotriazole-based compounds are unique nitrogen-containing heterocycles that have attracted significant attention from medicinal chemists as a promising class of bioactive heterocyclic products that exhibit numerous biological properties, such as anticancer [27,28,29,30,31] (BZI, Figure 1), antibacterial [32,33], antiviral [34,35], and anti-inflammatory activities [36,37]. Furthermore, substitution of the 3,4,5-trimethoxyphenyl ring of isoCA-4 by a quinazoline nucleus [38] or quinoline [39,40,41] led to tubulin inhibitor with potent antiproliferative deeds versus a variety of cancerous cell lines. Recently, BZII with a benzotriazole moiety for the replacement for trimethoxyphenyl moiety of colchicine was reported (Figure 1), and the docking studies of BZII showed that benzotriazole formed polar and hydrophobic interaction with the critical residue amino acids of α- and β-tubulin subunits in the colchicine-binding pocket. These data demonstrated that benzotriazole moiety might be a surrogate of the traditional 3,4,5-trimethoylphenyl moiety when binding to the colchicine site [9,30].

Based on these inspiring results, we have dedicated ourselves to designing and introducing novel anticancer agents pursuing a tubulin-microtubule system. We proposed a chain of 3-(benzotriazole)-2(3H)-imidazole-2-thiones as novel heterocyclic analogs of CA-4, in which the olefinic core structure of CA-4 is substituted by 2(3H)-imidzaole-2-thione. A new approach towards the development of a new series of novel imidazol-thione derivatives was proposed by replacing the 3,4,5-trimethoxyphenyl of CA-4 with the benzotriazole ring as ring A, and the hosted ring B with different substituents. In this paper we report on their synthesis and potent antitumor activities versus human cancer cell lines as well as cytotoxicity toward a representative normal human cell line HUVEC. In addition, the fundamental cytotoxic mechanisms of the typical compound **BI9** were also interpreted.

## 2. Results and Discussion

### 2.1. Design and Chemistry

**Scheme 1:**
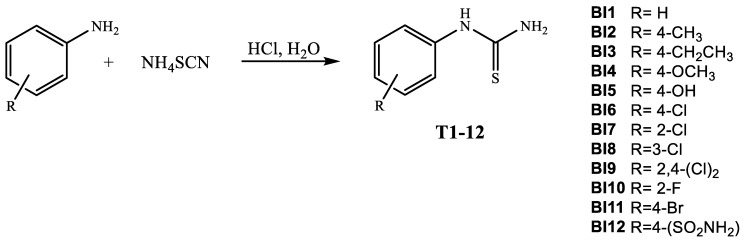
Preparation of some substituted thiourea **T1-12**.

**Scheme 2:**
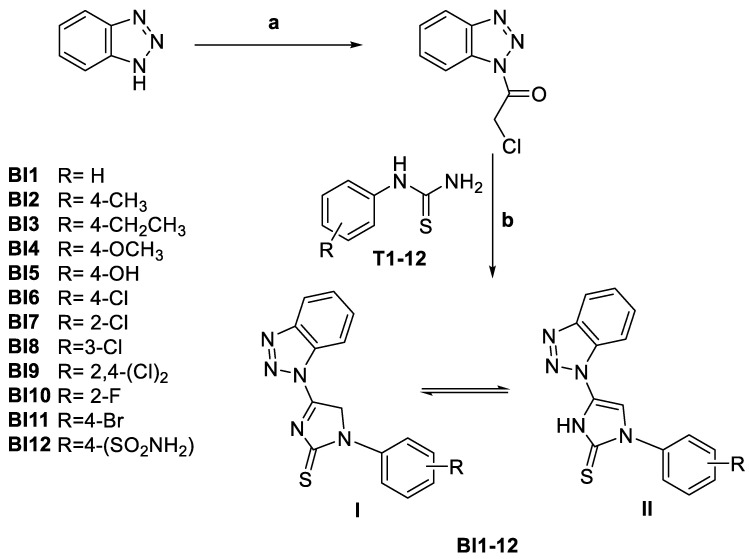
Synthesis of benzotriazoloimidazol-thione derivatives **BI1-12**. Reagents: (a) CICOCH_2_CI, dry acetone, NaOAc, 3 h; (b) Abs.EtOH,NaOAc, reflux, 6 h.

As illustrated in Scheme 1, some substituted thiourea derivatives **T1-12** were synthesized by refluxing some selected substituted anilines with ammonium thiocyanate in acidic aqueous solution. Novel benzotriazole bearing 3-substitutedphenylimidazol-2-thiones **BI1-12** were prepared in two steps as stated in Scheme 2. Initially, preparation of chloroethanone of benzotriazole, as intermediate, was prepared through the reaction between benzotriazole and chloroacetyl chloride. Then, the target structure **BI1-12** were synthesized via refluxing chloroethanone of benzotriazole with substituted thiourea derivatives **T1-12** in ethanol containing catalytic amount of anhydrous sodium acetate.

The structures of compounds **BI1-12** were confirmed using different spectroscopic methods and by elemental analysis. The IR spectra characteristically revealed the existence of peaks for NH groups which are tautomeric with CH_2_ group (C5) of imidazole ring moiety at range 3270–3340 cm^−1^, in addition to the presence of peaks of C=N at 1664–1662 cm^−1^. Regarding ^1^H NMR spectra, all synthesized benzotriazole imidazole-thione derivatives **BI1-12** present in two tautomeric forms I and II as stated in Scheme 2. They all exhibited the significant singlet signals corresponding to CH_2_ group (C5, imidazole) at δ 3.99–4.03 ppm which is tautomeric with NH group. Moreover, NH singlet signal observed at δ 11.04–11.75 ppm D_2_O exchangeable in compounds **BI1-6, BI8** and **BI11-12**. On the other hand, NH group in compounds **BI7, BI9** and **BI10** obviously showed singlet signals at δ 4.89–6.11 ppm with D_2_O exchangeable behavior in their spectra. Through ^1^HNMR, all of the benzotriazole analogues show a mixture of tautomer I and II in imidazole ring displaying that tautomer I, where C-5 in the imidazole ring is in the form of (-CH2-), represents the major one with ratio two times of tautomer II, where imidazole ring nitrogen bears hydrogen atom (NH) and C-5 is in form of (-CH=) [42]. This finding is consistent with the stability sequence of tautomer I, which is stabilized through conjugation (Supplementary Figure S1). Also, ^13^C NMR spectra of the prepared compounds **BI1-12** confirmed the formation of imidazole core as well as showed the presence of thione carbon at δ 188–188.55 ppm [43,44,45].

### 2.2. Biological Results and Discussion

#### 2.2.1. In Vitro Cell Growth Inhibitory Activity

Numerous Tumor-derived cell lines have been utilized to assess the influence of the synthesised compounds on cell viability, for example on MCF-7 breast adenocarcinoma, HL-60 leukemia, and HCT-116 colorectal carcinoma, as well as HUVEC human normal cell line was assayed by MTT assay. The outcomes are reviewed in Table 1. The imidazol-2-thiones compounds which are evaluated in this study were considered to enclose the benzotriazole substituent that mimics ring A in CA-4. The majority of compounds which were designed contain different substituents at position C-4 of the phenyl ring of the imidazol-2-thiones. Overall, those compounds reported a modest to potent growth inhibitory activity versus all triplet tumor cell lines used in this study.

The substituted phenyl ring of the imidazol-2-thiones compounds was reported to considerably affect the biological activity based on the category and nature of the substituent. The unsubstituted phenyl ring compound **BI1** demonstrated low micromolar range in MCF-7 and HL-60 while poor activity against HCT-116. Adding methyl substituent on the phenyl ring as in compound **BI2** lessened the activity, with IC_50_ values exceeding 10 µM entirely in the three cell lines. Substitution of the substituent in **BI2** by a greater electron-releasing ethyl group in **BI3** or methoxy group in **BI4**, resulted in an improvement in the antiproliferative action; **BI3** and **BI4** were 3.8-fold and 2.5-fold respectively more active than their corresponding methyl-containing derivative **BI2**. At the same time, trading the methyl group by polar group hydroxy to the phenyl ring of the imidazol-2-thione in **BI5** caused a weighty enhancement in the antiproliferative activity in submicromolar range versus MCF-7, HL-60 and HCT-116 with IC_50_ values of 4.4, 1.1 and 2.1 µM respectively.

Insertion of different electron withdrawing group as chloro group as in compounds **BI5**–**BI8** affected the cell viability with low micromolar range (IC_50_ values range: 1.51–15.5 µM) compared to their other halogen analogues containing fluoro (**BI10**) (IC_50_ values range: 7.02–21.5 µM) or bromo (**BI11**) derivatives (IC_50_ values range: 11.5–38.2 µM). Introduction of a chloro substituent on different location on the phenyl ring of imidazol-2-thione compounds resulted in good antiproliferative activity versus the cancer cell lines with negligible variances in IC_50_ values. For example, the antiproliferative activity of 4-chloro-substituted **BI6**, 2-chloro-substituted **BI7** and 3-chloro-substituted **BI8** exhibited comparable activity in low micromolar range versus HL-60 and HCT-116 cells with IC_50_ values between 1.51–7.38 µM. However, MCF-7 **BI7** (2-chloro-substituted) displayed less antiproliferative activity with an IC_50_ value of 15.5 µM compared to 7.29 and 2.66 µM for **6** (4-chloro-substituted) and **BI8** (3-chloro-substituted) respectively. It is of interest that combining compounds **BI6** and **BI7** to yield compound **BI9** (2,4-chloro-substituted) displayed impressive potency in antiproliferative activity; IC_50_ values for **BI9** fluctuated between 0.4 and 3.3 µM, which was more potent than its corresponding analogues **BI6** (IC_50_: 1.51–7.29 µM) and **BI7** (IC_50_: 3.42–15.5 µM). This result reflects the importance of the 2,4-dichloro substituent of the phenyl at imidazol-2-thione ring for optimum activity in cancer cells. Hosting bigger substituents at the phenyl ring as in **BI12** (4-suphonamide) led to improved activity in comparison to their equivalent analog **BI2** (4-methyl). Those marks showed that the substituted benzotriazole ring could be a decent alternate for the 3,4,5-trimethoxyphenyl ring of CA-4.

With the exception of compounds **BI2**, **BI5,** and **BI10,** all of the benzotriazole analogues (**BI1**, **BI3**, **BI4**, **BI6**–**BI9**, **BI11** and **BI12**) had little effect on the normal human umbilical vein endothelial cell, HUVEC (IC_50_: 17.3–135 µM). Most interestingly, the 2,4-chloro-substituted compound **BI9** exhibited potent anticancer activity toward the three cancer cell lines but low toxicity against HUVEC cells with an IC_50_ of 118 µM and 13.6 µM for the reference CA-4. The results demonstrated that these designed compounds might possess excellent selectivity over normal human cells, indicating a high safety index. Owing to the excellent antiproliferative activity of compound **BI9** in HL-60 (0.4 µM), it was considered in further studies as below.

#### 2.2.2. In Vitro Inhibition of Tubulin Polymerization and Colchicine Binding

Colchicine and CA-4 drag to the colchicine binding site by attachment to tubulin which resulting in hang-up of microtubule polymerization [22,46]. To explore if the antiproliferative activities of the topmost powerful compounds of the of imidazol-2-thione chain derived from a collaboration with tubulin, compounds **BI3**, **BI5–BI9** and **BI12** along with the benchmark compound CA-4 were assessed for their anti-tubulin polymerization activities besides the consequences of the binding of [^3^H] colchicine to tubulin, the results presented in Table 2.

Most compounds strongly inhibited tubulin assembly compared toward of CA-4 with average IC_50_ values 0.49–0.92 µM, except for the compound **BI8,** which was the least with an IC_50_ value of 3.05 µM. Compounds **BI5** and **BI9** were found to be the most active with IC_50_ values of 0.49 and 0.52 µM respectively, which are comparable to that of CA-4 (IC_50_ = 0.57 µM). Their results are in harmonization alongside the potent cell growth inhibitory action of **BI3** and **BI9** in cancer cells.

The same series of compounds were also evaluated at twin unalike concentrations (1 and 5 µM) on behalf of its capability to compete with colchicine for binding to tubulin by means of [^3^H] colchicine binding assay. All the compounds effectively hindered colchicine binding to tubulin by average 59–65% at 1 µM and 77–86% at 5 µM. Interestingly, compound **BI9** powerfully hindered colchicine binding to tubulin by 49% at 1 µM and 66% at 5 µM plus to a 1.2 fold greater potency compared to CA-4, with 64% and 85% inhibition, respectively. Consequently, that implies the involvement of compound **BI9** in tubulin polymerization inhibition over the colchicine-binding site. These outcomes are matched with formerly stated one on behalf of a sequence of linked benzotriazole correspondents, which powerfully evacuated colchicine from its binding site on the tubulin [30,31].

#### 2.2.3. HL-60 Cell Cycle Arrest

**Figure 2:**
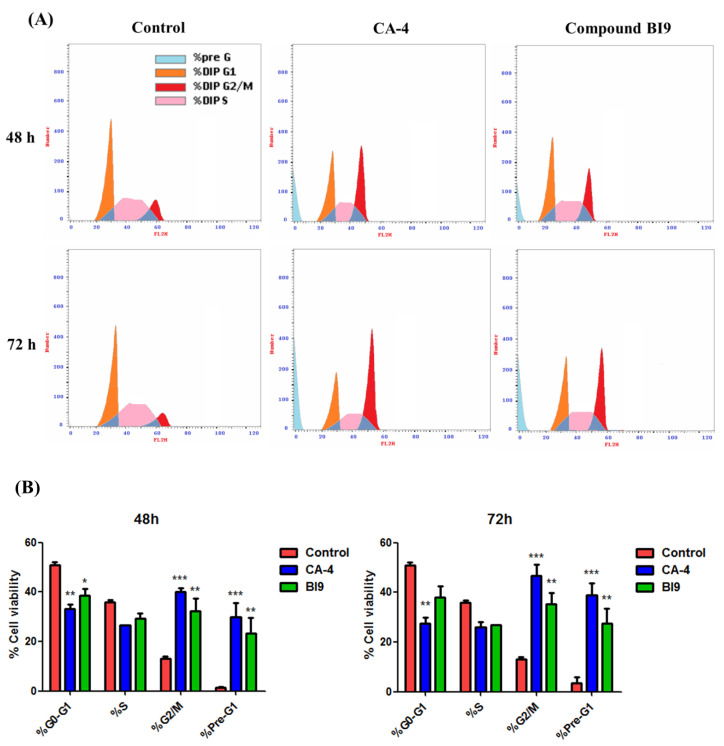
(**A**) Cell cycle analysis of HL-60 cells treated with compound **BI9** and CA-4 for 48 and 72 h. Cells were fixed and labeled with PI and analysed by flow cytometry as described in the experimental section. (**B**) The number of cells with 4N (G2/M), 2N(G0G1), and <2N (sub-G1) DNA content was determined with CellQuest software. Data are represented as mean of two independent experiments ± SEM. The asterisk indicate statistically significant differences.

It is well known that CA-4 as microtubule-targeting agent modify the tubulin-microtubule equilibrium and thus challenge the development of the mitotic spindles throughout the M phase, causing cell cycle arrest at G_2_/M phase and eventually prompt apoptosis [12,47,48]. The effect of the functioning compound benzotriazole analogue **BI9** at concentration 1 µM on the leukemia HL-60 cells was evaluated by flow cytometry for 48 and 72 h. As shown in Figure 2A, Compounds **BI9** brought clear G_2_/M arrest at 48 and 72 h with the proportions of HL-60 cells in G_2_/M phase of 32.3% and 35.1%, respectively. This discovery is comparable with CA-4 (50 nM) which produced a substantial escalation in the proportion of cells in G_2_/M arrest at 48 and 72 h with 40.1 and 46.6% of HL-60 cells respectively, connected to a lessening of cells in the cell cycle G0 phase (Figure 2B).

Furthermore, **BI9** initiated an important upturn in apoptosis as the population in the sub-G1 phase was amplified by 23.1 and 27.5% at 48 and 72 h, respectively, compared to 1.5 and 3.5% for untreated cells (Figure 2B). From the aforementioned results we can conclude that **BI9** demonstrated a significant upturn in the cell population of the HL-60 cancer cells in the G_2_/M phase, and induced cellular apoptosis in pre-G1 phase. These outcomes are in harmony with the formerly detected results for antimitotic spin-offs in the sequences of linked analogues which considerably encourage G_2_/M cycle arrest and apoptosis in MCF-7 cells [30,31].

#### 2.2.4. HL-60 Cell Apoptosis along with Alteration of Apoptosis Checkpoints Proteins

**Figure 3:**
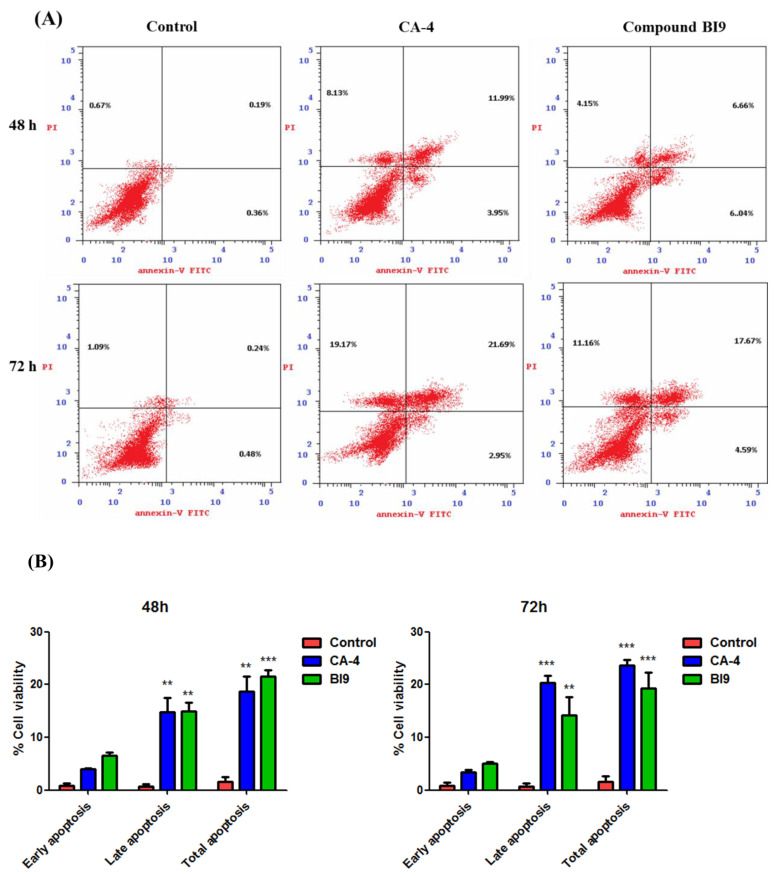
(**A**) Flow cytometric analysis of apoptotic cells after treatment of HL-60 cells with compounds **BI9** and CA-4 after incubation for 48 or 72 h. The cells were harvested and labeled with annexin-V-FITC and PI and analyzed by flow cytometry. (**B**) Quantitative analysis of apoptosis. Dual staining for annexin-V and with PI permits discrimination between live cells (annexin-V^−^/PI^−^), early apoptotic cells (annexin- V^+^/PI^−^), late apoptotic cells (annexin-V^+^/PI^+^) and necrotic cells (annexin-V^−^/PI^+^). Data are represented as mean ± SEM of two independent experiments. The asterisk indicate statistically significant differences.

Mitotic arrest of tumor cells by tubulin-directed agents is commonly linked with cellular apoptosis [49,50]. To see whether the compound **BI9** would stimulate cell apoptosis, HL-60 cells were dosed with **BI9** (1 µM) at time points 48 and 72 h, stained with Annexin-V/PI, and examined by flow cytometry. This double staining for annexin V and PI can offer insight among live cells (annexin V^−^/PI^−^), early apoptotic cells (annexin V^+^/PI^−^), late apoptotic cells (annexin V^+^/PI^−^), and necrotic cells (annexin V^−^/PI^+^). Compound **BI9** triggered a noteworthy buildup of annexin-V positive cells, persuading both early and late apoptosis in a time-dependent approach as related to the untreated control cells. As exhibited in Figure 3A, after HL-60 cells were exposed to CA-4 and **BI9** for 48 h time point, the total fractions of early apoptotic cells (annexin V^+^/PI^−^) and late apoptotic cells (annexin V^+^/PI^−^) considerably amplified from 1.6% in control cells to 18.7% and 21.5%, respectively. The average fraction of Annexin V-staining positive cells (total apoptotic cells) for CA-4 and **BI9** were 23.7% and 19.2% after 72 h, respectively, when compared to the control cells (1.6%) (Figure 3B). As stated from the cell cycle arrest and apoptosis outcomes mentioned earlier (Figure 2A,B), these outcomes confirmed that CA-4 analouge **BI9** could induce efficient apoptosis in HL-60 cancer cells.

**Figure 4:**
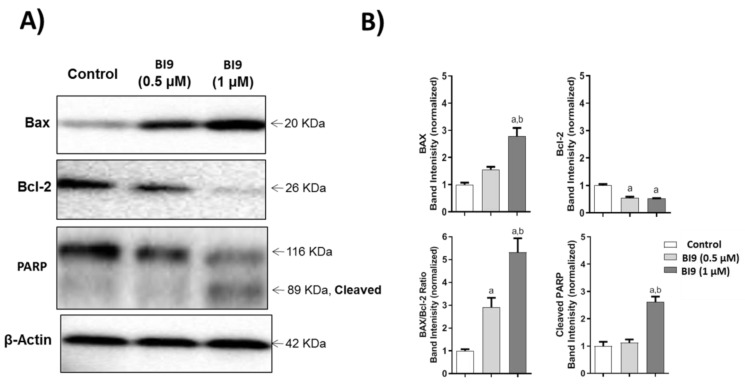
The immunoblotting of BAX, Bcl-2, and cleaved PARP (All were normalized to β-actin). (**A**) Representative western blots images show the effect of compound **BI9** at 0.5 µM and 1 µM concentrations on the expression levels of BAX, Bcl-2, and cleaved PARP proteins in HL-60 leukemia cells. (**B**) Quantification of the tested proteins in HL-60 leukemia cell lysates, both normalized the β-actin, as a protein internal control. The control group was set to ‘1’, and all data from three separate experiments are shown as mean ± SEM. ^a^ and ^b^ indicate statistically significant differences from the control and **BI9** (0.5 µM) group, respectively at *p* < 0.05, in one-way ANOVA with Tukey’s multiple comparisons test.

For additional examination of the effect of **BI9** on the apoptosis-related proteins expression in HL-60 cells, the consequences of compound **BI9** dosing on the expression of the pro-apoptotic protein Bax and anti-apoptotic protein Bcl-2 in HL60 cells was assessed. As shown in Figure 4, Western blotting of HL-60 cell extracts dosed by compound **BI9** at 0.5 and 1 µM for 84 h, the expression level of pro-apoptotic Bax protein was significantly up-regulated, and correspondingly the pro-survival protein of Bcl-2 was down-regulated compared to the untreated group. Furthermore, additional indicator of apoptosis was confirmed by poly ADP-reibose polymerase (PARP) cleavage of compound **BI9** after a 48 h treatment, confirming its proapototic activity. Taken together, the pattern in cell cycle analysis and apoptosis reveal that benzotriazole **BI9** was the most effective at regulating apoptosis-related protein expression in HL-60 cancer cells.

## 3. Materials and Methods

### 3.1. Chemistry

All reagents and solvents were used without further purification. All the recorded melting points were taken in an open glass capillary on a Griffin apparatus and the values given were uncorrected. Microanalyses for C, H, N, and S were carried out at the Regional Center for Mycology and Biotechnology, Faculty of Pharmacy, Al-Azhar University. C, H, N, S analysis values were accepted within a range of ±0.4% of theoretical calculated percentages. Also, Mass spectra were recorded on Jeol-Gas chromatography-mass spectrometry, JMS-Q1500GC (Tokyo, Japan) controlled with Escrime software. A direct insertion probe was used for sample infusion, source (EI^+^): source temperature was 210 °C. Electron energy was 70 eV, and trap-emission was 100 V. Data was acquired by applying a total MS scan from 40 to 1000 *m/z* (500 scan/s). IR spectra were determined using potassium bromide discs and values were represented in cm^−1^. IR spectra were recorded on Shimadzu IR 435 spectrophotometer (Shimadzu Corp., Kyoto, Japan) Faculty of Pharmacy, Cairo University. 1H NMR spectra were carried out on Bruker 400 MHz (Bruker Corp., Billerica, MA, USA) spectrophotometer, Faculty of Pharmacy, Cairo University. Chemical shifts were recorded in ppm on δ scale, coupling constants (J) were given in Hz and peak multiplicities are designed as follows: s, singlet; d, doublet; dd, doublet of doublet; t, triplet; q, quartet; qui, quintet; m, multiplet. 13C NMR spectra were carried out on Bruker 100 MHz spectrophotometer, Faculty of Pharmacy, Cairo University. The reaction progress was monitored by TLC using aluminum sheets precoated with UV fluorescent silica gel (MERCK 60F 254), spots were visualized using UV Lamp. The solvent system used was methanol, ethyl acetate, and toluene with a ratio of 1:2:3. The synthesis of some substituted thiourea **T1-12** as shown in Scheme 1 was achieved through refluxing a mixture of substituted aniline and ammonium thiocyanate in acidic aqueous solution according to the reported method [51].

#### 3.1.1. Synthesis of 1-(1H-Benzo[d][1,2,3]triazol-1-yl)-2-chloroethanone

A mixture of benzotriazole (0.01 mol, 1.19 g) and anhydrous sodium acetate (0.01 mol, 0.82 g) in dry acetone and chloroacetyl chloride (0.01 mol, 0.79 mL) was added drop-wise. The reaction mixture was stirred for 3 h. At the end of the reaction, the content was poured onto ice water, filtered, dried and recrystallized with ethanol. This yielded white crystal (82%), m.p. (73–75 °C) [52].

#### 3.1.2. General Procedure for the Synthesis of 4-(1H-Benzo[d][1,2,3]triazol-1-yl)-1-aryl-1H-imidazole-2(3H)-thione (BI1-12)

1-(1H-benzo[d][1,2,3]triazol-1-yl)-2-chloroethanone (0.01 mole), anhydrous sodium acetate and substituted thiourea T1-12 (0.01 mole) were dissolved in ethanol, and the mixture was refluxed for 6 h. The mixture was poured into cold water and the solid formed was recrystallized using ethanol to afford the final compounds **BI1-12** which showed one spot in the TLC technique [53].

*4-(1H-Benzo[d][1,2,3]Triazol-1-yl)-1-Phenyl-1H-Imidazole-2(3H)-Thione* (**BI1**), Yield: 55%; m.p.: 175–177 °C; IR (KBr) cm^−1^: 3270 (NH), 3120,3050 (arom.CH), 2957 (aliph.CH), 1640 (C=N), 1610 (C=C); ^1^HNMR (DMSO-*d*_6_, D_2_O) δ: 4.01 (s, 2H, CH_2_), 6.99 (d, 2H, arom.CH), 7.15 (d,2H, arom.CH), 7.37 (d,4H, arom.CH), 7.69–7.70 (s,1H, arom.CH, and 1H, CH=), 11.48 (s, brd, 1H, NH, D_2_O exchangeable); ^13^C NMR (DMSO-*d*_6_) δ: 35.59 (CH_2_,C5, imidazole), 120.81 (C4,C7, benzotriazole), 122.17 (C2,C6,benzene), 125.36 (C5, benzotriazole and C4, benzene), 129.52 (C3,C5, benzene), 129.82 (C6,benzotriazole), 139.10 (C7a, benzotriazole), 146.05 (C3a, benzotriazole), 177.70 (C1, benzene), 179.02 (C4, imidazole), 189.07 (C2, C=S, imidazole); MS: *m*/*z* (%abundance) 293.35 (M^+^) (12.78); Anal. Calcd. For C_15_H_11_N_5_S: C, 61.42; H, 3.78; N, 23.87; Found C, 61.93; H, 3.64; N, 23.51.

*4-(1H-Benzo[d][1,2,3]Triazol-1-yl)-1-(4-Methylphenyl)-1H-Imidazole-2(3H)-Thione* (**BI2**), Yield: 63%; m.p.: 178–180 °C; IR (KBr) cm^−1^: 3270 (NH), 3120,3050 (arom.CH), 2957 (aliph.CH), 1640 (C=N), 1610 (C=C); ^1^HNMR (DMSO-*d*_6_, D_2_O) δ: 2.28 (s, 3H, CH_3_), 3.99 (s, 2H, CH_2_), 6.91 (s, 1H, arom.CH), 6.93 (s,1H, arom.CH), 7.18 (d,2H, arom.CH), 7.20 (d,2H, arom.CH), 7.57 (s,1H, arom.CH), 7.57–7.59 (s,2H,1H, arom.CH and1H, CH=), 11.09 (s, brd, 1H, NH, D_2_O exchangeable); ^13^C NMR (DMSO-*d*_6_) δ: 20.94 (CH_3_), 35.64 (CH_2_,C5, imidazole), 120.64 (C2,C6, benzene), 122.14 (C7,benzotriazole), 129.85 (C4, benzotriazole), 130.23 (C4, benzene and C5, benzotriazole), 134.32 (C3,C5,benzene), 134.49 (C6, benzotriazole), 136.86 (C3a,C7a, benzotriazole), 137.00 (C1,benzene), 178.26 (C4, imidazole), 188.57 (C2, C=S, imidazole); MS: *m*/*z* ( %abundance) 307.37 (M^+^) (14.43); Anal. Calcd. For C_16_H_13_N_5_S: C, 62.52; H, 4.26; N, 22.78; Found C, 62.23; H, 4.54; N, 22.49.

*4-(1H-Benzo[d][1,2,3]Triazol-1-yl)-1-(4-Ethylphenyl)-1H-Imidazole-2(3H)-Thione* (**BI3**), Yield: 73%; m.p.: 150–152 °C; IR (KBr) cm^−1^: 3307 (NH), 3104,3020 (arom.CH), 2952 (aliph.CH), 1655 (C=N), 1620 (C=C); ^1^HNMR (DMSO-*d*_6_, D_2_O) δ: 1.17 (t, 3H, CH_3_), 2.61 (q, 2H, CH_2_), 4.00 (s, 2H, CH_2_), 6.94 (s, 1H, arom.CH), 6.95 (s,1H, arom.CH), 7.21 (d,2H, arom.CH), 7.23 (d,2H, arom.CH), 7.58 (s,1H, arom.CH), 7.60 (s,1H, arom.CH), 7.62 (s,1H, CH=),11.09 (s, brd, 1H, NH, D_2_O exchangeable); ^13^C NMR (DMSO-*d*_6_) δ: 16.06 (CH_3_),28.07 (CH_2_,C5, imidazole), 120.78 (C7, benzotriazole), 122.15 (C4,benzotriazole), 122.19 (C5,benzotriazole), 128.68 (C6,benzotriazole), 129.04 (C3,C5,benzene), 131.35 (C7a, benzotriazole), 136.95 (C4, benzene), 137.03 (C1, benzene), 140.74 (C2,C6,benzene),159.47 (C3a,benzotriazole), 178.33 (C4, imidazole), 188.58 (C2, C=S, imidazole); MS: *m*/*z* (%abundance) 321.40 (M^+^) (31.00); Anal. Calcd. For C_17_H_15_N_5_S: C, 63.53; H, 4.70; N, 21.79; Found C, 63.39; H, 4.48; N, 21.47.

*4-(1H-Benzo[d][1,2,3]Triazol-1-yl)-1-(4-Methoxyphenyl)-1H-Imidazole-2(3H)-Thione* (**BI4**), Yield: 72%; m.p.: 175–177 °C; IR (KBr) cm^−1^: 3317 (NH), 3124,3020 (arom.CH), 2922 (aliph.CH), 1660 (C=N), 1620,1616 (C=C); ^1^HNMR (DMSO-*d*_6_, D_2_O) δ: 3.75 (s, 3H, OCH_3_), 3.99 (s, 2H, CH_2_), 6.91 (d, 2H, arom.CH), 6.94 (d,2H, arom.CH), 6.96 (s,1H, arom.CH), 7.00 (s,1H, arom.CH), 7.02 (s,1H, arom.CH), 7.59 (s, 1H, arom.CH), 7.61 (s,1H, CH=), 11.04 (s, brd, 1H, NH, D_2_O exchangeable); ^13^C NMR (DMSO-*d*_6_) δ: 36.12 (CH_2_,C5, imidazole), 55.74 (CH_3_),114.43 (C3,C5 benzene), 114.56(C7,benzotriazole),114.98(C4,benzotriazole),122.27(C2,C6,benzene),124.01(C5,benzotriazole),126.01(C6,benzotriazole),132.50(C7a,benzotriazole),156.72 (C1,benzene,C3a,benzotriazole), 157.30 (C4 benzene), 177.97 (C4, imidazole), 188.47 (C2, C=S, imidazole); MS: *m*/*z* (%abundance) 323.37 (M^+^) (32.94); Anal. Calcd. For C_16_H_13_N_5_OS: C, 59.43; H, 4.05; N, 21.66; Found C, 59.61; H, 4.19; N, 21.38.

*4-(1H-Benzo[d][1,2,3]Triazol-1-yl)-1-(4-Hydroxyphenyl)-1H-Imidazole-2(3H)-Thione* (**BI5**), Yield: 68%; m.p.: 267–269 °C; IR (KBr) cm^−1^: 3422 (OH),3270 (NH), 3114,3020 (arom.CH), 2960 (aliph.CH), 1654 (C=N), 1620 (C=C); ^1^HNMR (DMSO-*d*_6_, D_2_O) δ: 3.90 (s, 2H, CH_2_), 6.76 (d,2H, arom.CH), 6.78 (d,2H, arom.CH), 6.93 (s,1H, arom.CH), 6.95 (s,1H, arom.CH), 7.46 (s,1H, arom.CH),7.48 (s,1H, arom.CH), 7.96 (s,1H, CH=), 9.47 (s, brd, 1H, OH, D_2_O exchangeable) 11.06 (s, brd, 1H, NH, D_2_O exchangeable); ^13^C NMR (DMSO-*d*_6_) δ: 38.87 (CH_2_,C5,imidazole),115.77(C7,benzotriazoleand C3,C5,benzene), 116.24 (C4,C5,benzotriazole), 122.47 (C2,C6,benzene), 124.68 (C6,benzotriazole), 131.10 (C7a, benzotriazole), 134.66 (C1, benzene), 155.01(C4,benzene),155.86 (C3a,benzotriazole), 177.70 (C4, imidazole), 188.43 (C2, C=S, imidazole); MS: *m*/*z* (%abundance) 309.35 (M^+^) (15.47); Anal. Calcd. For C_15_H_11_N5OS: C, 58.24; H, 3.58; N, 22.64; Found C, 58.41; H, 3.81; N, 22.42.

*4-(1H-Benzo[d][1,2,3]Triazol-1-yl)-1-(4-Chlorophenyl)-1H-Imidazole-2(3H)-Thione* (**BI6**), Yield: 70%; m.p.: 187–189 °C; IR (KBr) cm^−1^: 3315 (NH), 3070,3010 (arom.CH), 2910 (aliph.CH), 1662 (C=N), 1620 (C=C); ^1^HNMR (DMSO-*d*_6_, D_2_O) δ: 3.99 (s, 2H, CH_2_), 6.99 (s, 1H, arom.CH), 7.39 (d,2H, arom.CH), 7.44 (s,1H, arom.CH), 7.48 (d,2H, arom.CH), 7.73 (d,2H, arom.CH), 8.00 (s,1H, CH=), 11.27 (s, brd, 1H, NH, D_2_O exchangeable); ^13^C NMR (DMSO-*d*_6_) δ: 34.97 (CH_2_,C5, imidazole), 122.23 (C5,C7, benzotriazole), 123.06 (C4,C6,benzotriazole), 123.56 (C4, benzene), 128.90 (C5,benzene), 129.42 (C6,benzene), 129.67 (C1,C2, benzene), 138.17 (C7a, benzotriazole,C3, benzene), 146.74 (C3a,benzotriazole), 178.80 (C4, imidazole), 188.55 (C2, C=S, imidazole); MS: *m*/*z* (%abundance) 327.79 (M^+^) (43.85); Anal. Calcd. For C_15_H_10_ClN_5_S: C, 54.96; H, 3.07; N, 21.37; Found C, 54.66; H, 3.21; N, 21.23.

*4-(1H-Benzo[d][1,2,3]Triazol-1-yl)-1-(2-Chlorophenyl)-1H-Imidazole-2(3H)-Thione* (**BI7**), Yield: 70%; m.p.: 180–182 °C; IR (KBr) cm^−1^: 3312 (NH), 3110,3020 (arom.CH), 2960 (aliph.CH), 1652 (C=N), 1620 (C=C); ^1^HNMR (DMSO-*d*_6_, D_2_O) δ: 3.65 (s,2H, CH_2_), 5.38 (s, brd,1H, NH, D_2_O exchangeable), 6.96 (s, 2H, arom.CH), 7.24 (s,1H, arom.CH), 7.46 (s,3H, arom.CH), 7.87 (s,3H, arom.CH), 8.20 (s,1H, CH=); ^13^C NMR (DMSO-*d*_6_) δ: 34.20 (CH_2_,C5, imidazole), 115.08 (C7, benzotriazole), 115.59 (C4,benzotriazole), 119.41 (C5, benzotriazole), 121.83 (C5,benzene), 124.49 (C6,benzotriazole), 126.86 (C6 benzene), 127.45 (C3, benzene), 127.68 (C4, benzene), 129.27 (C7a, benzotriazole), 129.83(C1,benzene),136.73 (C3a,benzotriazole), 140.61 (C2, benzene), 152.08 (C4, imidazole), 182.70 (C2, C=S, imidazole); MS: *m*/*z* (%abundance) 327.79 (M^+^) (4.85); Anal. Calcd. For C_15_H_10_ClN_5_S: C, 54.96; H, 3.07; N, 21.37; Found C, 54.73; H, 3.37; N, 21.54.

*4-(1H-Benzo[d][1,2,3]Triazol-1-yl)-1-(3-Chlorophenyl)-1H-Imidazole-2(3H)-Thione* (**BI8**), Yield: 65%; m.p.: 120–122 °C; IR (KBr) cm^−1^: 3340 (NH), 3120,3070 (arom.CH), 2980 (aliph.CH), 1650 (C=N), 1620 (C=C); ^1^HNMR (DMSO-*d*_6_, D_2_O) δ: 4.00 (s, 2H, CH_2_), 7.14 (s, 1H, arom.CH), 7.15 (d,2H, arom.CH), 7.32 (s,1H, arom.CH), 7.33 (d,2H, arom.CH), 7.36 (s, 1H, arom.CH), 7.71 (d,2H, arom.CH), 9.81 (s,1H, CH=), 11.46 (s, brd, 1H, NH, D_2_O exchangeable); ^13^C NMR (DMSO-*d*_6_) δ: 34.86 (CH_2_,C5, imidazole), 120.01 (C7, benzotriazole), 121.52 (C4,benzotriazole), 122.58 (C5, benzotriazole), 124.24 (C4,benzene), 125.83 (C6,benzotriazole), 129.80 (C5,C6 benzene), 130.63 (C7a, benzotriazole), 133.16 (C3, benzene), 141.31 (C1,C2,benzene),146.36 (C3a,benzotriazole), 149.42 (C4, imidazole), 181.66 (C2, C=S, imidazole); MS: *m*/*z* (%abundance) 327.79 (M^+^) (40.61); Anal. Calcd. For C_15_H_10_ClN_5_S: C, 54.96; H, 3.07; N, 21.37; Found C, 54.53; H, 3.34; N, 21.11.

*4-(1H-Benzo[d][1,2,3]Triazol-1-yl)-1-(2,4-Dichlorophenyl)-1H-Imidazole-2(3H)-Thione* (**BI9**), Yield: 70%; m.p.: 187–189 °C; IR (KBr) cm^−1^: 3324 (NH), 3050 (arom.CH), 2964 (aliph.CH), 1640 (C=N), 1610 (C=C); ^1^HNMR (DMSO-*d*_6_, D_2_O) δ: 4.89 (s, brd, 1H, NH, D_2_O exchangeable),5.07 (s, 2H, CH_2_), 7.17–7.20 (m, 4H, arom.CH), 7.80–7.81 (m,3H, arom.CH), 7.90–8.00 (s, brd,1H, CH=); ^13^C NMR (DMSO-*d*_6_) δ: 52.47 (CH_2_,C5, imidazole), 111.89 (C7, benzotriazole), 115.83 (C4,benzotriazole), 118.13 (C5, benzotriazole), 119.07 (C5,benzene), 123.04 (C6,benzotriazole), 123.74(C6,benzene),126.11(C3,benzene), 126.84(C4,benzene),133.97(C7a,benzotriazole),142.21(C2,benzene),145.56(C1,benzene), 148.62 (C3a,benzotriazole), 169.20 (C4, imidazole), 187.29 (C2, C=S, imidazole); MS: *m*/*z* (%abundance) 362.24 (M^+^) (42.61); Anal. Calcd. For C_15_H_9_Cl_2_N_5_S: C, 49.74; H, 2.50; N, 19.33; Found C, 49.61; H, 2.24; N, 19.28.

*4-(1H-Benzo[d][1,2,3]Triazol-1-yl)-1-(2-Florophenyl)-1H-Imidazole-2(3H)-Thione* (**BI10**), Yield: 70%; m.p.: 196–198 °C; IR (KBr) cm^−1^: 3311 (NH), 3112,3020 (arom.CH), 2970 (aliph.CH), 1640 (C=N), 1620 (C=C); ^1^HNMR (DMSO-*d*_6_, D_2_O) δ: 3.94 (s, 2H, CH_2_), 6.11 (s, brd, 1H, NH, D_2_O exchangeable), 7.28 (s, 1H, arom.CH), 7.29 (s,1H, arom.CH), 7.30 (s,1H, arom.CH), 7.31 (s,1H, arom.CH), 7.84 (s,1H, arom.CH), 7.85 (s,1H, arom.CH), 7.85 (s,1H, arom.CH), 7.86 (s,1H, arom.CH), 7.96 (s,1H, CH=); ^13^C NMR (DMSO-*d*_6_) δ: 34.22 (CH_2_,C5, imidazole), 115.63 (C7,benzotriazole),116.04 (C4,C5,benzotriazole), 116.18 (C5, benzene), 123.96 (C6,benzene), 124.62 (C6,benzotriazole), 124.73 (C3,C4 benzene), 128.68 (C7a, benzotriazole),139.41 (C1,benzene), 139.53(C2,benzene),140.83 (C3a,benzotriazole), 170.10(C4, imidazole), 184.64 (C2, C=S, imidazole); MS: *m*/*z* (%abundance) 311.34 (M^+^) (34.83); Anal. Calcd. For C_15_H_10_FN_5_S: C, 57.87; H, 3.24; N, 22.49; Found C, 57.71; H, 3.47; N, 22.24.

*4-(1H-Benzo[d][1,2,3]Triazol-1-yl)-1-(4-Bromophenyl)-1H-Imidazole-2(3H)-Thione* (**BI11**), Yield: 68%; m.p.: 267–269 °C; IR (KBr) cm^−1^: 3205 (NH), 3140,3020 (arom.CH), 2930 (aliph.CH), 1650 (C=N), 1620 (C=C); ^1^HNMR (DMSO-*d*_6_, D_2_O) δ: 4.02 (s, 2H, CH_2_), 6.92–6.93 (d, 2H, arom.CH), 7.44–7.52 (m,4H,arom.CH), 7.54–7.67 (d,2H, arom.CH), 7.91 (s,1H, CH=), 11.42 (s, brd, 1H, NH, D_2_O exchangeable); ^13^C NMR (DMSO-*d*_6_) δ: 34.95 (CH_2_,C5, imidazole), 117.02 (C7,C4,benzotriazole), 122.58 (C5,benzotriazole,C4,benzene), 123.97 (C6,benzotriazole), 132.17 (C7a,benzotriazole), 132.33 (C3,C5 benzene), 132.59 (C1, benzene), 138.65(C2,C6,benzene),147.24 (C3a,benzotriazole), 175.35 (C4, imidazole), 188.46 (C2, C=S, imidazole); MS: *m*/*z* (%abundance) 372.37 (M^+^) (17.82); Anal. Calcd. For C_15_H_10_BrN_5_S: C, 48.40; H, 2.71; N, 18.81; Found C, 48.11; H, 2.54; N, 18.69.

*4-(4-(1H-Benzo[d][1,2,3]Triazol-1-yl)-2-Thioxo-2,3-Dihydro-1H-Imidazol-1-yl)Benzenesulfonamide* (**BI12**), Yield: 64%; m.p.: 217–219 °C; IR (KBr) cm^−1^: 3368,3311,3270 (NH), 3092,3050 (arom.CH), 2980 (aliph.CH), 1650 (C=N), 1640 (C=C); ^1^HNMR (DMSO-*d*_6_, D_2_O) δ: 4.03 (s, 2H, CH_2_), 7.11 (d,2H, arom.CH), 7.30 (d,2H, arom.CH), 7.32 (s,2H, SO_2_NH_2_, D_2_O exchangeable), 7.80 (d,2H, arom.CH), 7.82 (s,3H, 2H,arom.CH and 1H, CH=), 11.75 (s, brd, 1H, NH, D_2_O exchangeable); ^13^C NMR (DMSO-*d*_6_) δ:34.87 (CH_2_,C5,imidazole), 120.42 (C4,C7,benzotriazole), 121.84 (C2,C6,benzene), 122.18 (C5,C6, benzotriazole), 127.52 (C3,C5, benzene), 127.88 (C4,benzene and C7a,benzotriazole), 140.10 (C3a, benzotriazole and C1,benzene), 174.84 (C4, imidazole), 188.15 (C2, C=S, imidazole); MS: *m*/*z* (%abundance) 372.42 (M^+^) (69.19); Anal. Calcd. For C_15_H_12_N_6_O_2_S_2_: C, 48.38; H, 3.25; N, 22.57; Found C, 48.31; H, 3.44; N, 22.68.

### 3.2. Biochemical Evaluation of Activity

All biochemical assays were accomplished in triplicate on a minimum of three independent times for the calculation of the mean values and reporting.

#### 3.2.1. Cell Culture

All the human tumour cell lines MCF-7, HL-60, HCT-116, and HUVEC were cultured in Dulbecco’s Modified Eagle’s Medium (DMEM) with 10% fetal bovine serum, 2 mM L-glutamine and 100 µg/mL penicillin/streptomycin. Cells were maintained at 37 °C in 5% CO_2_ in a humidified incubator. All cells were sub-cultured three times/week by trypsinization using TrypLE Express (1X).

#### 3.2.2. Cell Viability Assay

The Benzotriazole compounds were evaluated for antiproliferative effect using the MTT viability assay of various cancer cell lines (MCF-7, HCT-116 and HL-60) and the normal human umbilical vein endothelial cell line (HUVEC) to calculate the relative IC_50_ values for each compound. Cells were seeded in triplicate in 96-well plates at a density of 10 × 10^3^ cells/mL in a total volume of 200 µL per well. 0.1% of DMSO was used as a vehicle control. After this time, they were treated with 2 µL test compound which had been pre-prepared as stock solutions in ethanol to furnish the concentration range of study, 10 nM to 100 µM, and re-incubated for a further 72 h. The culture medium was then removed, and the cells washed with 100 µL phosphate buffered saline (PBS) and 50 µL MTT added, to reach a final concentration of 1 mg/mL MTT added. Cells were incubated for 2 h in darkness at 37 °C. At this point solubilisation was begun through the addition of 200 mL DMSO and the cells maintained at room temperature in darkness for 20 min to ensure thorough colour diffusion before reading the absorbance. Plates were incubated for 72 h at 37 °C + 5% CO_2_. The MTT (5 mg/mL in PBS) was added and incubated for another 4 h, and the optical density was detected with a microplate reader at 570 nm. Results were expressed as percentage viability relative to vehicle control (100%). Dose response curves were plotted and IC_50_ values (concentration of drug resulting in 50% reduction in cell survival) were obtained using the commercial software package Prism (GraphPad Software, Inc., La Jolla, CA, USA). All the experiments were repeated in at least three independent experiments.

#### 3.2.3. Tubulin Polymerization Assay

The assembly of purified bovine tubulin was monitored using a kit, BK006, purchased from Cytoskeleton Inc., (Denver, CO, USA). The assay was carried out in accordance with the manufacturer’s instructions using the standard assay conditions [54]. Briefly, purified (>99%) bovine brain tubulin (3 mg/mL) in a buffer consisting of 80 mM PIPES (pH 6.9), 0.5 mM EGTA, 2 mM MgCl_2_, 1 mM GTP and 10% glycerol was incubated at 37 °C in the presence of either vehicle (2% (*v/v*) ddH_2_O), CA-4, benzotriazole compounds. Light was scattered proportionally to the concentration of polymerized microtubules in the assay. Therefore, the tubulin assembly was monitored turbidimetrically at 340 nm in a Spectramax 340 PC spectrophotometer (Molecular Devices, Sunnyvale, CA, USA). The concentration that inhibits tubulin polymerization by 50% (IC_50_) was determined using area under the curve (AUC). The AUC of the untreated controls were considered as 100% polymerization. The IC_50_ value for each compound was computed using GraphPad Prism Software.

#### 3.2.4. Colchicine Site Competitive Binding Assay

The affinity of Benzotriazole compounds to colchicine binding site was determined using a Colchicine Site Competitive Assay kit CytoDYNAMIX Screen15 (Cytoskeleton, Inc., Denver, CO, USA) using the standard protocol of the manufacturer to determine Ki values (μM). Biotin-labelled tubulin (0.5 μg) in 10 μL of reaction buffer was mixed with [3H]colchicine (0.08 μM, PerkinElmer, Waltham, MA) and the test compounds (positive control colchicine, negative control vinblastine, G-1, fluorescent G-1, or 2-ME) in a 96-well plate (final volume: 100 μL). After incubating for 2 h at 37 °C with gentle shaking, streptavidin-labelled yttrium SPA beads (80 μg in 20 μL reaction buffer, PerkinElmer, Waltham, MA) were added to each well and incubated for 30 min at 4 °C. The plates were then read on a scintillation counter (Packard Instrument, Topcount Microplate Reader) and the percentage of inhibition was calculated [55,56].

#### 3.2.5. Cell Cycle Analysis

HL-60 cells were seeded at a density of 1 × 10^5^ cells/well in 6-well plates and treated with CA-4 and compound **BI9** (1 µM) for 48 and 72 h. The cells were collected by trypsinization and centrifuged at 800× *g* for 15 min. Cells were washed twice with ice-cold PBS and fixed in ice-cold 70% ethanol overnight at −20 °C. Fixed cells were centrifuged at 800× *g* for 15 min and stained with 50 μg/mL of PI, containing 50 μg/mL of DNase-free RNase A, at 37 °C for 30 min. The DNA content of cells (10,000 cells/experimental group) was analysed by flow cytometer at 488 nm using a FACSCalibur flow cytometer (BD Biosciences, San Jose, CA, USA) and all data were recorded and analysed using the CellQuest Software (Becton-Dickinson).

#### 3.2.6. Annexin V/PI Apoptotic Assay

Apoptotic cell death was detected by flow cytometry using Annexin V and propidium iodide (PI). HL-60 Cells were seeded in 6 well plated at density of 1 × 10^5^ cells/mL and treated with vehicle (0.1% (*v*/*v*) EtOH), positive control (CA-4) or compound **BI9** (1 µM) for 48 and 72 h. Cells were then harvested and prepared for flow cytometric analysis. Cells were washed in 1X binding buffer (20X binding buffer: 0.1M HEPES, pH 7.4; 1.4 M NaCl; 25 mM CaCl_2_ diluted in dH_2_O) and incubated in the dark for 30 min on ice in Annexin V-containing binding buffer [1:100]. Cells were then washed once in binding buffer and then re-suspended in PI-containing binding buffer [1:1000]. Samples were analysed immediately using the BD accuri flow cytometer and prism software for analysis the data. Four populations are produced during the assay Annexin V and PI negative (Q4, healthy cells), Annexin V positive and PI negative (Q3, early apoptosis), Annexin V and PI positive (Q2, late apoptosis) and Annexin V negative and PI positive (Q1, necrosis).

#### 3.2.7. Evaluation of Expression Levels of Anti-Apoptotic Proteins Bcl-2, Pro-Apoptotic Proteins Bcl-2, Bax and PARP Cleavage

HL-60 cells were seeded at a density of 1 × 10^5^ cells/flask in T25 flasks. After 48 h, whole cell lysates were prepared from untreated cells or cells treated with vehicle control (EtOH, 0.1% *v/v*) or compound **BI9** (0.5 and 1 μM). HL-60 cells were harvested in RIPA buffer supplemented with protease inhibitors (Roche Diagnostics), phosphatase inhibitor cocktail 2 (Sigma-Aldrich), and phosphatase inhibitor cocktail 3 (Sigma-Aldrich). Equal quantities of protein (as determined by a BCA assay) were resolved by SDS-PAGE (12%) followed by transfer to PVDF membranes. Membranes were blocked in 5% bovine serum albumin/TBST for 1 h. Membranes were incubated in the relevant primary antibodies at 4 °C overnight, washed with TBST, and incubated in horseradish peroxidase conjugated secondary antibody for 1 h at rt and washed again. Western blot analysis was performed as described above using antibodies directed against BAX [1:1000] (Cell Signaling Technology), PARP [1:500] (Cell Signaling Technology) and Bcl-2 [1:500] (Cell Signaling Technology) followed by incubation with a horseradish peroxidase-conjugated anti-mouse antibody [1:2000] (Promega, Madison, WI, USA). All blots were probed with β-actin antibody [1:5000] (Sigma) to confirm equal loading. Proteins were detected using enhanced chemiluminescent Western blot detection (Clarity Western ECL substrate) (Bio Rad) on the ChemiDoc MP System (Bio Rad). Experiments were performed on three independent occasions.

## 4. Conclusions

This paper described the synthesis of a series of innovative combretastatin-A4 analogues in which the cis-olefinic bridge is exchanged with an imidazol-2-thiones and benzotriazole substituent mimics ring A in CA-4. These compounds displayed encouraging antiproliferative activity versus different cancer cell lines. Between them, compound **BI9**, bearing 2,4-chloro-substituted at phenyl attached imidazol-2-thione, showed strong antiproliferative activity versus numerous cancer cell lines such as MCF-7, HL-60 and HCT-116 with IC_50_ 3.57, 0.40 and 2.63 µM, respectively. Importantly, compound **BI9** reported low cytotoxicity in HUVEC cell lines, demonstrating its superior toxicity to proliferating cancer cells. The mechanism ofaction studies suggested that compound **BI9** induced G_2_/M arrest, apoptosis through PARP cleavage and regulated the expression of pro-apoptotic protein BAX and anti-apoptotic proteins Bcl-2. It exerted anticancer activity by hang-up tubulin polymerization in the colchicine binding site. These results strongly suggest that novel benzotriazole moiety bearing imidazol-2-thiones as CA-4 analogues can be further explored to develop promising anti-cell proliferative agents for the more effective tubulin polymerization inhibitors dealing with cancer.

## Figures and Tables

**Table 1:** Antiproliferative activity of benzotriazole analogues on selected cell lines (IC_50_ [µM]).

Compound No.	R	Antiproliferative Activities IC_50_ µM ^a^			
		MCF-7 ^b^	HL-60 ^c^	HCT-116 ^d^	HUVEC ^e^
**BI1**	H	6.4 ± 0.18	5.23 ± 0.36	17.5 ± 1.13	63 ± 3.02
**BI2**	4-CH_3_	12.5 ± 0.36	37.1 ± 2.54	14.8 ± 0.95	12.2 ± 0.58
**BI3**	4-CH_2_CH_3_	10.6 ± 0.31	3.91 ± 0.27	2.75 ± 0.18	135 ± 6.49
**BI4**	4-OCH_3_	2.29 ± 0.07	22.1 ± 1.52	1.48 ± 0.0.03	117 ± 5.62
**BI5**	4-OH	4.45 ± 0.13	1.18 ± 0.08	2.15 ± 0.14	4.74 ± 0.23
**BI6**	4-Cl	7.29 ± 0.21	3.28 ± 0.22	1.51 ± 0.12	27.4 ± 1.31
**BI7**	2-Cl	15.5 ± 0.44	3.42 ± 0.23	7.38 ± 0.48	17.33 ± 0.35
**BI8**	3-Cl	2.66 ± 0.08	5.12 ± 0.35	2.1 ± 0.14	39.8 ± 1.91
**BI9**	2,4-Cl	3.57 ± 0.16	0.4 ± 0.03	2.63 ± 0.17	118.9 ± 5.91
**BI10**	2-F	21.5 ± 0.61	8.41 ± 0.58	7.02 ± 0.45	10.8 ± 0.52
**BI11**	4-Br	38.2 ± 1.09	11.5 ± 0.11	17.4 ± 1.12	47.8 ± 2.29
**BI12**	4-SO_2_NH_3_	6.1 ± 0.75	0.9 ± 0.06	7.83 ± 0.5	25.1 ± 1.2
**CA-4**		0.58 ± 0.02	0.77 ± 0.05	0.24 ± 0.02	13.6 ± 0.65

^a^ IC_50_—compound concentration required to inhibit cell proliferation by 50%. Data are expressed as the mean ± SD from dose-response curves of three independent experiments. ^b^ MCF-7 is a human breast cancer cell line. ^c^ HL-60 is a human leukemia cancer cell line. ^d^ HCT-116 is a human colon cancer cell line. ^e^ HUVEC is a human umbilical vein endothelial cell line.

**Table 2:** Inhibition of tubulin polymerization and colchicine binding by benzotriazole compounds and CA-4.

Compound	Anti-Tubulin Activity ^a^ IC_50_ ± SD (µM)	Colchicine Binding ^b^ % ± SD	
		1 µM Drug	5 µM Drug
**BI3**	0.928 ± 0.04	65.38 ± 2	86.85 ± 2
**BI5**	0.497 ± 0.03	61.78 ± 2	81.34 ± 2
**BI6**	0.928 ±0.03	63.23 ± 2	77.63 ± 2
**BI7**	0.840 ± 0.02	61.18 ± 2	79.95 ± 2
**BI8**	3.057 ± 9.69	59.74± 2	82.13 ± 2
**BI9**	0.520 ±0.02	49.38 ± 2	66.57 ± 2
**BI12**	0.784 ± 0.02	64.67 ± 2	80.26 ± 2
**CA-4**	0.579 ± 0.01	64.23 ± 0.9	85.63 ± 2

^a^ Inhibition of tubulin polymerization. Tubulin was at 10 µM. ^b^ Inhibition of [^3^H] colchicine binding. Tubulin and colchicine were at 1 and 5 µM concentrations, respectively.

## Data Availability

The data presented in this study are available in this article.

## Supplementary Materials

The following are available online, Figure S1: the ^1^H and ^13^C-NMR spectrum for the prepared compounds **BI1-12.**

## Sample Availability

Samples of the compounds are not available from the authors.
