# Supplementary material for: Design, Synthesis, and Antipoliferative Activities of Novel Substituted Imidazole-Thione Linked Benzotriazole Derivatives
Source: Molecules. 2021 Oct 2;26(19):5983. doi: 10.3390/molecules26195983 (PMC8512560; doi:10.3390/molecules26195983)
Supplement: Supplementary file 1 [file molecules-26-05983-s001.zip › molecules-1376492-supplementary.pdf]

BI 1

Khaled Omar_H_SL1

Microanalytical Unit - FOPCU - NMR laboratory
www.pharma.cu.edu.eg dir-mau.fopcu@pharma.cu.edu.eg

BRUKER

^{1}HNMR

```
Current Data Parameters
NAME      Khaled Omar_H_SL1
EXPNO                    10
PROCNO                    1

F2 - Acquisition Parameters
Date_              20200712
Time                  20.29
INSTRUM               spect
PROBHD    5 mm PABBO BB/
PULPROG                zg30
TD                    65536
SOLVENT                DMSO
NS                       32
DS                        2
SWH            8012.820 Hz
FIDRES         0.122266 Hz
AQ            4.0894465 sec
RG                   169.46
DW               62.400 usec
DE                 6.50 usec
TE                296.0 K
D1           1.00000000 sec
TD0                       1

======== CHANNEL f1 ========
SFO1        400.1924713 MHz
NUC1                     1H
P1                15.00 usec
PLW1        10.39999962 W

F2 - Processing parameters
SI                    65536
SF          400.1900000 MHz
WDW                      EM
SSB       0
LB                  0.30 Hz
GB        0
PC                     1.00
```

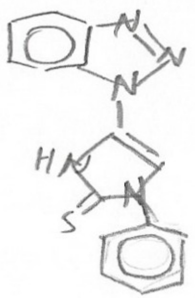

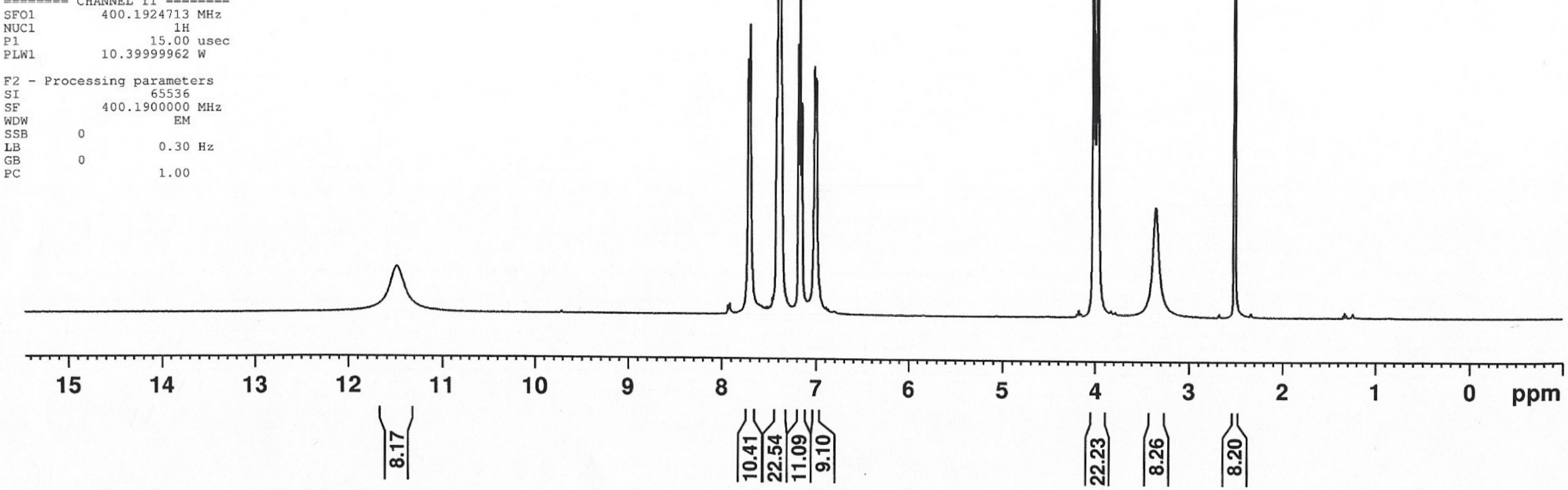

Khaled Omar_H_SL1_D2O

Microanalytical Unit - FOPCU - NMR laboratory
www.pharma.cu.edu.eg dir-mau.fopcu@pharma.cu.edu.eg

BRUKER

BI 1

^{1}HNMR + D2O

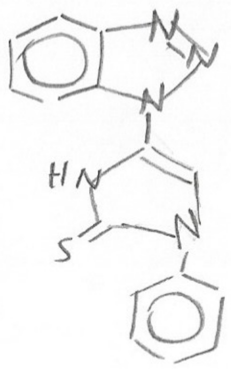

```
Current Data Parameters
NAME      Khaled Omar_H_SL1_D2O
EXPNO                  10
PROCNO                  1

F2 - Acquisition Parameters
Date_            20200818
Time                11.02
INSTRUM             spect
PROBHD   5 mm PABBO BB/
PULPROG              zg30
TD                  65536
SOLVENT              DMSO
NS                     32
DS                      2
SWH              8012.820 Hz
FIDRES           0.122266 Hz
AQ              4.0894465 sec
RG                  22.91
DW                 62.400 usec
DE                   6.50 usec
TE                  298.0 K
D1             1.00000000 sec
TD0                     1

======== CHANNEL f1 ========
SFO1         400.1924713 MHz
NUC1                  1H
P1                 15.00 usec
PLW1         10.39999962 W

F2 - Processing parameters
SI                  65536
SF            400.1900000 MHz
WDW                    EM
SSB      0
LB                   0.30 Hz
GB       0
PC                   1.00
```

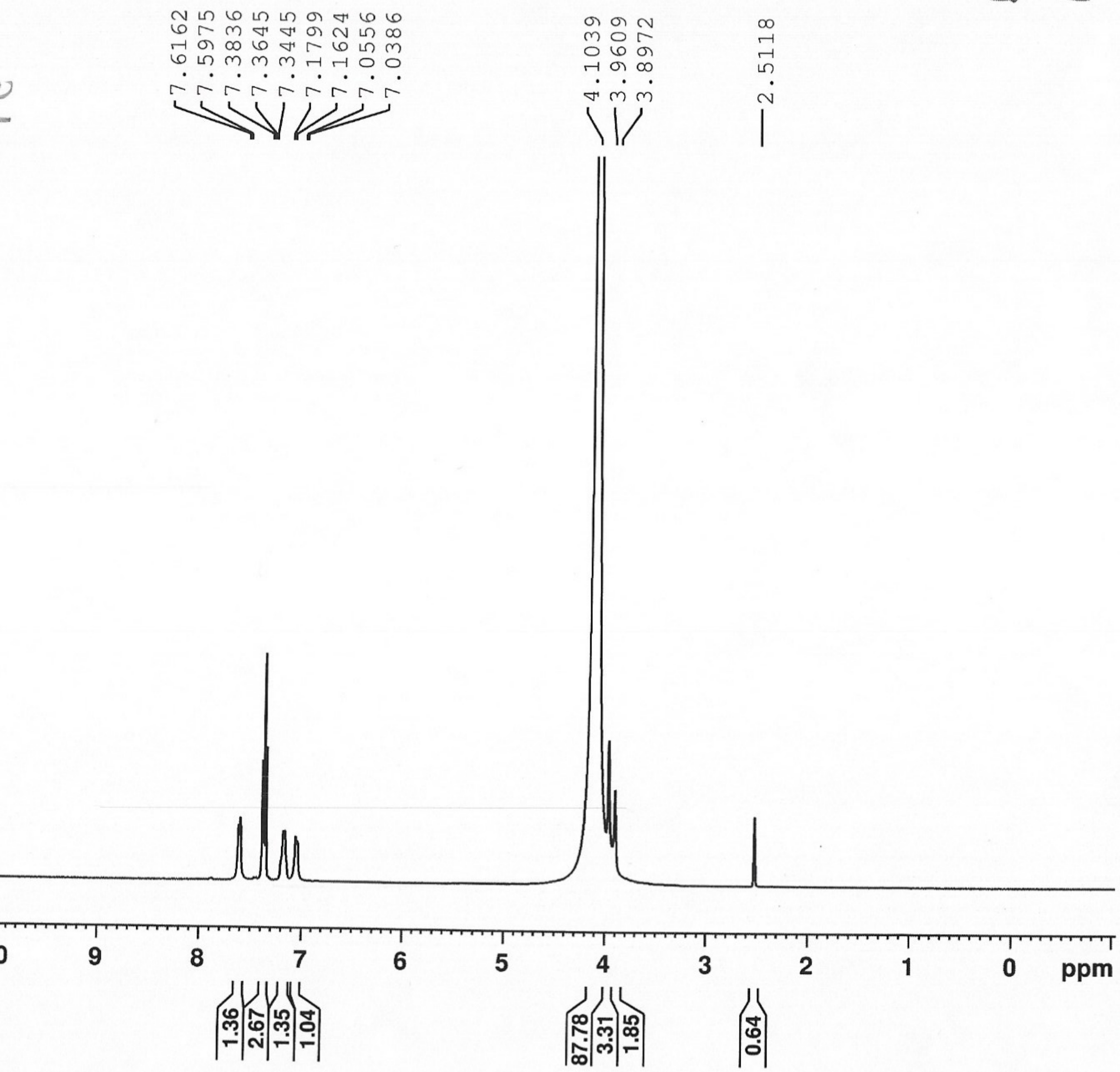

Khaled Omar_C_SL1

BI 1 ^{13}C NMR

Microanalytical Unit - FOPCU - NMR laboratory
www.pharma.cu.edu.eg dir-mau.fopcu@pharma.cu.edu.eg

```
Current Data Parameters
NAME      Khaled Omar_C_SL1
EXPNO                    10
PROCNO                    1

F2 - Acquisition Parameters
Date_              20200817
Time                  17.59
INSTRUM               spect
PROBHD    5 mm PABBO BB/
PULPROG             zgpg30
TD                   65536
SOLVENT               DMSO
NS                    1500
DS                       4
SWH              24038.461 Hz
FIDRES            0.366798 Hz
AQ               1.3631488 sec
RG                  202.37
DW                  20.800 usec
DE                    6.50 usec
TE                   298.0 K
D1              2.00000000 sec
D11             0.03000000 sec
TD0                      1

======== CHANNEL f1 ========
SFO1          100.6379178 MHz
NUC1                  13C
P1                  10.00 usec
PLW1          45.00000000 W

======== CHANNEL f2 ========
SFO2          400.1916008 MHz
NUC2                   1H
CPDPRG[2          waltz16
```

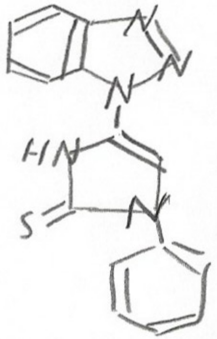

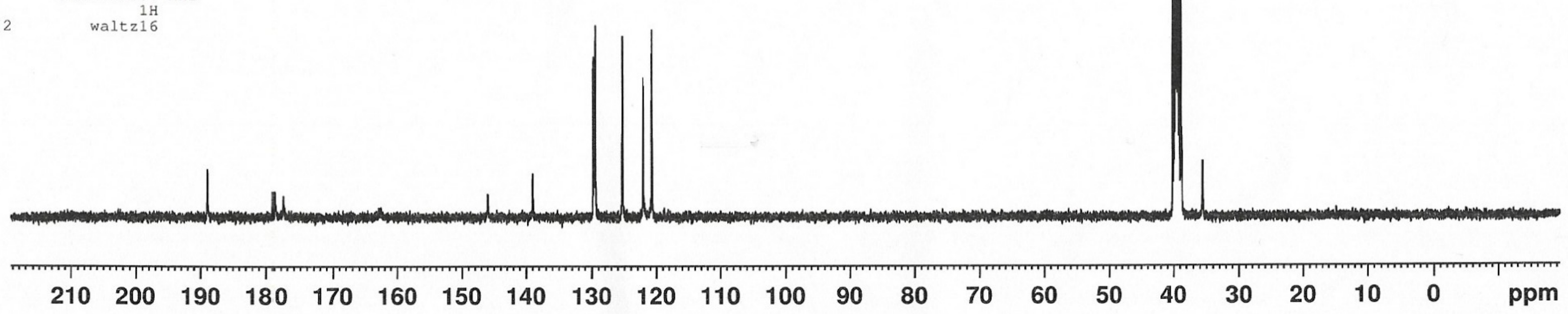

Khaled Omar_H_SL2 BI 2 ^{1}HNMR

Microanalytical Unit - FOPCU - NMR laboratory
www.pharma.cu.edu.eg dir-mau.fopcu@pharma.cu.edu.eg

BRUKER

```
Current Data Parameters
NAME      Khaled Omar_H_SL2
EXPNO                    10
PROCNO                    1

F2 - Acquisition Parameters
Date_              20200817
Time                  18.04
INSTRUM               spect
PROBHD   5 mm PABBO BB/
PULPROG                zg30
TD                    65536
SOLVENT                DMSO
NS                       32
DS                        2
SWH                8012.820 Hz
FIDRES             0.122266 Hz
AQ                4.0894465 sec
RG                   146.06
DW                   62.400 usec
DE                     6.50 usec
TE                    298.0 K
D1               1.00000000 sec
TD0                       1

======== CHANNEL f1 ========
SFO1           400.1924713 MHz
NUC1                     1H
P1                    15.00 usec
PLW1            10.39999962 W

F2 - Processing parameters
SI                    65536
SF             400.1900000 MHz
WDW                      EM
SSB      0
LB                     0.30 Hz
GB       0
PC                     1.00
```

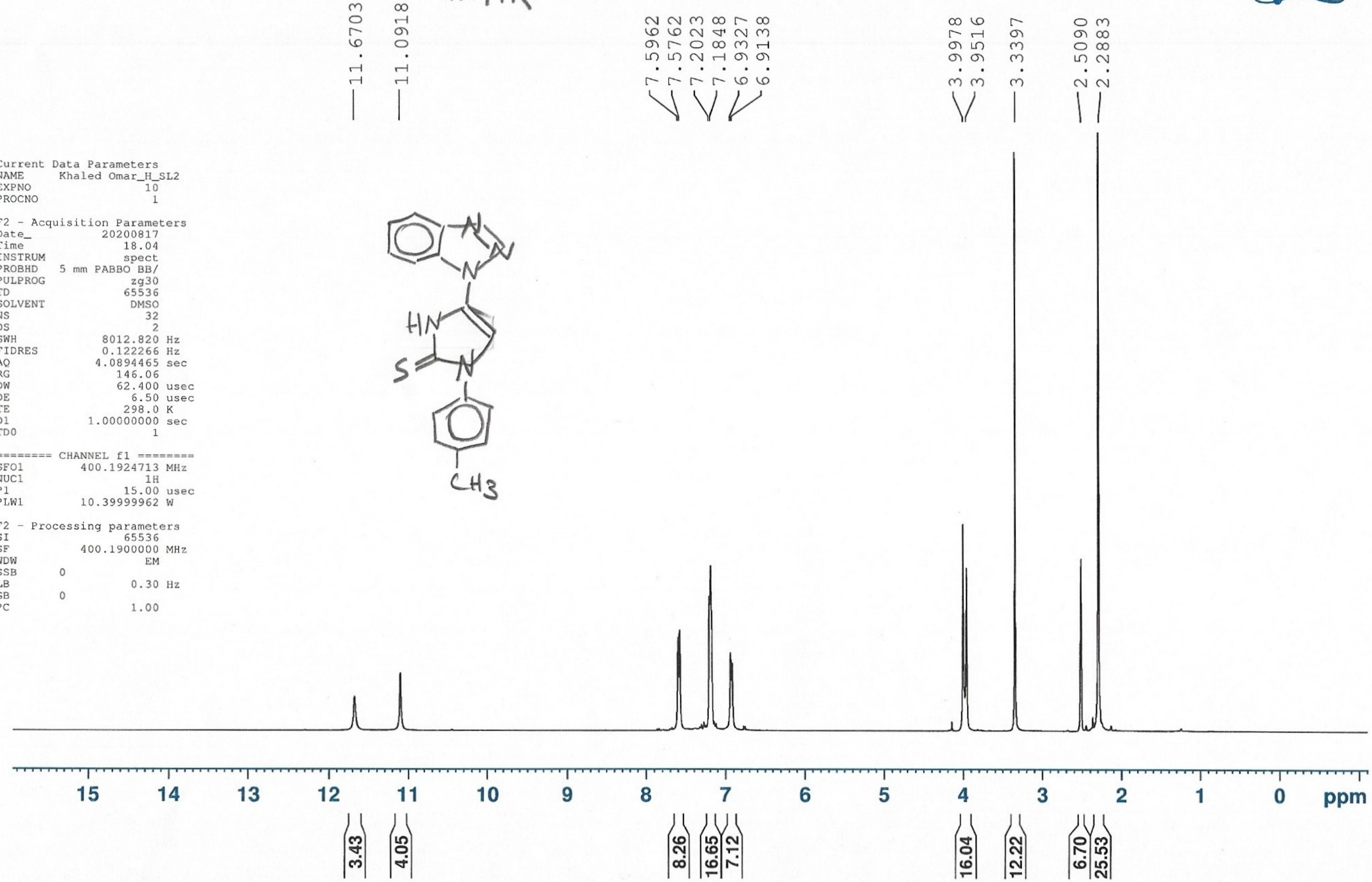

Khaled Omar_H_SL2_D2O BI 2

Microanalytical Unit - FOPCU - NMR laboratory
www.pharma.cu.edu.eg dir-mau.fopcu@pharma.cu.edu.eg

BRUKER

^{1}H NMR + D_2O

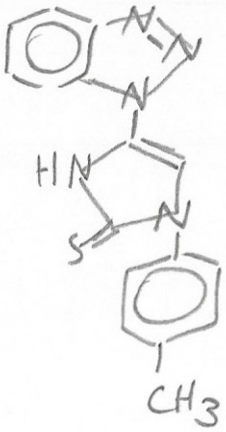


```
Current Data Parameters
NAME      Khaled Omar_H_SL2_D2O
EXPNO                      10
PROCNO                      1

F2 - Acquisition Parameters
Date_            20200818
Time                11.07
INSTRUM             spect
PROBHD   5 mm PABBO BB/
PULPROG              zg30
TD                  65536
SOLVENT              DMSO
NS                     32
DS                      2
SWH              8012.820 Hz
FIDRES           0.122266 Hz
AQ              4.0894465 sec
RG                 146.06
DW                 62.400 usec
DE                   6.50 usec
TE                  298.0 K
D1            1.00000000 sec
TD0                     1

======== CHANNEL f1 ========
SFO1          400.1924713 MHz
NUC1                   1H
P1                  15.00 usec
PLW1          10.39999962 W

F2 - Processing parameters
SI                  65536
SF            400.1900000 MHz
WDW                    EM
SSB      0
LB                   0.30 Hz
GB       0
PC                   1.00
```

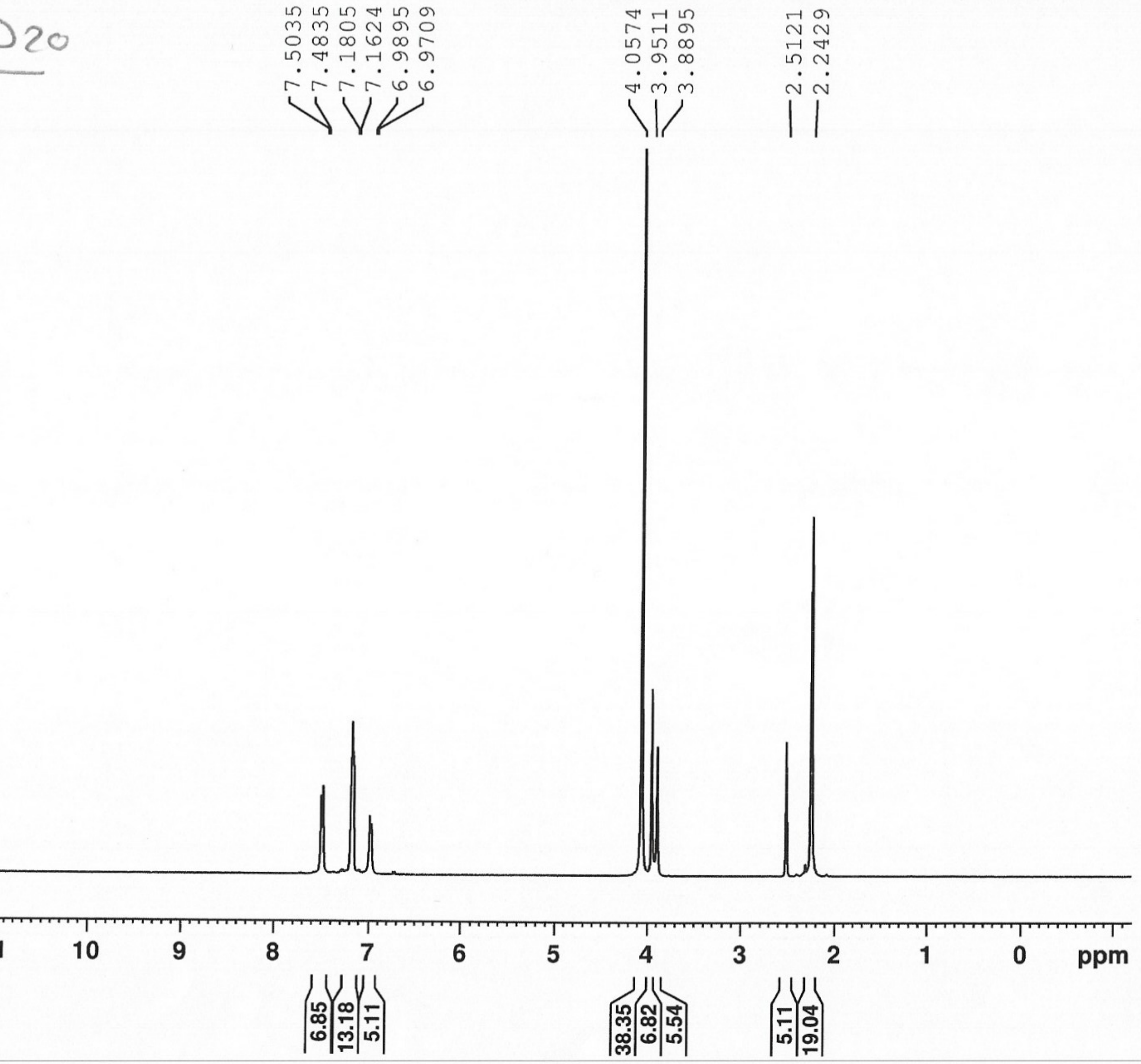

Khaled Omar_C_SL2

BI 2

^{13}C NMR

Microanalytical Unit - FOPCU - NMR laboratory
www.pharma.cu.edu.eg dir-mau.fopcu@pharma.cu.edu.eg

```
Current Data Parameters
NAME      Khaled Omar_C_SL2
EXPNO                   10
PROCNO                   1

F2 - Acquisition Parameters
Date_             20200817
Time                 19.31
INSTRUM              spect
PROBHD   5 mm PABBO BB/
PULPROG            zgpg30
TD                  65536
SOLVENT              DMSO
NS                   1500
DS                      4
SWH             24038.461 Hz
FIDRES           0.366798 Hz
AQ              1.3631488 sec
RG                 202.37
DW                 20.800 usec
DE                   6.50 usec
TE                  298.0 K
D1             2.00000000 sec
D11            0.03000000 sec
TD0                     1

======== CHANNEL f1 ========
SFO1          100.6379178 MHz
NUC1                  13C
P1                  10.00 usec
PLW1          45.00000000 W

======== CHANNEL f2 ========
SFO2          400.1916008 MHz
NUC2                   1H
CPDPRG[2          waltz16
```

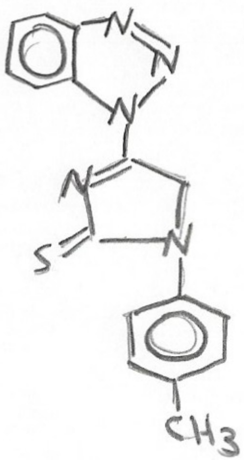

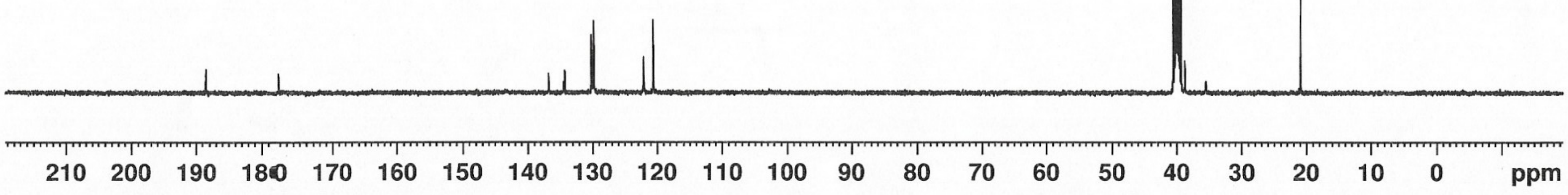

Khaled Omar_H_SL9 BI3

Microanalytical Unit - FOPCU - NMR laboratory
www.pharma.cu.edu.eg dir-mau.fopcu@pharma.cu.edu.eg

'H NMR

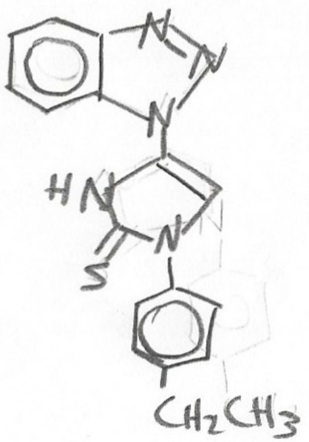

**Current Data Parameters**	
NAME	Khaled Omar_H_SL9
EXPNO	10
PROCNO	1
**F2 - Acquisition Parameters**	
Date_	20200824
Time	20.12
INSTRUM	spect
PROBHD	5 mm PABBO BB/
PULPROG	zg30
TD	65536
SOLVENT	DMSO
NS	32
DS	2
SWH	8012.820 Hz
FIDRES	0.122266 Hz
AQ	4.0894465 sec
RG	180.8
DW	62.400 usec
DE	6.50 usec
TE	298.0 K
D1	1.00000000 sec
TD0	1
**======== CHANNEL f1 ========**	
SFO1	400.1924713 MHz
NUC1	1H
P1	15.00 usec
PLW1	10.39999962 W
**F2 - Processing parameters**	
SI	65536
SF	400.1900000 MHz
WDW	EM
SSB	0
LB	0.30 Hz
GB	0
PC	1.00

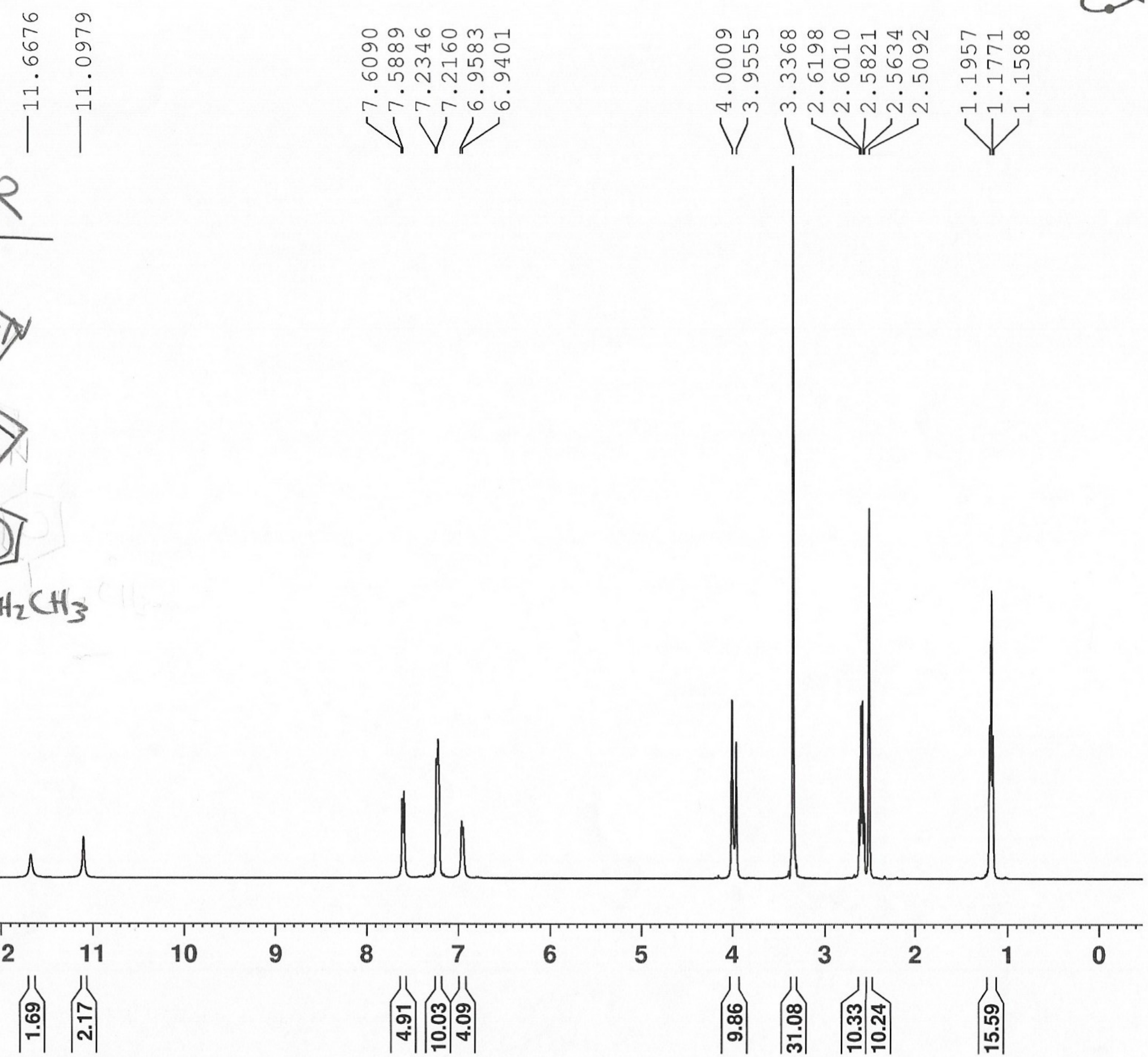

Khaled Omar_H_SL9_D2O

Microanalytical Unit - FOPCU - NMR laboratory
www.pharma.cu.edu.eg dir-mau.fopcu@pharma.cu.edu.eg

BI 3

^{1}HNMR + D_2O

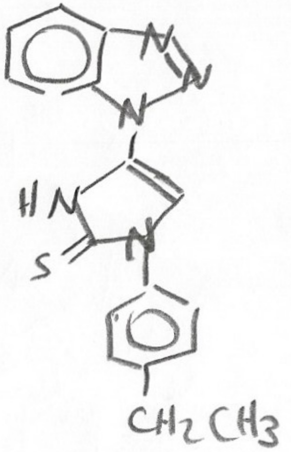


```
Current Data Parameters
NAME      Khaled Omar_H_SL9_D2O
EXPNO                        10
PROCNO                        1

F2 - Acquisition Parameters
Date_              20200825
Time                  12.41
INSTRUM               spect
PROBHD    5 mm PABBO BB/
PULPROG                zg30
TD                    65536
SOLVENT                DMSO
NS                       32
DS                        2
SWH                8012.820 Hz
FIDRES             0.122266 Hz
AQ                4.0894465 sec
RG                   169.46
DW                   62.400 usec
DE                     6.50 usec
TE                    298.0 K
D1               1.00000000 sec
TD0                       1

======== CHANNEL f1 ========
SFO1         400.1924713 MHz
NUC1                      1H
P1                     15.00 usec
PLW1            10.39999962 W

F2 - Processing parameters
SI                    65536
SF             400.1900000 MHz
WDW                      EM
SSB      0
LB                     0.30 Hz
GB       0
PC                     1.00
```

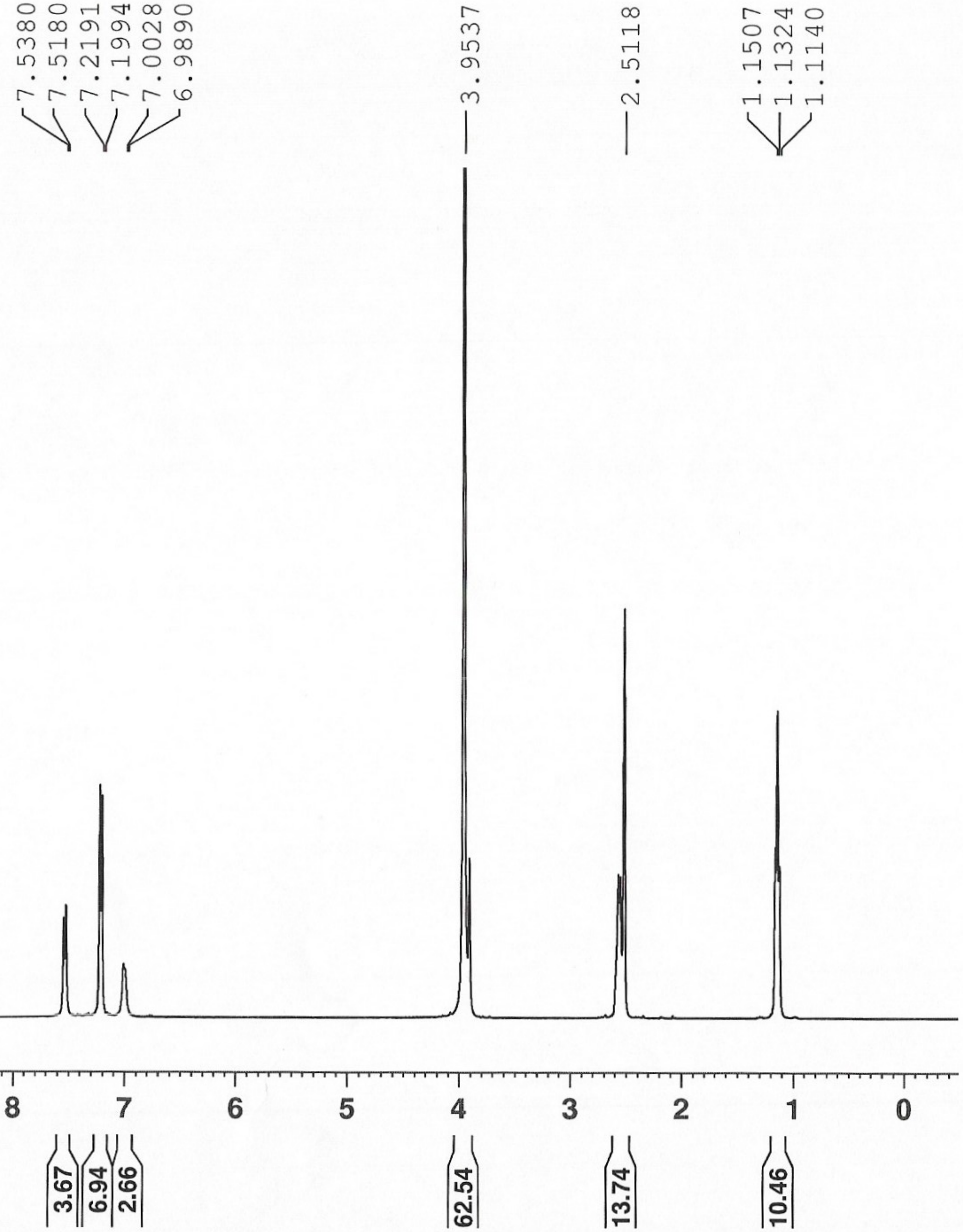

Khaled Omar_C_SL9

Microanalytical Unit - FOPCU - NMR laboratory
www.pharma.cu.edu.eg dir-mau.fopcu@pharma.cu.edu.eg

BI 3
^{13}C NMR

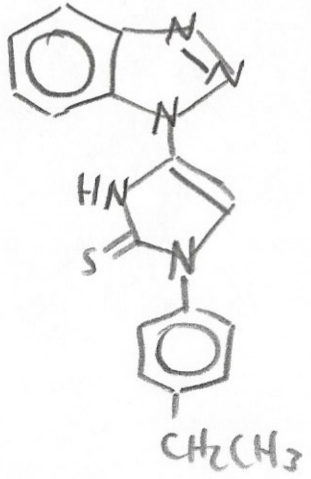


```
Current Data Parameters
NAME      Khaled Omar_C_SL9
EXPNO                    10
PROCNO                    1

F2 - Acquisition Parameters
Date_              20200824
Time                  21.22
INSTRUM               spect
PROBHD   5 mm PABBO BB/
PULPROG             zgpg30
TD                   65536
SOLVENT               DMSO
NS                    1200
DS                       4
SWH              24038.461 Hz
FIDRES            0.366798 Hz
AQ               1.3631488 sec
RG                  202.37
DW                  20.800 usec
DE                    6.50 usec
TE                   298.0 K
D1              2.00000000 sec
D11             0.03000000 sec
TD0                      1

======== CHANNEL f1 ========
SFO1          100.6379178 MHz
NUC1                  13C
P1                  10.00 usec
PLW1          45.00000000 W

======== CHANNEL f2 ========
SFO2          400.1916008 MHz
NUC2                   1H
CPDPRG[2          waltz16
```

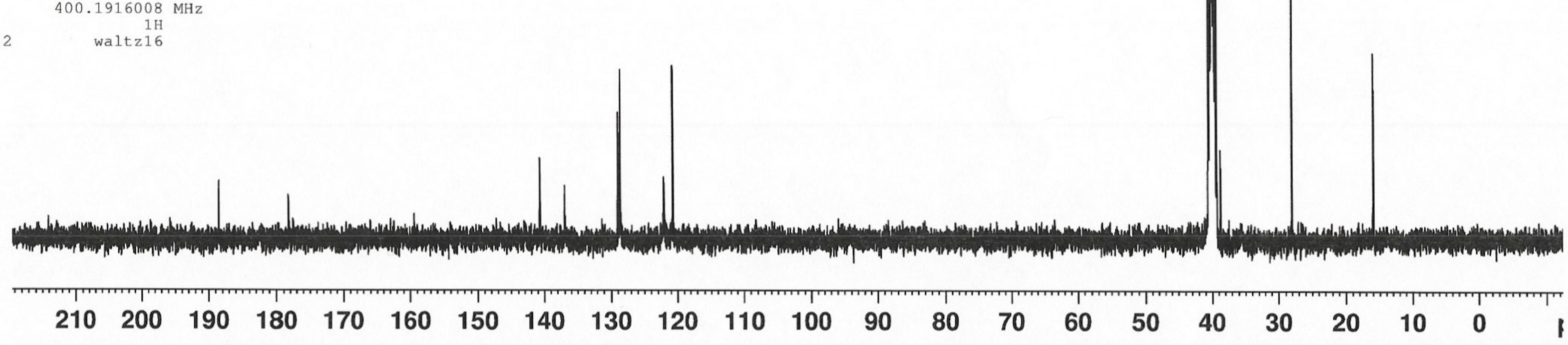

Khaled Omar_H_SL8 BI4

Microanalytical Unit - FOPCU - NMR laboratory
www.pharma.cu.edu.eg dir-mau.fopcu@pharma.cu.edu.eg

¹HNMR

```
Current Data Parameters
NAME      Khaled Omar_H_SL8
EXPNO                    10
PROCNO                    1

F2 - Acquisition Parameters
Date_              20200824
Time                  18.57
INSTRUM               spect
PROBHD   5 mm PABBO BB/
PULPROG                zg30
TD                    65536
SOLVENT                DMSO
NS                       32
DS                        2
SWH               8012.820 Hz
FIDRES            0.122266 Hz
AQ               4.0894465 sec
RG                   106.37
DW                   62.400 usec
DE                     6.50 usec
TE                    298.0 K
D1               1.00000000 sec
TD0                       1

======== CHANNEL f1 ========
SFO1         400.1924713 MHz
NUC1                     1H
P1                    15.00 usec
PLW1           10.39999962 W

F2 - Processing parameters
SI                    65536
SF           400.1900000 MHz
WDW                      EM
SSB       0
LB                     0.30 Hz
GB        0
PC                     1.00
```

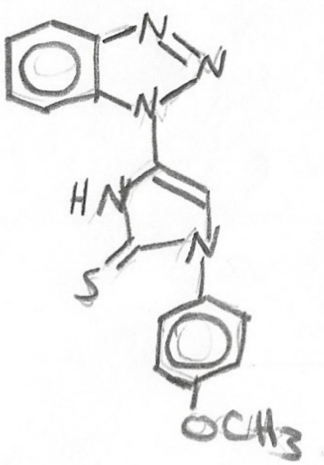

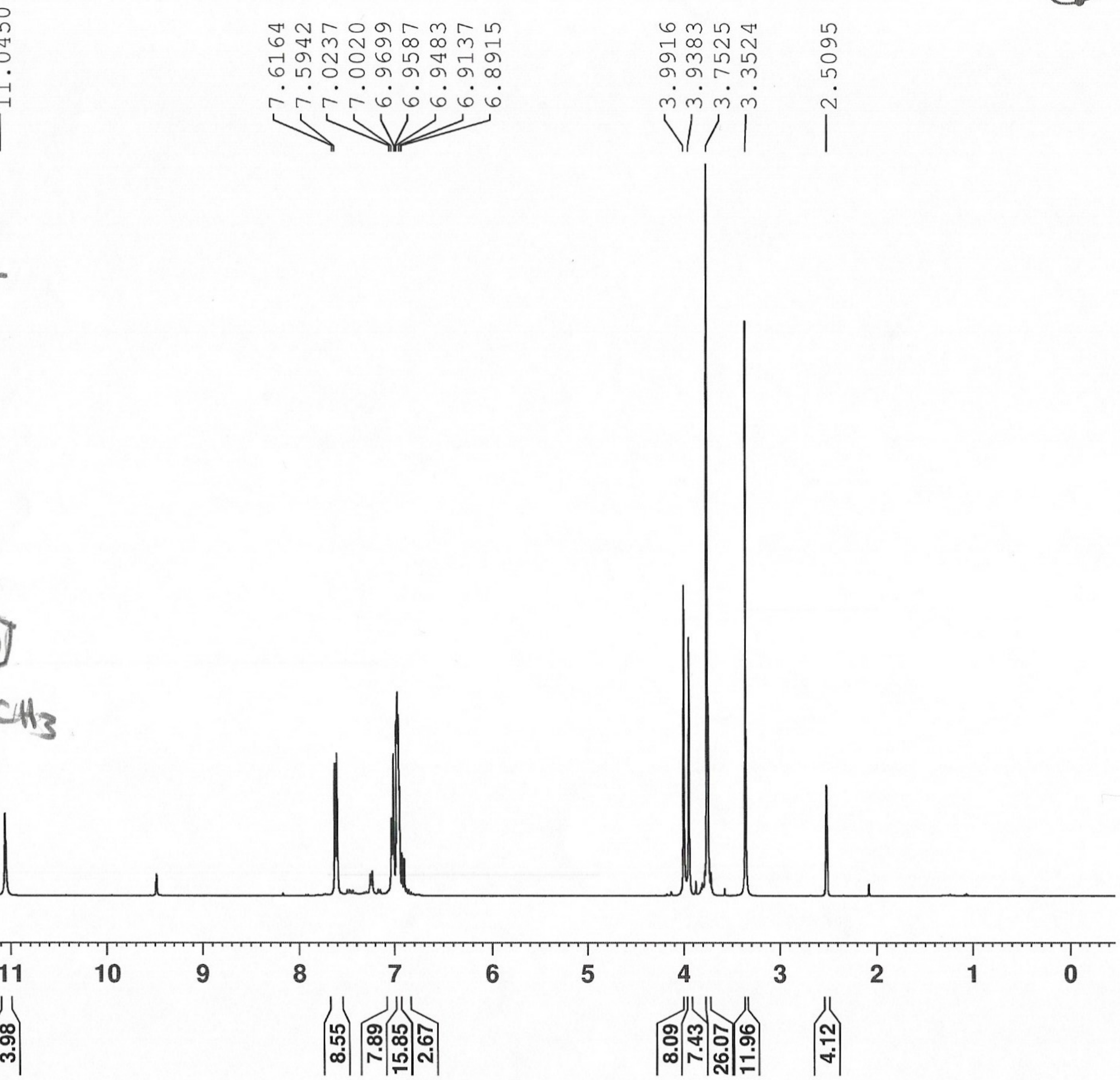

Khaled Omar_H_SL8_D2O

Microanalytical Unit - FOPCU - NMR laboratory
www.pharma.cu.edu.eg dir-mau.fopcu@pharma.cu.edu.eg

BI 4

^{1}HNMR + D_2O

```
Current Data Parameters
NAME        Khaled Omar_H_SL8_D2O
EXPNO                  10
PROCNO                  1

F2 - Acquisition Parameters
Date_           20200825
Time               12.36
INSTRUM            spect
PROBHD    5 mm PABBO BB/
PULPROG             zg30
TD                 65536
SOLVENT             DMSO
NS                    32
DS                     2
SWH             8012.820 Hz
FIDRES          0.122266 Hz
AQ             4.0894465 sec
RG                106.37
DW                62.400 usec
DE                  6.50 usec
TE                 298.0 K
D1            1.00000000 sec
TD0                    1

======== CHANNEL f1 ========
SFO1         400.1924713 MHz
NUC1                  1H
P1                 15.00 usec
PLW1         10.39999962 W

F2 - Processing parameters
SI                 65536
SF           400.1900000 MHz
WDW                   EM
SSB      0
LB                  0.30 Hz
GB       0
PC                  1.00
```

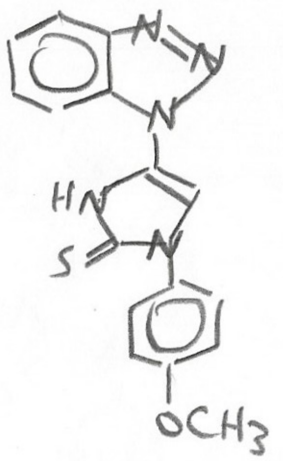

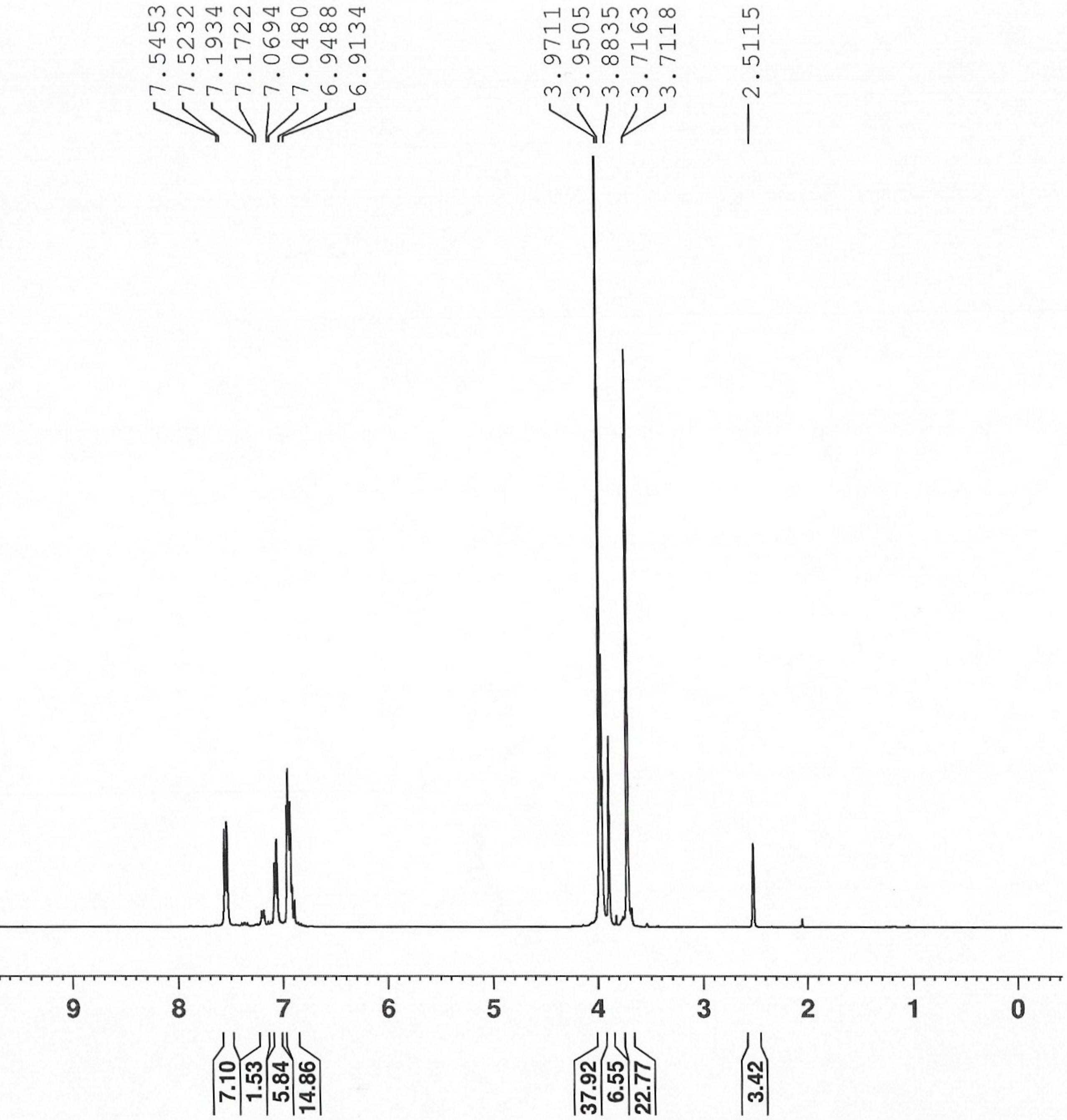

Khaled Omar_C_SL8

Microanalytical Unit - FOPCU - NMR laboratory
www.pharma.cu.edu.eg dir-mau.fopcu@pharma.cu.edu.eg

BI 4

^{13}C NMR

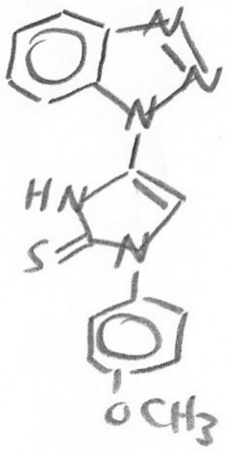


```
Current Data Parameters
NAME        Khaled Omar_C_SL8
EXPNO                      10
PROCNO                      1

F2 - Acquisition Parameters
Date_                20200824
Time                    20.07
INSTRUM                 spect
PROBHD      5 mm PABBO BB/
PULPROG               zgpg30
TD                      65536
SOLVENT                  DMSO
NS                       1200
DS                          4
SWH                 24038.461 Hz
FIDRES               0.366798 Hz
AQ                  1.3631488 sec
RG                     202.37
DW                     20.800 usec
DE                       6.50 usec
TE                      298.0 K
D1                 2.00000000 sec
D11                0.03000000 sec
TD0                         1

======== CHANNEL f1 ========
SFO1              100.6379178 MHz
NUC1                      13C
P1                      10.00 usec
PLW1              45.00000000 W

======== CHANNEL f2 ========
SFO2              400.1916008 MHz
NUC2                       1H
CPDPRG[2              waltz16
```

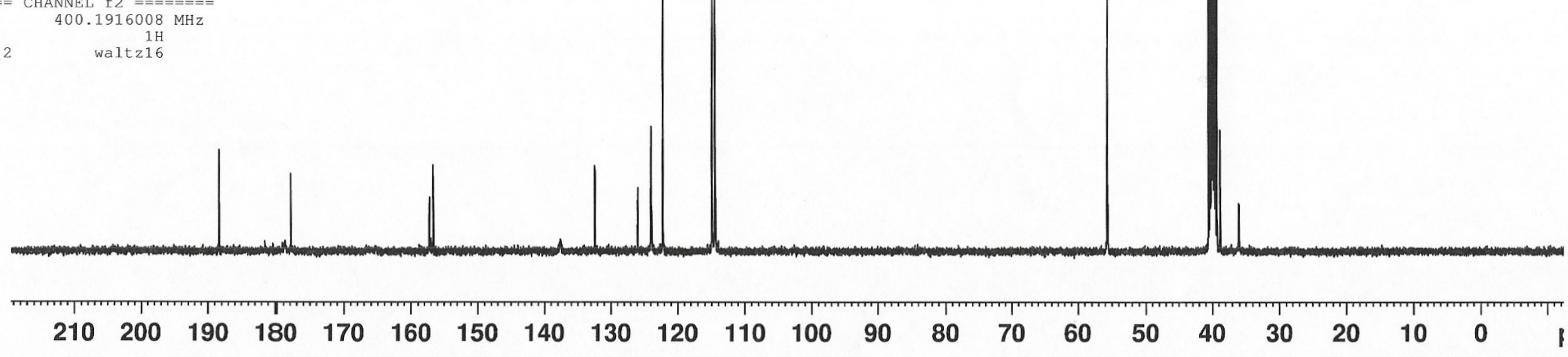

Khaled Omar_H_SL10 BI5

Microanalytical Unit - FOPCU - NMR laboratory
www.pharma.cu.edu.eg dir-mau.fopcu@pharma.cu.edu.eg

BRU[illegible]

```
Current Data Parameters
NAME      Khaled Omar_H_SL10
EXPNO                    10
PROCNO                    1

F2 - Acquisition Parameters
Date_              20200824
Time                  21.27
INSTRUM               spect
PROBHD    5 mm PABBO BB/
PULPROG                zg30
TD                    65536
SOLVENT                DMSO
NS                       32
DS                        2
SWH            8012.820 Hz
FIDRES         0.122266 Hz
AQ            4.0894465 sec
RG                   114.95
DW               62.400 usec
DE                 6.50 usec
TE                298.0 K
D1           1.00000000 sec
TD0                       1

======== CHANNEL f1 ========
SFO1        400.1924713 MHz
NUC1                     1H
P1                15.00 usec
PLW1        10.39999962 W

F2 - Processing parameters
SI                    65536
SF          400.1900000 MHz
WDW                      EM
SSB       0
LB                 0.30 Hz
GB        0
PC                     1.00
```

¹HNMR

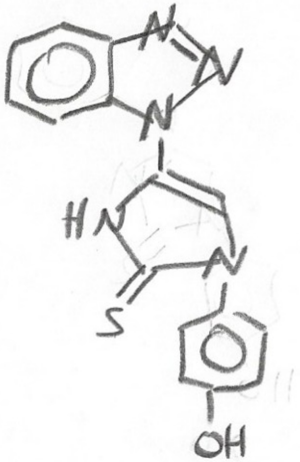

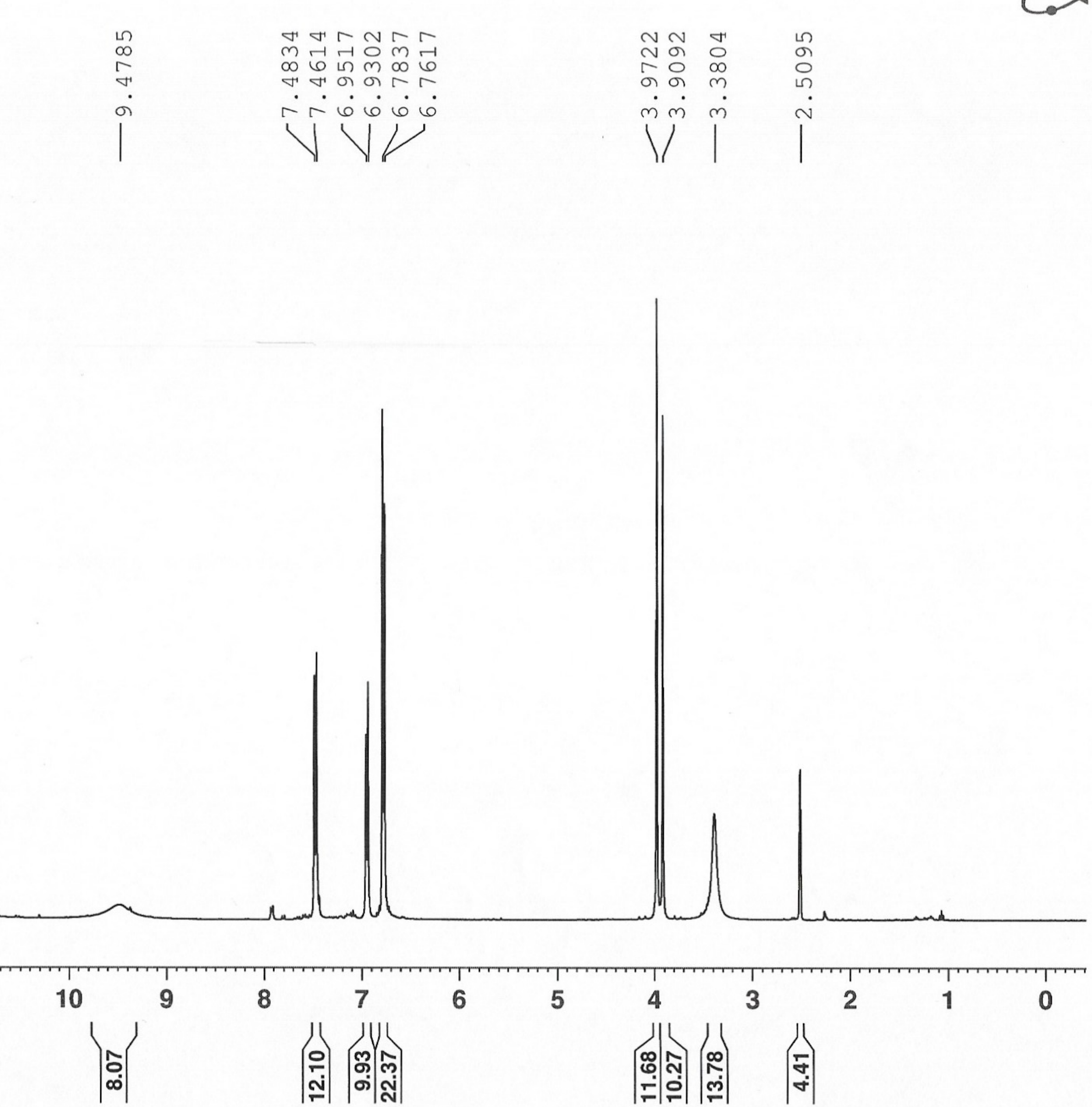

Khaled Omar_H_SL10_D2O

Microanalytical Unit - FOPCU - NMR laboratory
www.pharma.cu.edu.eg dir-mau.fopcu@pharma.cu.edu.eg

BI 5

^{1}HNMR+D2O

```
Current Data Parameters
NAME      Khaled Omar_H_SL10_D2O
EXPNO                    10
PROCNO                    1

F2 - Acquisition Parameters
Date_              20200825
Time                  12.46
INSTRUM               spect
PROBHD   5 mm PABBO BB/
PULPROG                zg30
TD                    65536
SOLVENT                DMSO
NS                       32
DS                        2
SWH                8012.820 Hz
FIDRES             0.122266 Hz
AQ                4.0894465 sec
RG                   114.95
DW                   62.400 usec
DE                     6.50 usec
TE                    298.0 K
D1              1.00000000 sec
TD0                       1

======== CHANNEL f1 ========
SFO1          400.1924713 MHz
NUC1                   1H
P1                  15.00 usec
PLW1          10.39999962 W

F2 - Processing parameters
SI                    65536
SF            400.1900000 MHz
WDW                      EM
SSB      0
LB                     0.30 Hz
GB       0
PC                     1.00
```

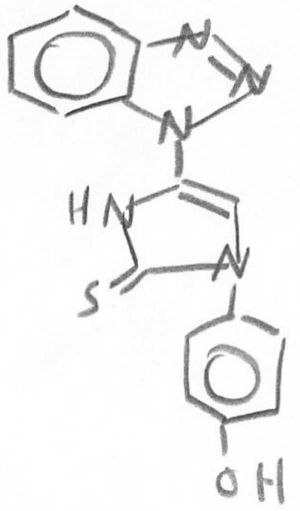

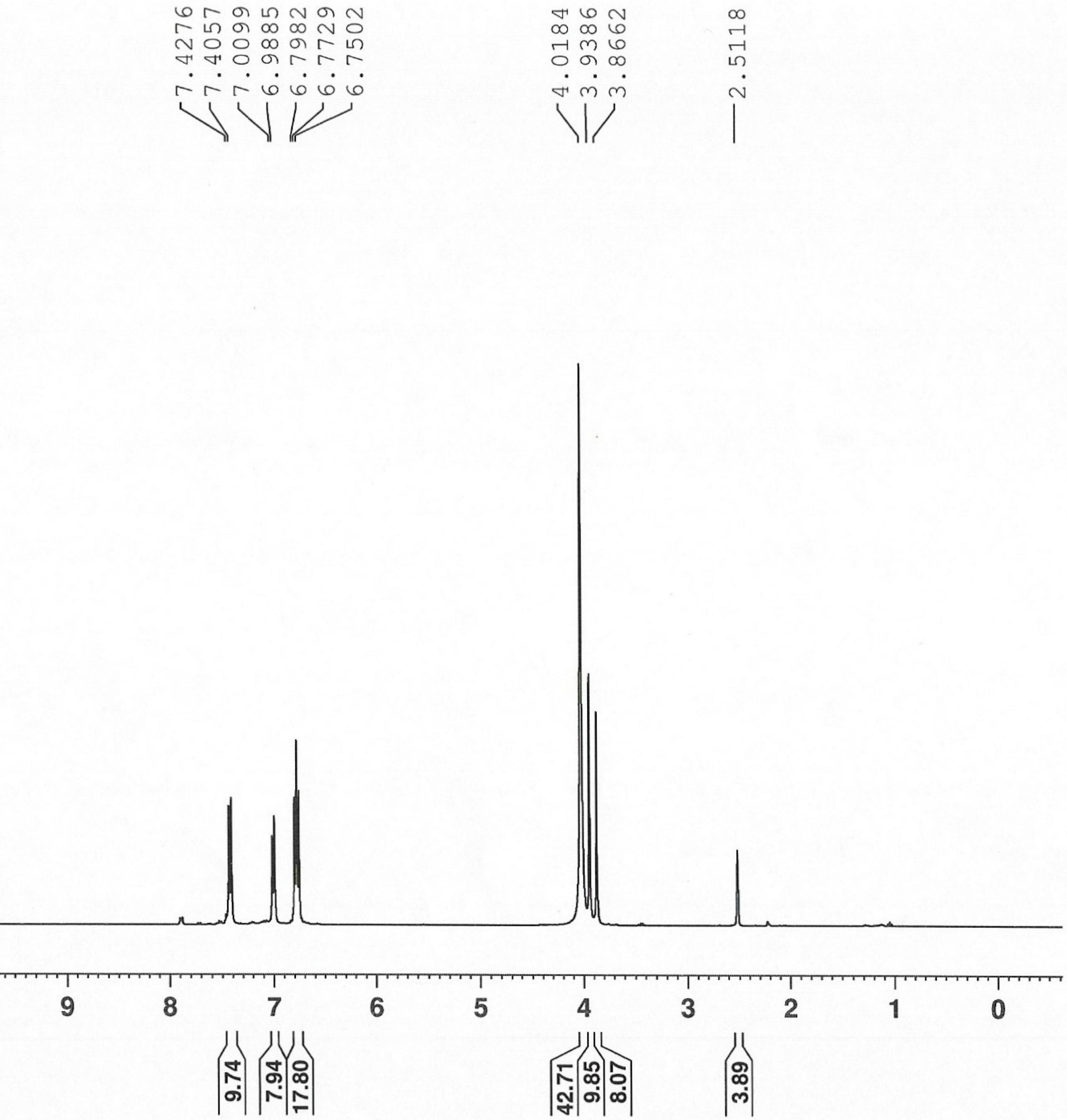

Khaled Omar_C_SL10

Microanalytical Unit - FOPCU - NMR laboratory
www.pharma.cu.edu.eg dir-mau.fopcu@pharma.cu.edu.eg

BI5

^{13}C NMR

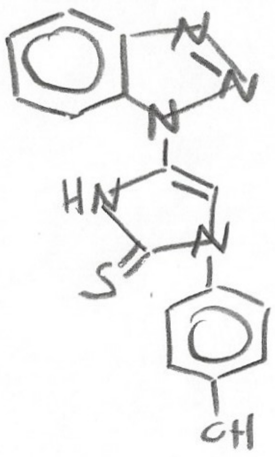

```
Current Data Parameters
NAME      Khaled Omar_C_SL10
EXPNO                   10
PROCNO                   1

F2 - Acquisition Parameters
Date_             20200824
Time                 22.36
INSTRUM              spect
PROBHD   5 mm PABBO BB/
PULPROG            zgpg30
TD                   65536
SOLVENT               DMSO
NS                    1200
DS                       4
SWH             24038.461 Hz
FIDRES           0.366798 Hz
AQ              1.3631488 sec
RG                  202.37
DW                  20.800 usec
DE                    6.50 usec
TE                   298.0 K
D1              2.00000000 sec
D11             0.03000000 sec
TD0                      1

======== CHANNEL f1 ========
SFO1          100.6379178 MHz
NUC1                  13C
P1                  10.00 usec
PLW1          45.00000000 W

======== CHANNEL f2 ========
SFO2          400.1916008 MHz
NUC2                   1H
CPDPRG[2           waltz16
```

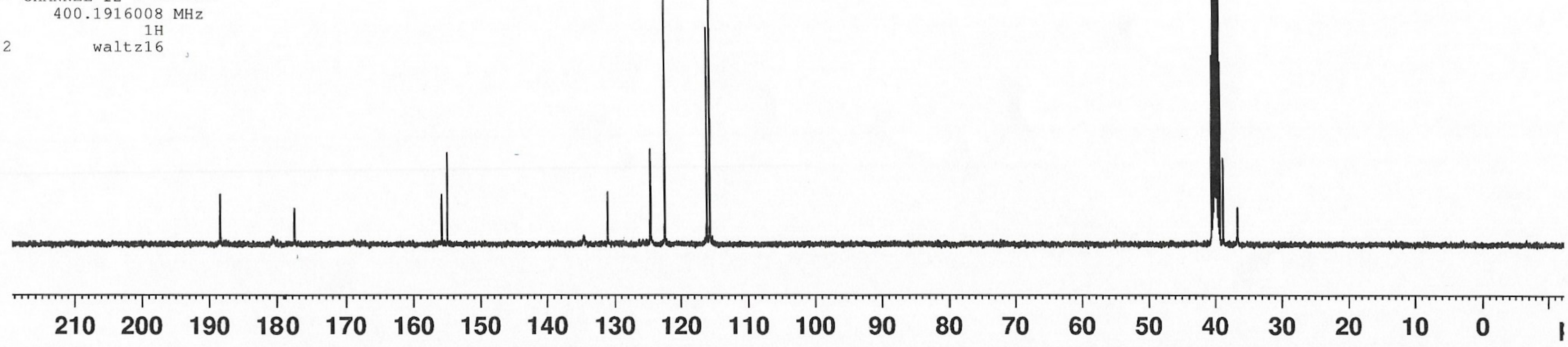

Khaled Omar_H_SL5 BI 6

Microanalytical Unit - FOPCU - NMR laboratory
www.pharma.cu.edu.eg dir-mau.fopcu@pharma.cu.edu.eg

BRUI

```
Current Data Parameters
NAME      Khaled Omar_H_SL5
EXPNO                  10
PROCNO                  1

F2 - Acquisition Parameters
Date_            20200824
Time                15.12
INSTRUM             spect
PROBHD   5 mm PABBO BB/
PULPROG              zg30
TD                  65536
SOLVENT              DMSO
NS                     32
DS                      2
SWH              8012.820 Hz
FIDRES           0.122266 Hz
AQ              4.0894465 sec
RG                 114.95
DW                 62.400 usec
DE                   6.50 usec
TE                  298.0 K
D1             1.00000000 sec
TD0                     1

======== CHANNEL f1 ========
SFO1          400.1924713 MHz
NUC1                   1H
P1                  15.00 usec
PLW1          10.39999962 W

F2 - Processing parameters
SI                  65536
SF            400.1900000 MHz
WDW                    EM
SSB      0
LB                   0.30 Hz
GB       0
PC                   1.00
```

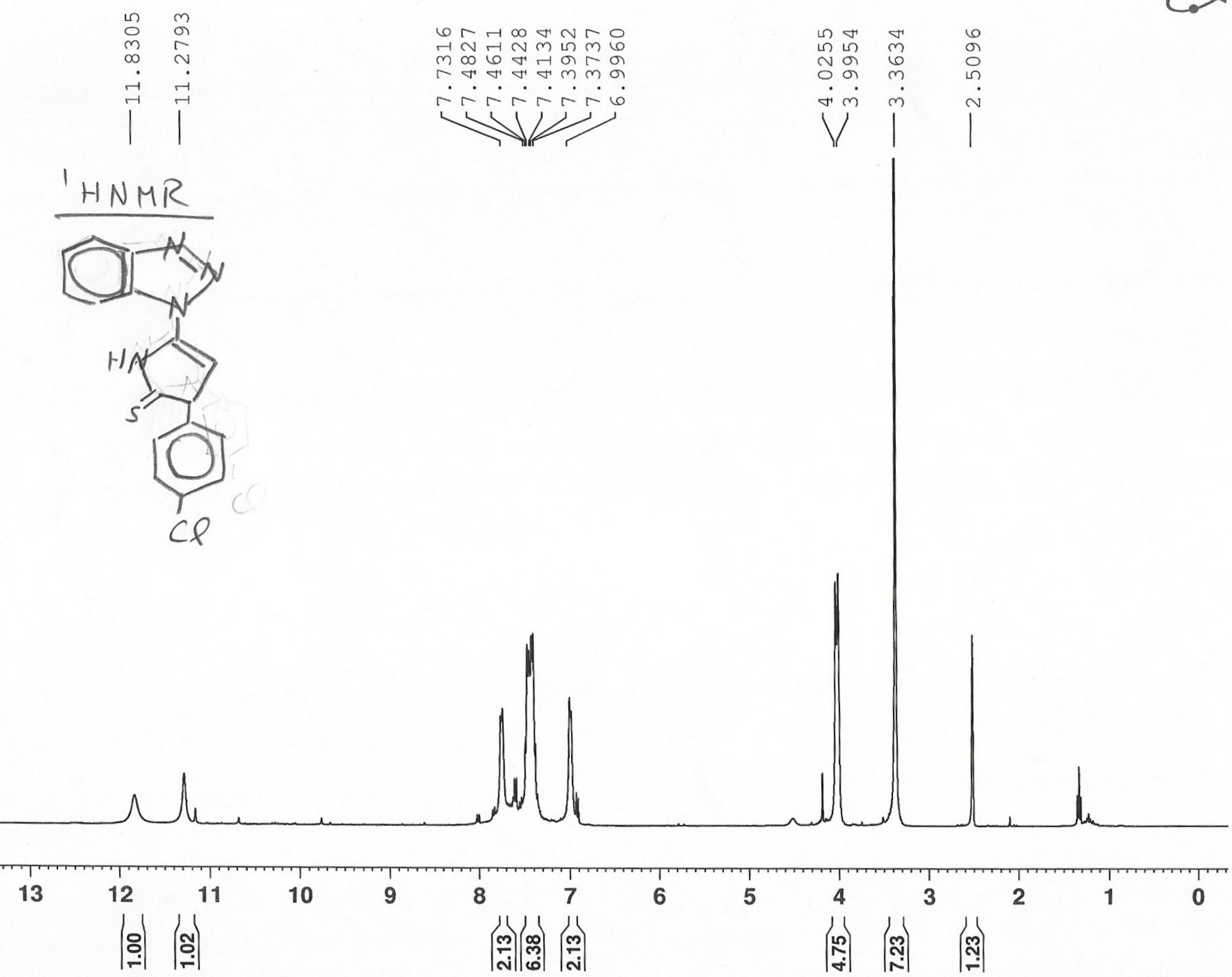

Khaled Omar_H_SL5_D2O

Microanalytical Unit - FOPCU - NMR laboratory
www.pharma.cu.edu.eg dir-mau.fopcu@pharma.cu.edu.eg

BI6

^{1}HNMR+D2O

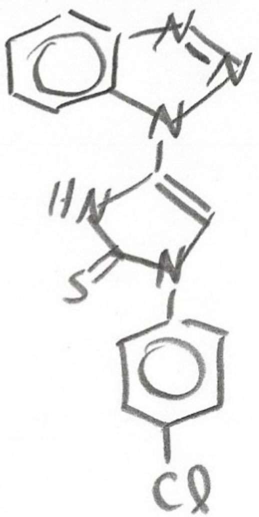


```
Current Data Parameters
NAME      Khaled Omar_H_SL5_D2O
EXPNO                10
PROCNO                1

F2 - Acquisition Parameters
Date_          20200825
Time              12.21
INSTRUM           spect
PROBHD   5 mm PABBO BB/
PULPROG            zg30
TD                65536
SOLVENT            DMSO
NS                   32
DS                    2
SWH            8012.820 Hz
FIDRES         0.122266 Hz
AQ            4.0894465 sec
RG               114.95
DW               62.400 usec
DE                 6.50 usec
TE                298.0 K
D1           1.00000000 sec
TD0                   1

======== CHANNEL f1 ========
SFO1        400.1924713 MHz
NUC1                 1H
P1                15.00 usec
PLW1        10.39999962 W

F2 - Processing parameters
SI                65536
SF          400.1900000 MHz
WDW                  EM
SSB       0
LB                 0.30 Hz
GB        0
PC                 1.00
```

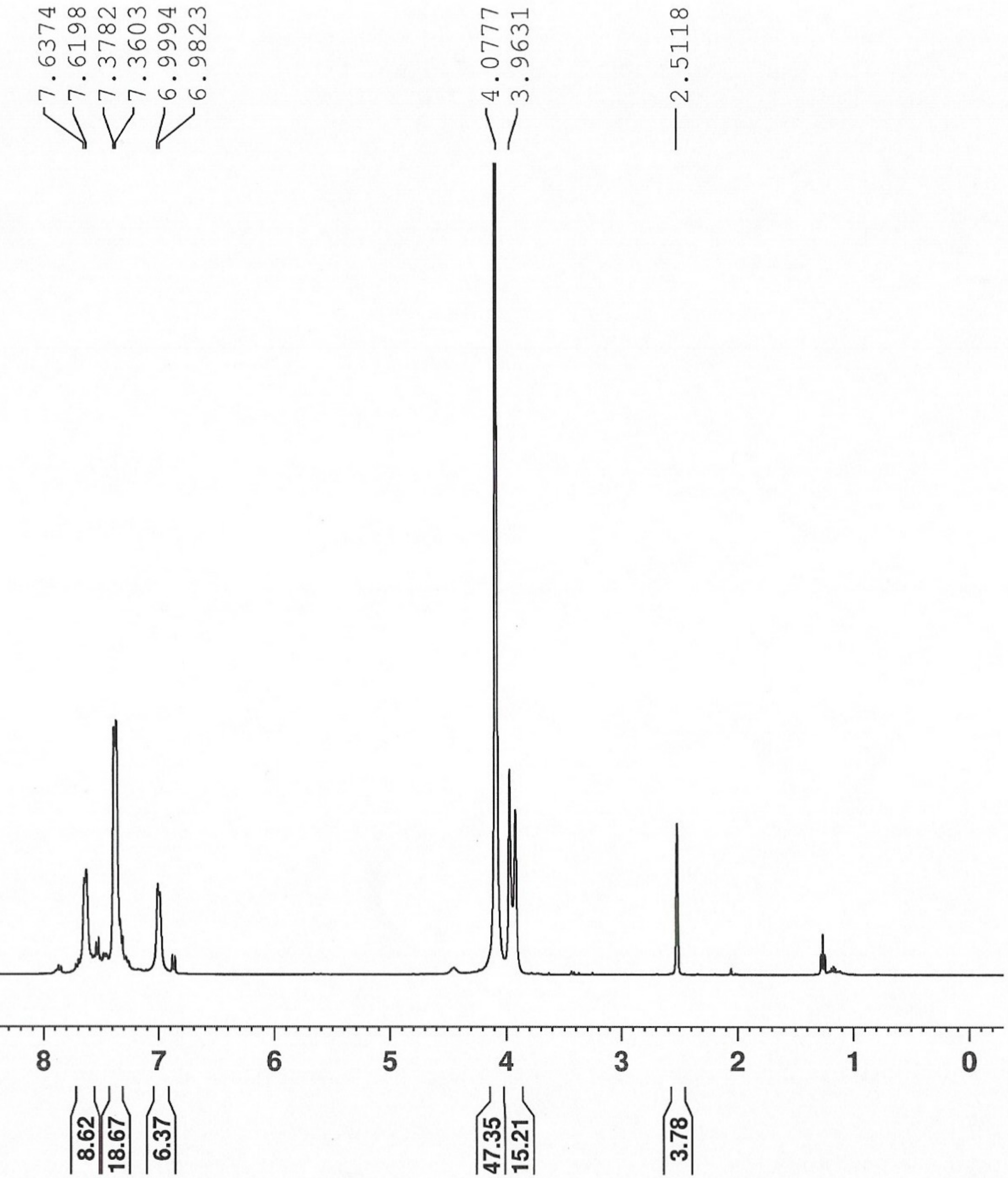

Khaled Omar_C_SL5

Microanalytical Unit - FOPCU - NMR laboratory
www.pharma.cu.edu.eg dir-mau.fopcu@pharma.cu.edu.eg

BRUKER

BI6

^{13}CNMR

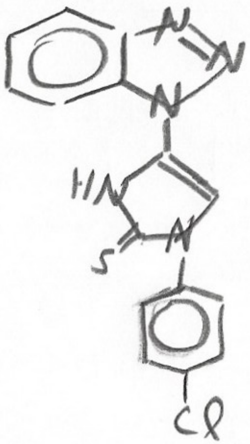

```
Current Data Parameters
AME      Khaled Omar_C_SL5
XPNO                    10
ROCNO                    1

2 - Acquisition Parameters
ate_              20200824
ime                  16.22
NSTRUM               spect
ROBHD    5 mm PABBO BB/
ULPROG              zgpg30
D                    65536
OLVENT                DMSO
S                     1200
S                        4
WH               24038.461 Hz
IDRES             0.366798 Hz
Q                1.3631488 sec
G                   202.37
W                   20.800 usec
E                     6.50 usec
E                    298.0 K
1               2.00000000 sec
11              0.03000000 sec
D0                       1

======== CHANNEL f1 ========
FO1         100.6379178 MHz
UC1                     13C
1                     10.00 usec
LW1             45.00000000 W

======== CHANNEL f2 ========
FO2         400.1916008 MHz
UC2                      1H
PDPRG[2             waltz16
```

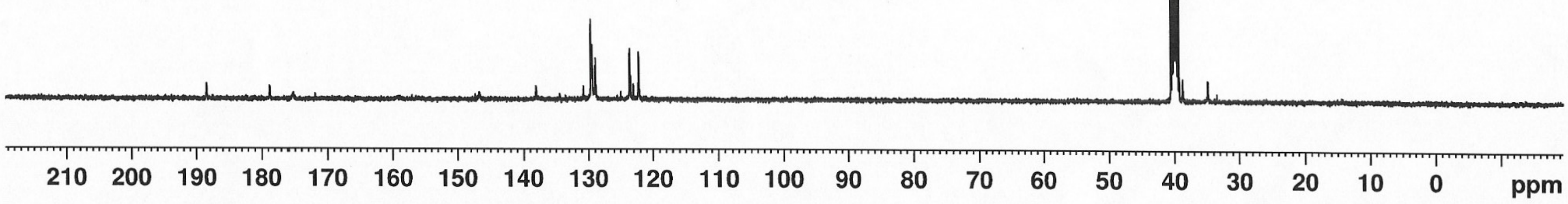

Khaled Omar_H_SL6 BI7

Microanalytical Unit - FOPCU - NMR laboratory
www.pharma.cu.edu.eg dir-mau.fopcu@pharma.cu.edu.eg

BRUKER

^{1}HNMR

Current Data Parameters
NAME Khaled Omar_H_SL6
EXPNO 10
PROCNO 1

F2 - Acquisition Parameters
Date_ 20200824
Time 16.27
INSTRUM spect
PROBHD 5 mm PABBO BB/
PULPROG zg30
TD 65536
SOLVENT DMSO
NS 32
DS 2
SWH 8012.820 Hz
FIDRES 0.122266 Hz
AQ 4.0894465 sec
RG 106.37
DW 62.400 usec
DE 6.50 usec
TE 298.0 K
D1 1.00000000 sec
TD0 1

======== CHANNEL f1 ========
SFO1 400.1924713 MHz
NUC1 1H
P1 15.00 usec
PLW1 10.39999962 W

F2 - Processing parameters
SI 65536
SF 400.1900000 MHz
WDW EM
SSB 0
LB 0.30 Hz
GB 0
PC 1.00

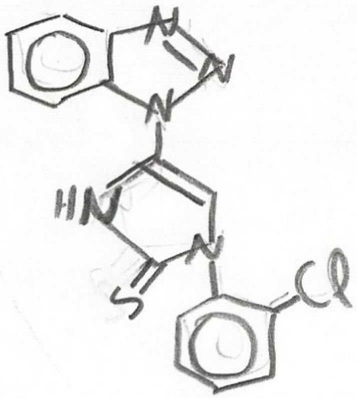

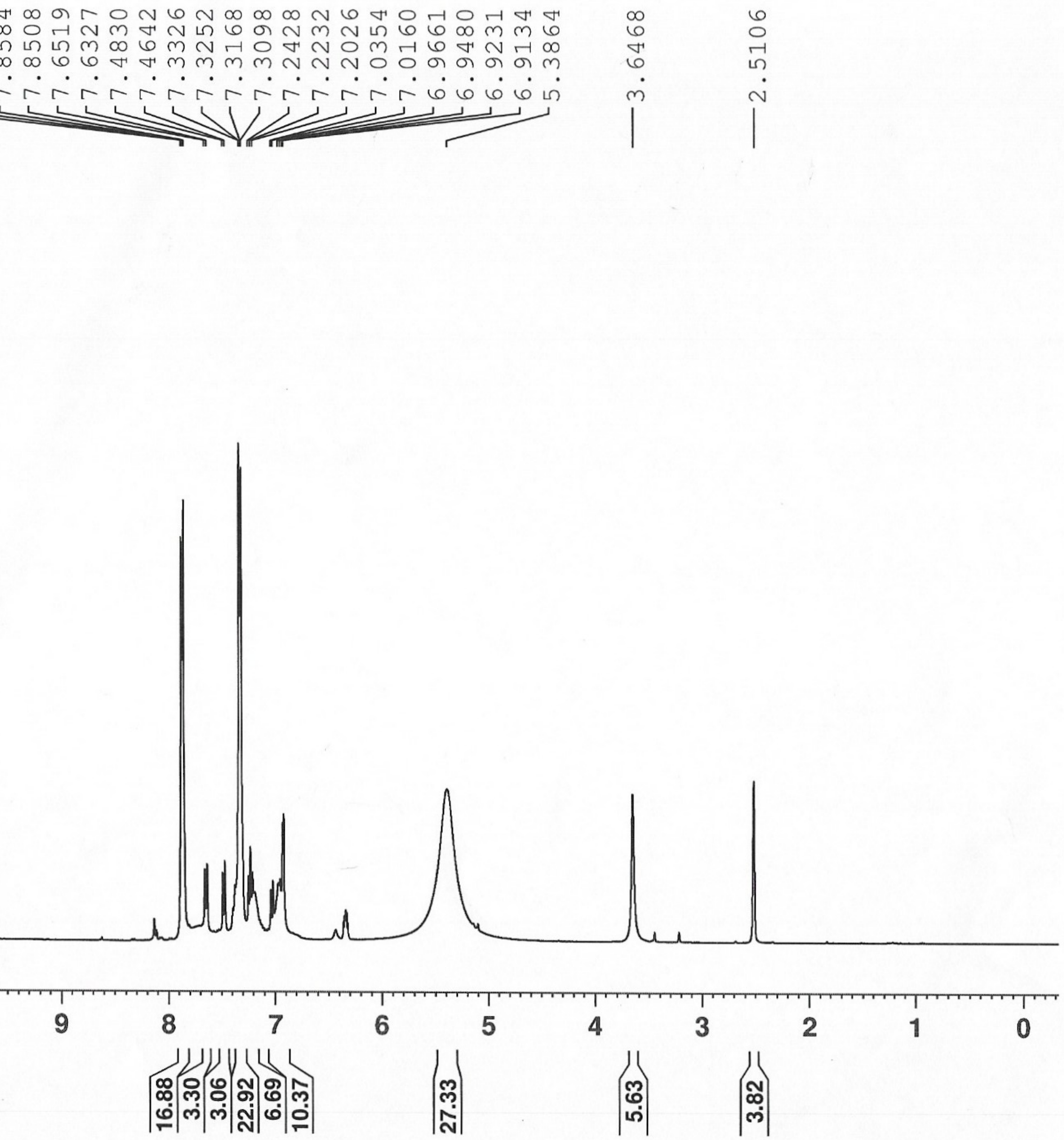

Khaled Omar_H_SL6_D2O

Microanalytical Unit - FOPCU - NMR laboratory
www.pharma.cu.edu.eg dir-mau.fopcu@pharma.cu.edu.eg

BI7
^{1}H NMR + D2O

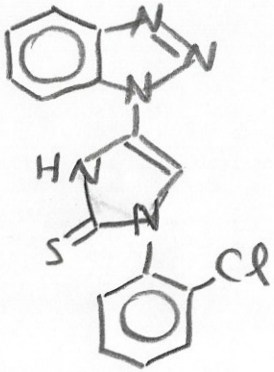


```
Current Data Parameters
NAME     Khaled Omar_H_SL6_D2O
EXPNO                 10
PROCNO                 1

F2 - Acquisition Parameters
Date_           20200825
Time               12.26
INSTRUM            spect
PROBHD   5 mm PABBO BB/
PULPROG             zg30
TD                 65536
SOLVENT             DMSO
NS                    32
DS                     2
SWH             8012.820 Hz
FIDRES          0.122266 Hz
AQ             4.0894465 sec
RG                106.37
DW                62.400 usec
DE                  6.50 usec
TE                 298.0 K
D1            1.00000000 sec
TD0                    1

======== CHANNEL f1 ========
SFO1         400.1924713 MHz
NUC1                  1H
P1                 15.00 usec
PLW1         10.39999962 W

F2 - Processing parameters
SI                 65536
SF           400.1900000 MHz
WDW                   EM
SSB      0
LB                  0.30 Hz
GB       0
PC                  1.00
```

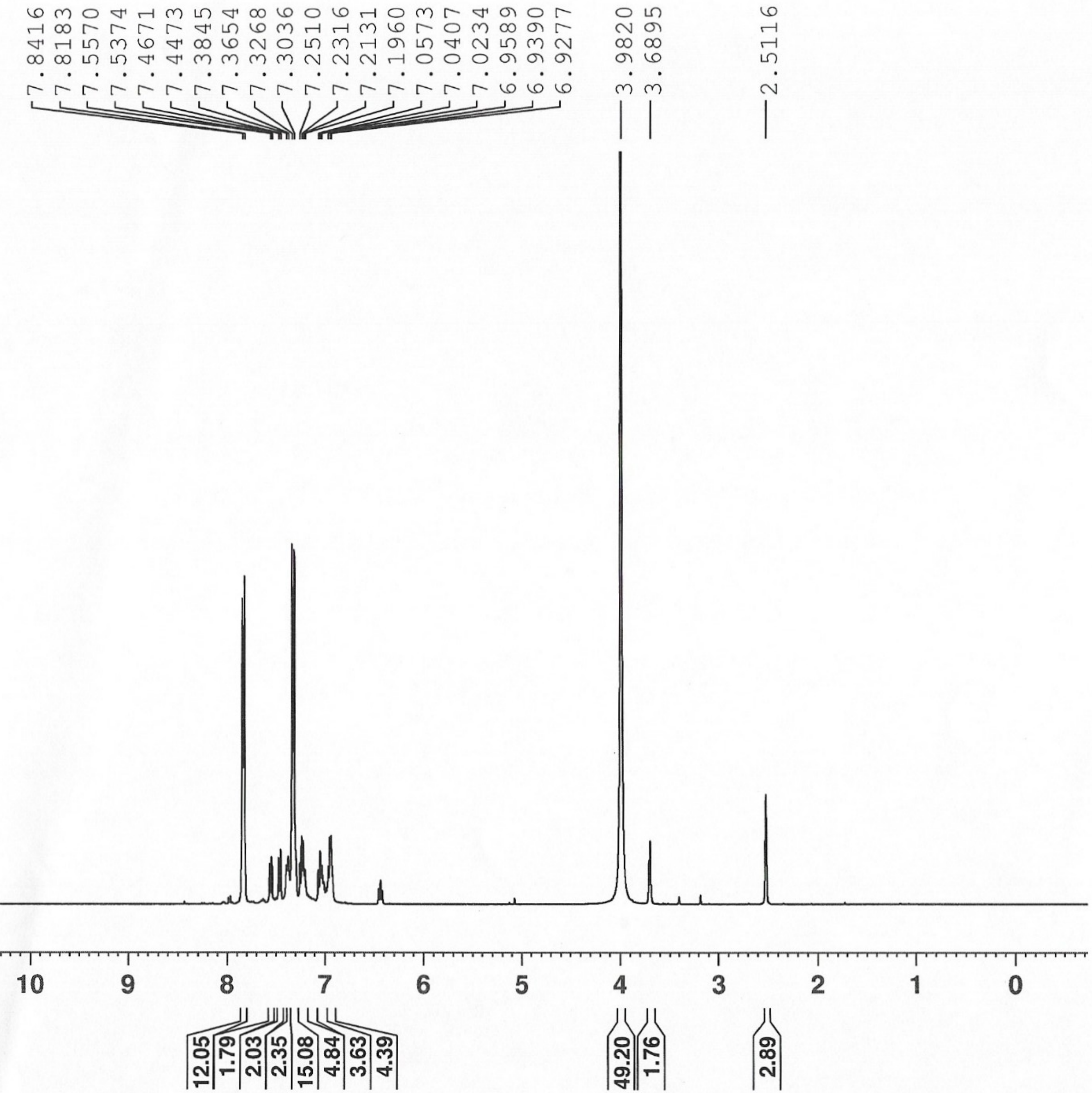

Khaled Omar_C_SL6

Microanalytical Unit - FOPCU - NMR laboratory
www.pharma.cu.edu.eg dir-mau.fopcu@pharma.cu.edu.eg

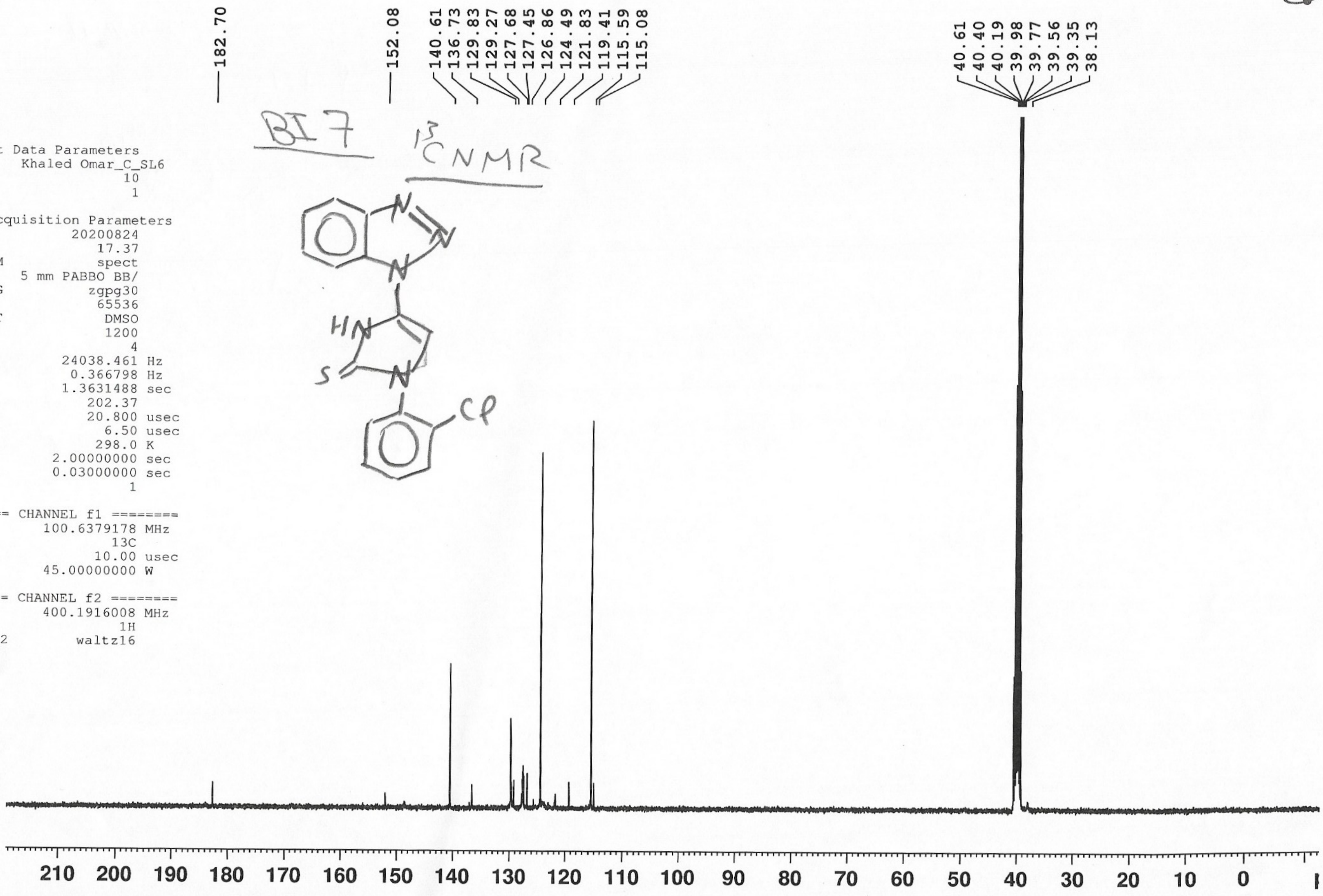

BI7 ^{13}C NMR

```
Current Data Parameters
NAME        Khaled Omar_C_SL6
EXPNO                      10
PROCNO                      1

F2 - Acquisition Parameters
Date_               20200824
Time                   17.37
INSTRUM                spect
PROBHD     5 mm PABBO BB/
PULPROG              zgpg30
TD                    65536
SOLVENT                DMSO
NS                     1200
DS                        4
SWH              24038.461 Hz
FIDRES            0.366798 Hz
AQ               1.3631488 sec
RG                   202.37
DW                   20.800 usec
DE                     6.50 usec
TE                    298.0 K
D1              2.00000000 sec
D11             0.03000000 sec
TD0                       1

======== CHANNEL f1 ========
SFO1          100.6379178 MHz
NUC1                  13C
P1                  10.00 usec
PLW1          45.00000000 W

======== CHANNEL f2 ========
SFO2          400.1916008 MHz
NUC2                   1H
CPDPRG[2           waltz16
```

Khaled Omar_H_SL4 BI8

¹H NMR

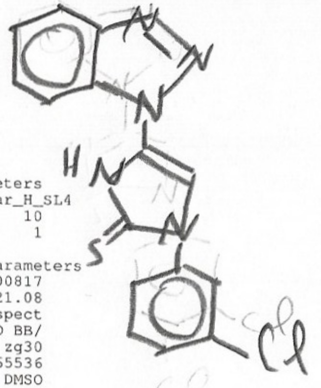

Microanalytical Unit - FOPCU - NMR laboratory
www.pharma.cu.edu.eg dir-mau.fopcu@pharma.cu.edu.eg

BRUKER

```
Current Data Parameters
NAME      Khaled Omar_H_SL4
EXPNO                    10
PROCNO                    1

F2 - Acquisition Parameters
Date_             20200817
Time                 21.08
INSTRUM              spect
PROBHD   5 mm PABBO BB/
PULPROG               zg30
TD                   65536
SOLVENT               DMSO
NS                      32
DS                       2
SWH               8012.820 Hz
FIDRES            0.122266 Hz
AQ               4.0894465 sec
RG                  129.43
DW                  62.400 usec
DE                    6.50 usec
TE                   298.0 K
D1              1.00000000 sec
TD0                      1

======== CHANNEL f1 ========
SFO1           400.1924713 MHz
NUC1                    1H
P1                   15.00 usec
PLW1           10.39999962 W

F2 - Processing parameters
SI                   65536
SF             400.1900000 MHz
WDW                     EM
SSB      0
LB                    0.30 Hz
GB       0
PC                    1.00
```

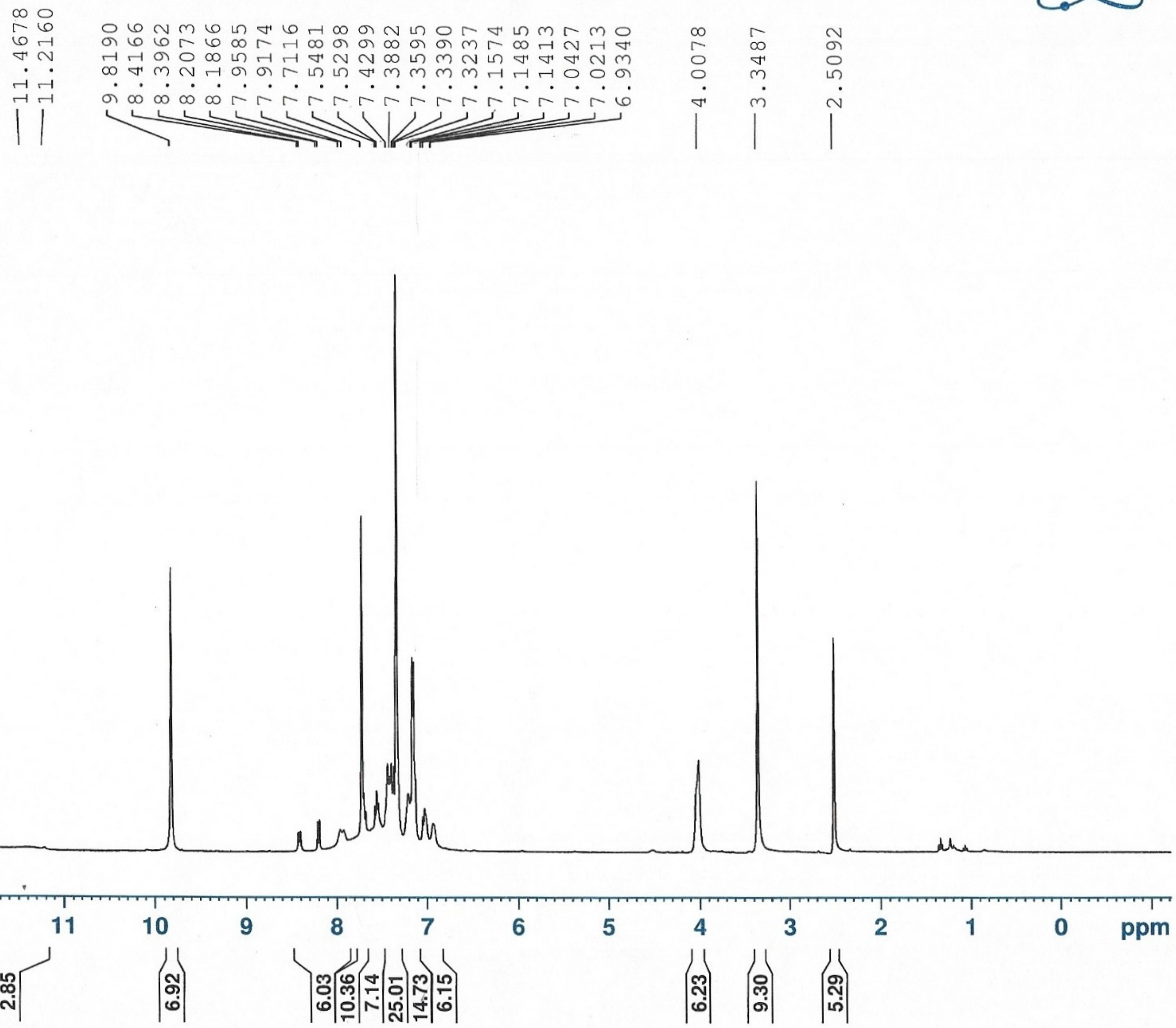

Khaled Omar_H_SL4_D2O BI 8

Microanalytical Unit - FOPCU - NMR laboratory
www.pharma.cu.edu.eg dir-mau.fopcu@pharma.cu.edu.eg

BRUKER

^1H NMR + D_2O

```
Current Data Parameters
NAME       Khaled Omar_H_SL4_D2O
EXPNO                      10
PROCNO                      1

F2 - Acquisition Parameters
Date_              20200818
Time                  11.17
INSTRUM               spect
PROBHD   5 mm PABBO BB/
PULPROG                zg30
TD                    65536
SOLVENT                DMSO
NS                       32
DS                        2
SWH               8012.820 Hz
FIDRES            0.122266 Hz
AQ               4.0894465 sec
RG                   129.43
DW                   62.400 usec
DE                     6.50 usec
TE                    298.0 K
D1              1.00000000 sec
TD0                       1

======== CHANNEL f1 ========
SFO1           400.1924713 MHz
NUC1                    1H
P1                   15.00 usec
PLW1           10.39999962 W

F2 - Processing parameters
SI                    65536
SF             400.1900000 MHz
WDW                      EM
SSB      0
LB                     0.30 Hz
GB       0
PC                     1.00
```

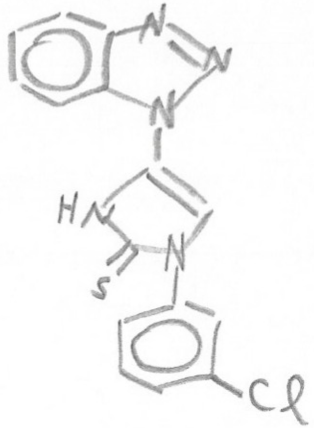

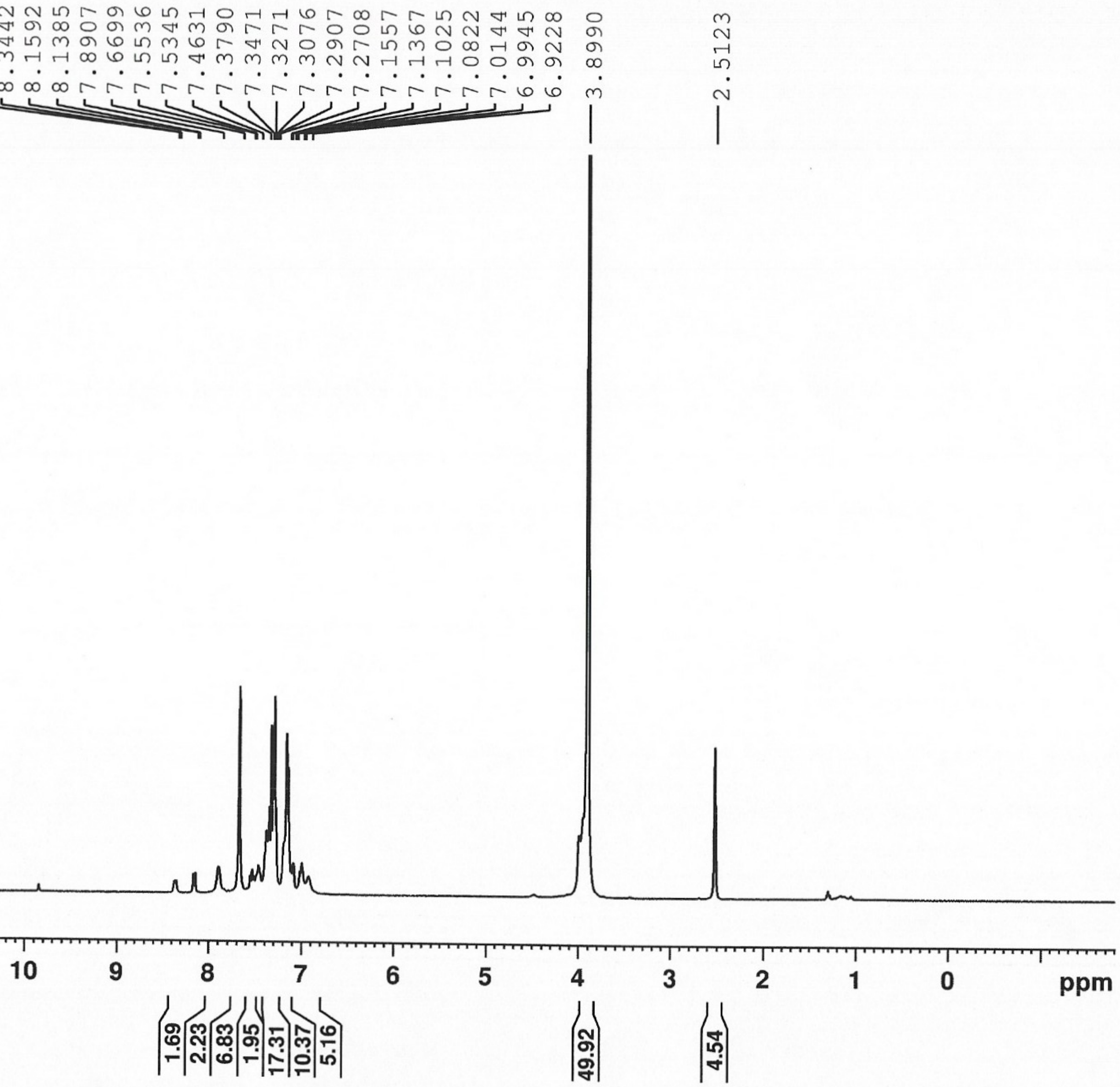

Khaled Omar_C_SL4

Microanalytical Unit - FOPCU - NMR laboratory
www.pharma.cu.edu.eg dir-mau.fopcu@pharma.cu.edu.eg

BRUKER

BI 8

^{13}C NMR

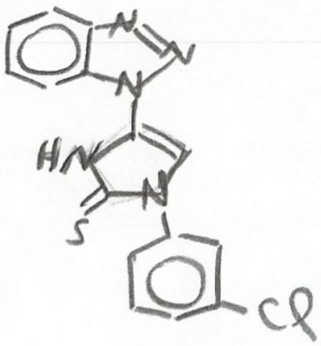

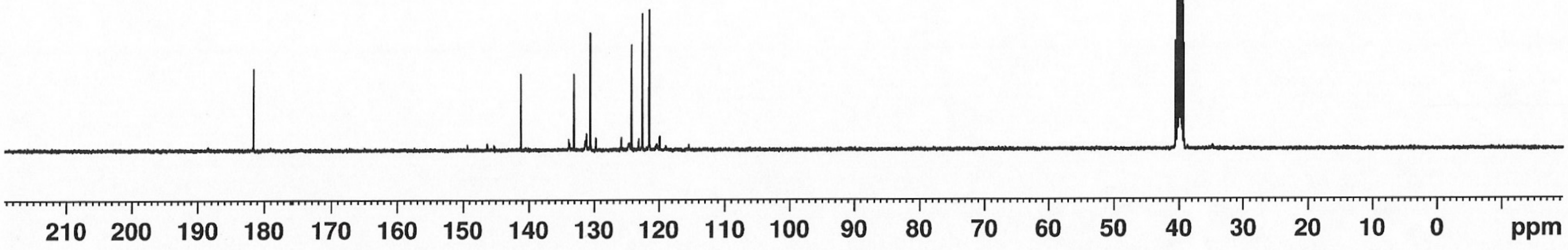


```
Current Data Parameters
NAME      Khaled Omar_C_SL4
EXPNO                   10
PROCNO                   1

F2 - Acquisition Parameters
Date_            20200817
Time                22.35
INSTRUM             spect
PROBHD   5 mm PABBO BB/
PULPROG            zgpg30
TD                  65536
SOLVENT              DMSO
NS                   1500
DS                      4
SWH             24038.461 Hz
FIDRES           0.366798 Hz
AQ              1.3631488 sec
RG                 202.37
DW                 20.800 usec
DE                   6.50 usec
TE                  298.0 K
D1             2.00000000 sec
D11            0.03000000 sec
TD0                     1

======== CHANNEL f1 ========
SFO1         100.6379178 MHz
NUC1                 13C
P1                 10.00 usec
PLW1         45.00000000 W

======== CHANNEL f2 ========
SFO2         400.1916008 MHz
NUC2                  1H
CPDPRG[2          waltz16
```

Khaled Omar_H_SL12 BI9

¹HNMR

Microanalytical Unit - FOPCU - NMR laboratory
www.pharma.cu.edu.eg dir-mau.fopcu@pharma.cu.edu.eg

BRU

```
Current Data Parameters
NAME     Khaled Omar_H_SL12
EXPNO                   10
PROCNO                   1

F2 - Acquisition Parameters
Date_           20200824
Time               23.56
INSTRUM            spect
PROBHD   5 mm PABBO BB/
PULPROG             zg30
TD                 65536
SOLVENT             DMSO
NS                    32
DS                     2
SWH             8012.820 Hz
FIDRES          0.122266 Hz
AQ             4.0894465 sec
RG                106.37
DW                62.400 usec
DE                  6.50 usec
TE                 298.0 K
D1            1.00000000 sec
TD0                    1

======== CHANNEL f1 ========
SFO1         400.1924713 MHz
NUC1                  1H
P1                 15.00 usec
PLW1         10.39999962 W

F2 - Processing parameters
SI                 65536
SF           400.1900000 MHz
WDW                   EM
SSB      0
LB                  0.30 Hz
GB       0
PC                  1.00
```

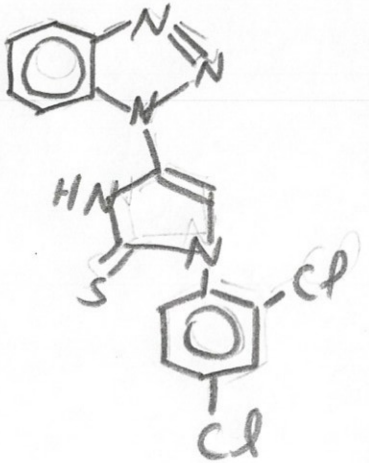

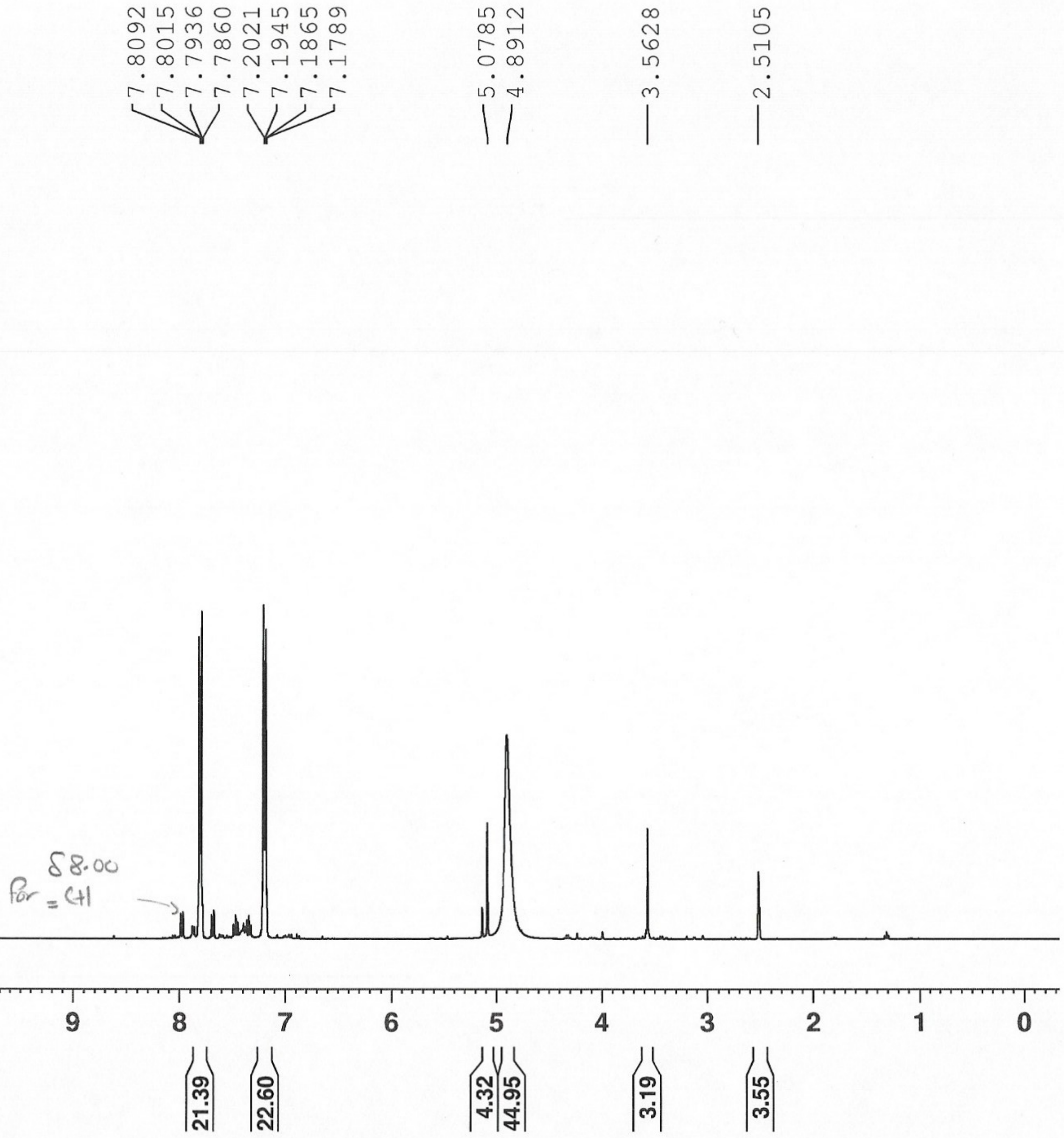

Khaled Omar_H_SL12_D2O

Microanalytical Unit - FOPCU - NMR laboratory
www.pharma.cu.edu.eg dir-mau.fopcu@pharma.cu.edu.eg

BI 9

^{1}HNMR + D_2O

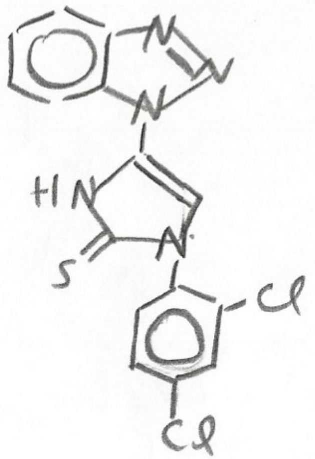


```
Current Data Parameters
NAME       Khaled Omar_H_SL12_D2O
EXPNO                        10
PROCNO                        1

F2 - Acquisition Parameters
Date_              20200825
Time                  12.58
INSTRUM               spect
PROBHD   5 mm PABBO BB/
PULPROG                zg30
TD                    65536
SOLVENT                DMSO
NS                       32
DS                        2
SWH                8012.820 Hz
FIDRES             0.122266 Hz
AQ                4.0894465 sec
RG                   106.37
DW                   62.400 usec
DE                     6.50 usec
TE                    298.0 K
D1               1.00000000 sec
TD0                       1

======== CHANNEL f1 ========
SFO1          400.1924713 MHz
NUC1                   1H
P1                  15.00 usec
PLW1          10.39999962 W

F2 - Processing parameters
SI                    65536
SF            400.1900000 MHz
WDW                      EM
SSB       0
LB                     0.30 Hz
GB        0
PC                     1.00
```

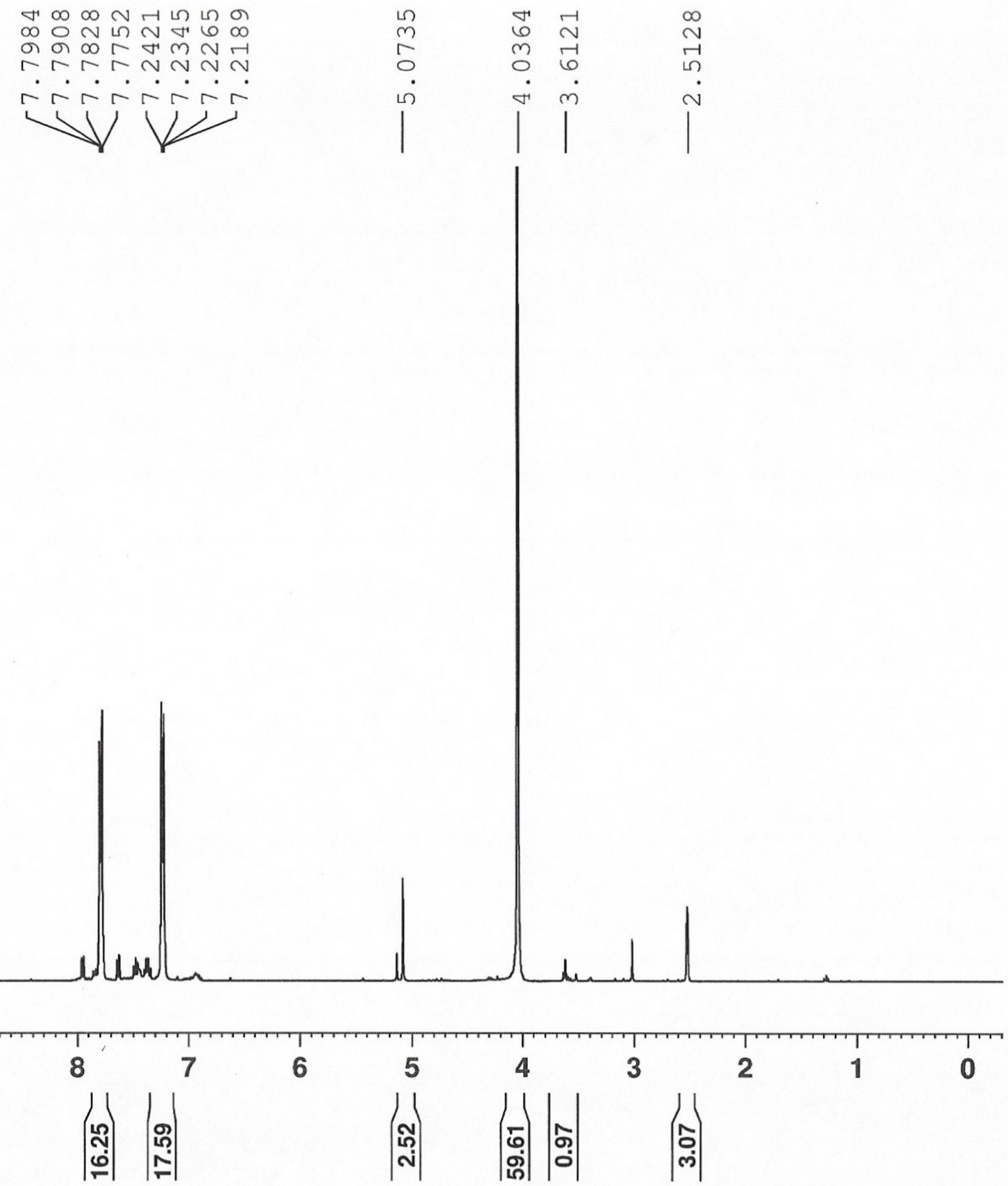

Khaled Omar_C_SL12

Microanalytical Unit - FOPCU - NMR laboratory
www.pharma.cu.edu.eg dir-mau.fopcu@pharma.cu.edu.eg

```
Current Data Parameters
NAME       Khaled Omar_C_SL12
EXPNO                    10
PROCNO                    1

F2 - Acquisition Parameters
Date_              20200825
Time                   1.06
INSTRUM               spect
PROBHD    5 mm PABBO BB/
PULPROG              zgpg30
TD                    65536
SOLVENT                DMSO
NS                     1200
DS                        4
SWH               24038.461 Hz
FIDRES             0.366798 Hz
AQ                1.3631488 sec
RG                   202.37
DW                   20.800 usec
DE                     6.50 usec
TE                    298.0 K
D1               2.00000000 sec
D11              0.03000000 sec
TD0                       1

======== CHANNEL f1 ========
SFO1         100.6379178 MHz
NUC1                     13C
P1                     10.00 usec
PLW1             45.00000000 W

======== CHANNEL f2 ========
SFO2         400.1916008 MHz
NUC2                      1H
CPDPRG[2             waltz16
```

BI 9
^{13}CNMR

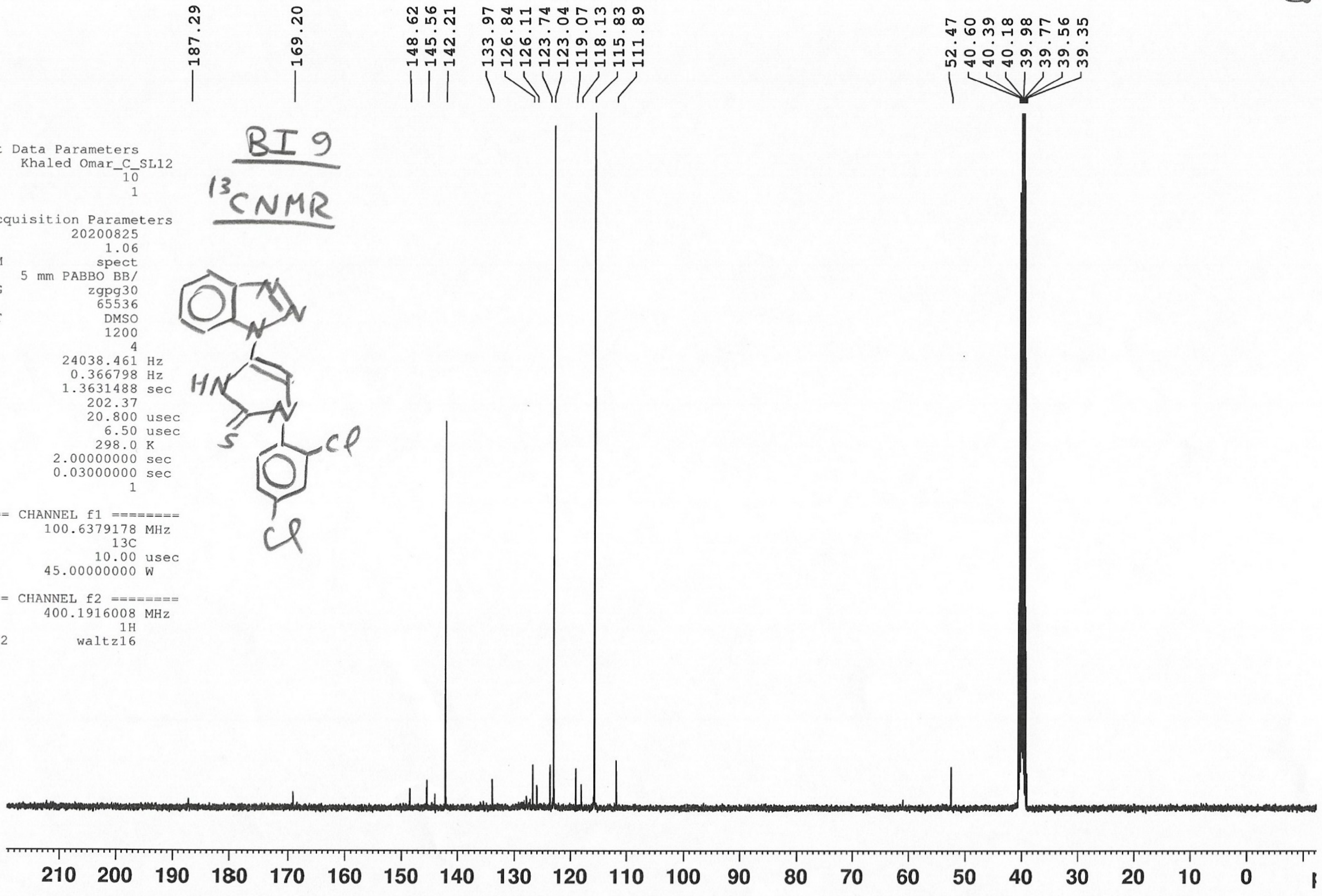

Khaled Omar_H_SL7

BI 10

1H NMR

Microanalytical Unit - FOPCU - NMR laboratory
www.pharma.cu.edu.eg dir-mau.fopcu@pharma.cu.edu.eg

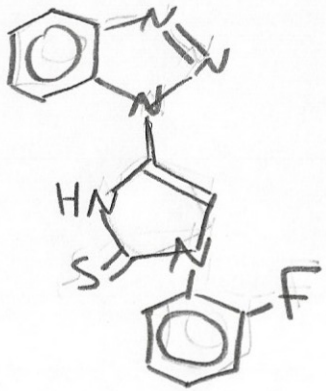


```
Current Data Parameters
NAME      Khaled Omar_H_SL7
EXPNO                  10
PROCNO                  1

F2 - Acquisition Parameters
Date_            20200824
Time                17.42
INSTRUM             spect
PROBHD   5 mm PABBO BB/
PULPROG              zg30
TD                  65536
SOLVENT              DMSO
NS                     32
DS                      2
SWH              8012.820 Hz
FIDRES           0.122266 Hz
AQ              4.0894465 sec
RG                 114.95
DW                 62.400 usec
DE                   6.50 usec
TE                  298.0 K
D1             1.00000000 sec
TD0                     1

======== CHANNEL f1 ========
SFO1          400.1924713 MHz
NUC1                   1H
P1                  15.00 usec
PLW1          10.39999962 W

F2 - Processing parameters
SI                  65536
SF            400.1900000 MHz
WDW                    EM
SSB      0
LB                   0.30 Hz
GB       0
PC                   1.00
```

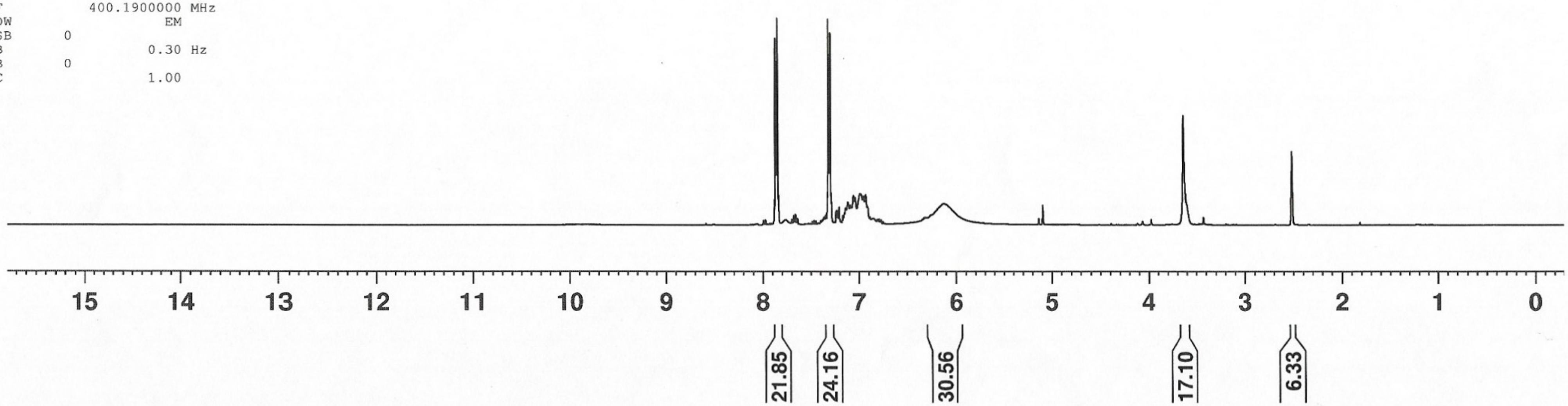

Khaled Omar_H_SL7_D2O

Microanalytical Unit - FOPCU - NMR laboratory
www.pharma.cu.edu.eg dir-mau.fopcu@pharma.cu.edu.eg

BRUKER

BI 10

$^1HNMR + D_2O$

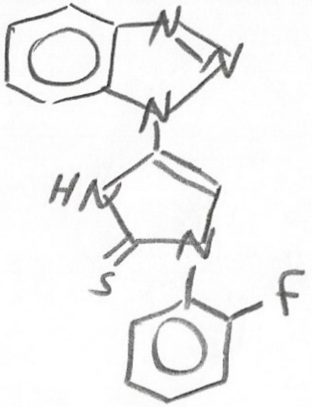


```
Current Data Parameters
NAME      Khaled Omar_H_SL7_D2O
EXPNO                   10
PROCNO                   1

F2 - Acquisition Parameters
Date_             20200825
Time                 12.31
INSTRUM              spect
PROBHD   5 mm PABBO BB/
PULPROG               zg30
TD                   65536
SOLVENT               DMSO
NS                      32
DS                       2
SWH               8012.820 Hz
FIDRES            0.122266 Hz
AQ               4.0894465 sec
RG                  114.95
DW                  62.400 usec
DE                    6.50 usec
TE                   298.0 K
D1              1.00000000 sec
TD0                      1

======== CHANNEL f1 ========
SFO1         400.1924713 MHz
NUC1                   1H
P1                   15.00 usec
PLW1          10.39999962 W

F2 - Processing parameters
SI                   65536
SF             400.1900000 MHz
WDW                     EM
SSB      0
LB                    0.30 Hz
GB       0
PC                    1.00
```

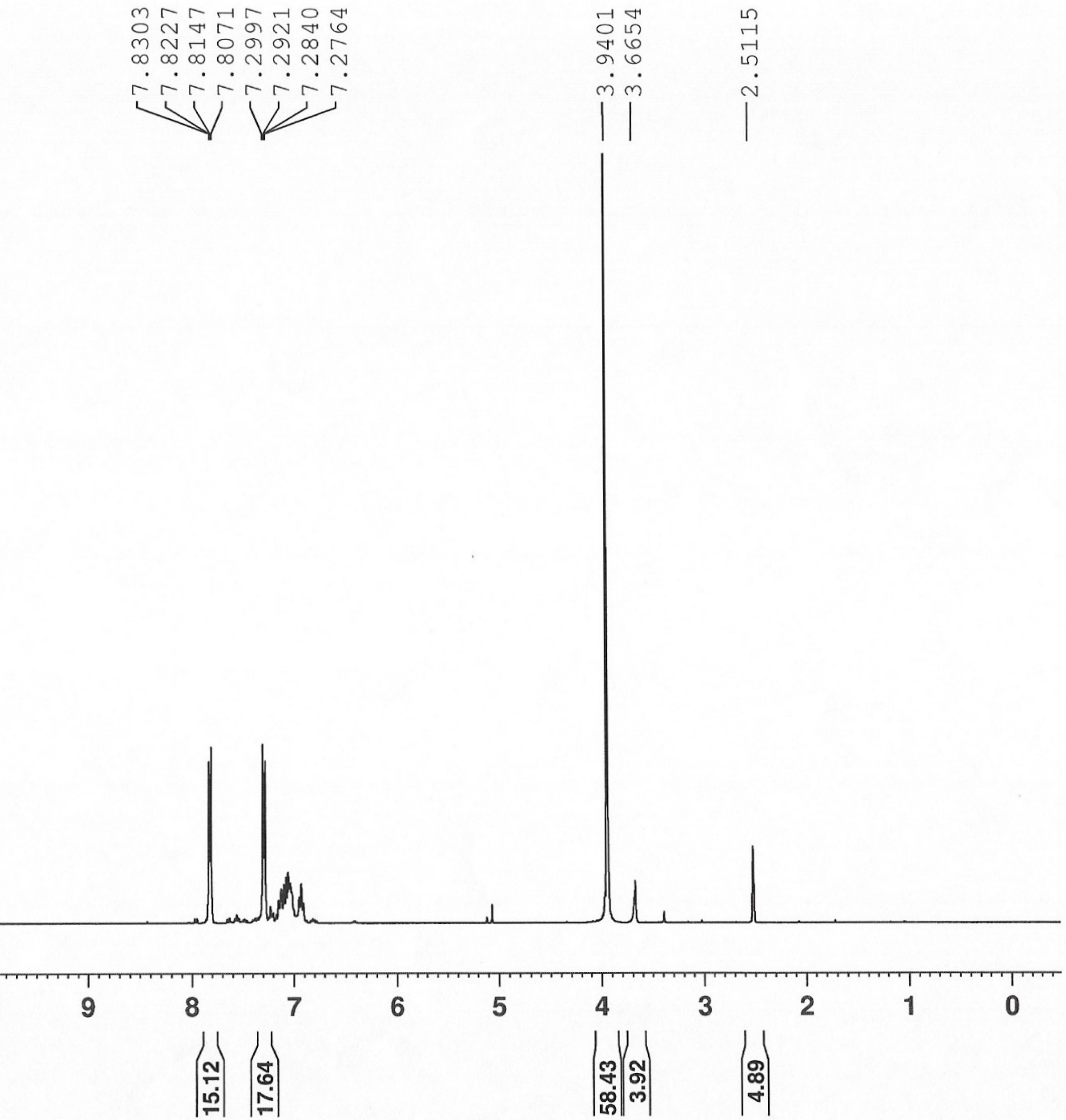

Khaled Omar_C_SL7

BI 10 13C NMR

Microanalytical Unit - FOPCU - NMR laboratory
www.pharma.cu.edu.eg dir-mau.fopcu@pharma.cu.edu.eg

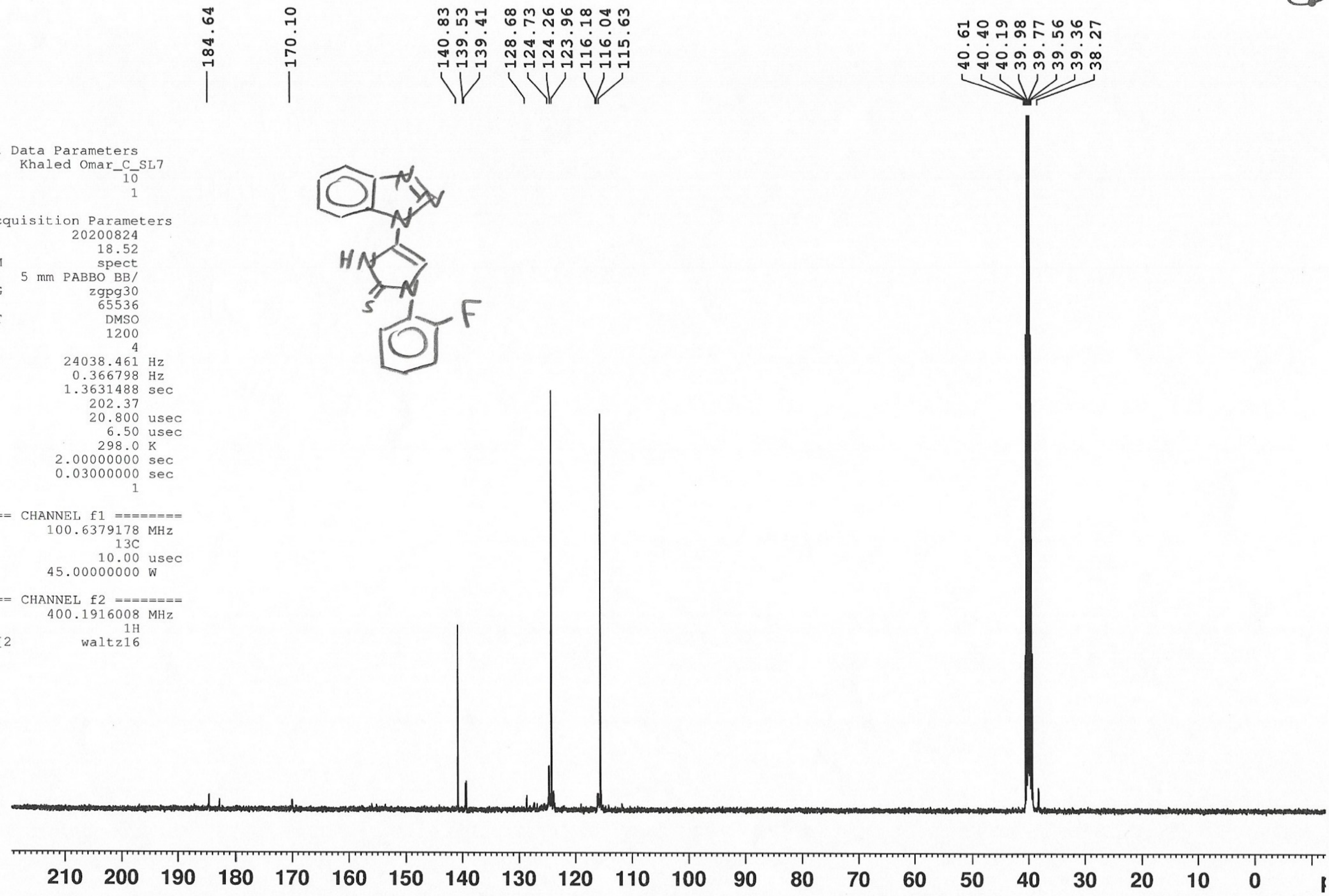


```
Current Data Parameters
NAME     Khaled Omar_C_SL7
EXPNO                  10
PROCNO                  1

F2 - Acquisition Parameters
Date_            20200824
Time                18.52
INSTRUM             spect
PROBHD   5 mm PABBO BB/
PULPROG           zgpg30
TD                 65536
SOLVENT             DMSO
NS                  1200
DS                     4
SWH            24038.461 Hz
FIDRES          0.366798 Hz
AQ             1.3631488 sec
RG                202.37
DW                20.800 usec
DE                  6.50 usec
TE                 298.0 K
D1            2.00000000 sec
D11           0.03000000 sec
TD0                    1

======== CHANNEL f1 ========
SFO1         100.6379178 MHz
NUC1                 13C
P1                 10.00 usec
PLW1         45.00000000 W

======== CHANNEL f2 ========
SFO2         400.1916008 MHz
NUC2                  1H
CPDPRG[2           waltz16
```

Khaled Omar_H_SL11 BI 11

¹HNMR

Microanalytical Unit - FOPCU - NMR laboratory
www.pharma.cu.edu.eg dir-mau.fopcu@pharma.cu.edu.eg

BRU

Current Data Parameters
NAME Khaled Omar_H_SL11
EXPNO 10
PROCNO 1

F2 - Acquisition Parameters
Date_ 20200824
Time 22.42
INSTRUM spect
PROBHD 5 mm PABBO BB/
PULPROG zg30
TD 65536
SOLVENT DMSO
NS 32
DS 2
SWH 8012.820 Hz
FIDRES 0.122266 Hz
AQ 4.0894465 sec
RG 129.43
DW 62.400 usec
DE 6.50 usec
TE 298.0 K
D1 1.00000000 sec
TD0 1

======== CHANNEL f1 ========
SFO1 400.1924713 MHz
NUC1 1H
P1 15.00 usec
PLW1 10.39999962 W

F2 - Processing parameters
SI 65536
SF 400.1900000 MHz
WDW EM
SSB 0
LB 0.30 Hz
GB 0
PC 1.00

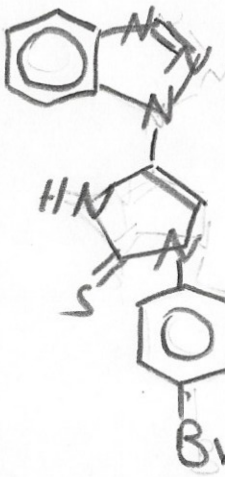

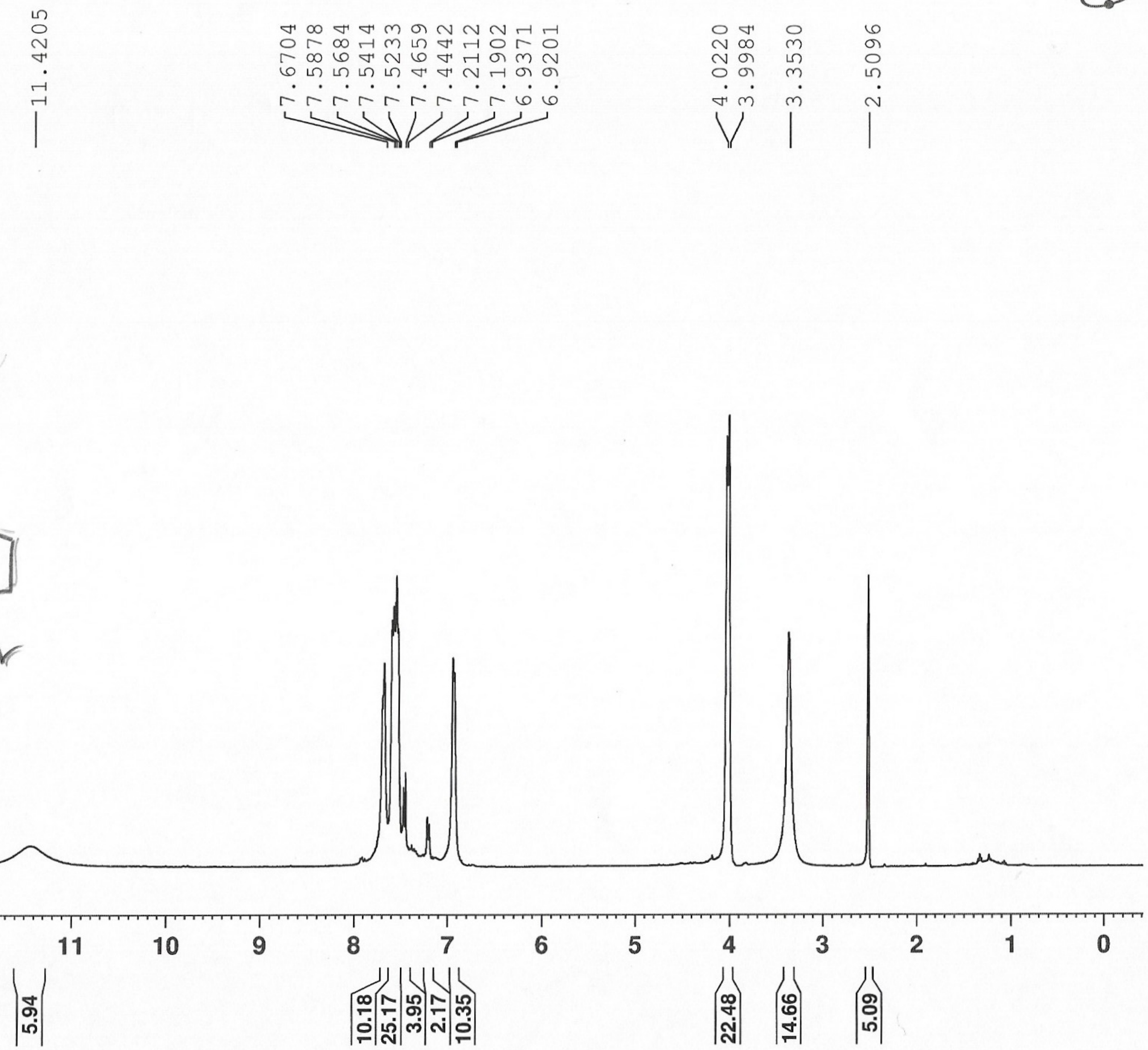

Khaled Omar_H_SL11_D2O

Microanalytical Unit - FOPCU - NMR laboratory
www.pharma.cu.edu.eg dir-mau.fopcu@pharma.cu.edu.eg

BRUKER

BI 11

^{1}HNMR + D2O

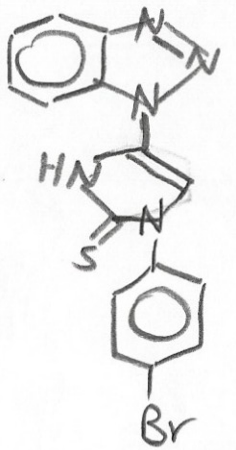

```
Current Data Parameters
NAME       Khaled Omar_H_SL11_D2O
EXPNO                  10
PROCNO                  1

F2 - Acquisition Parameters
Date_            20200825
Time                12.53
INSTRUM             spect
PROBHD    5 mm PABBO BB/
PULPROG              zg30
TD                  65536
SOLVENT              DMSO
NS                     32
DS                      2
SWH              8012.820 Hz
FIDRES           0.122266 Hz
AQ              4.0894465 sec
RG                 129.43
DW                 62.400 usec
DE                   6.50 usec
TE                  298.0 K
D1             1.00000000 sec
TD0                     1

======== CHANNEL f1 ========
SFO1          400.1924713 MHz
NUC1                   1H
P1                  15.00 usec
PLW1          10.39999962 W

F2 - Processing parameters
SI                  65536
SF            400.1900000 MHz
WDW                    EM
SSB       0
LB                   0.30 Hz
GB        0
PC                   1.00
```

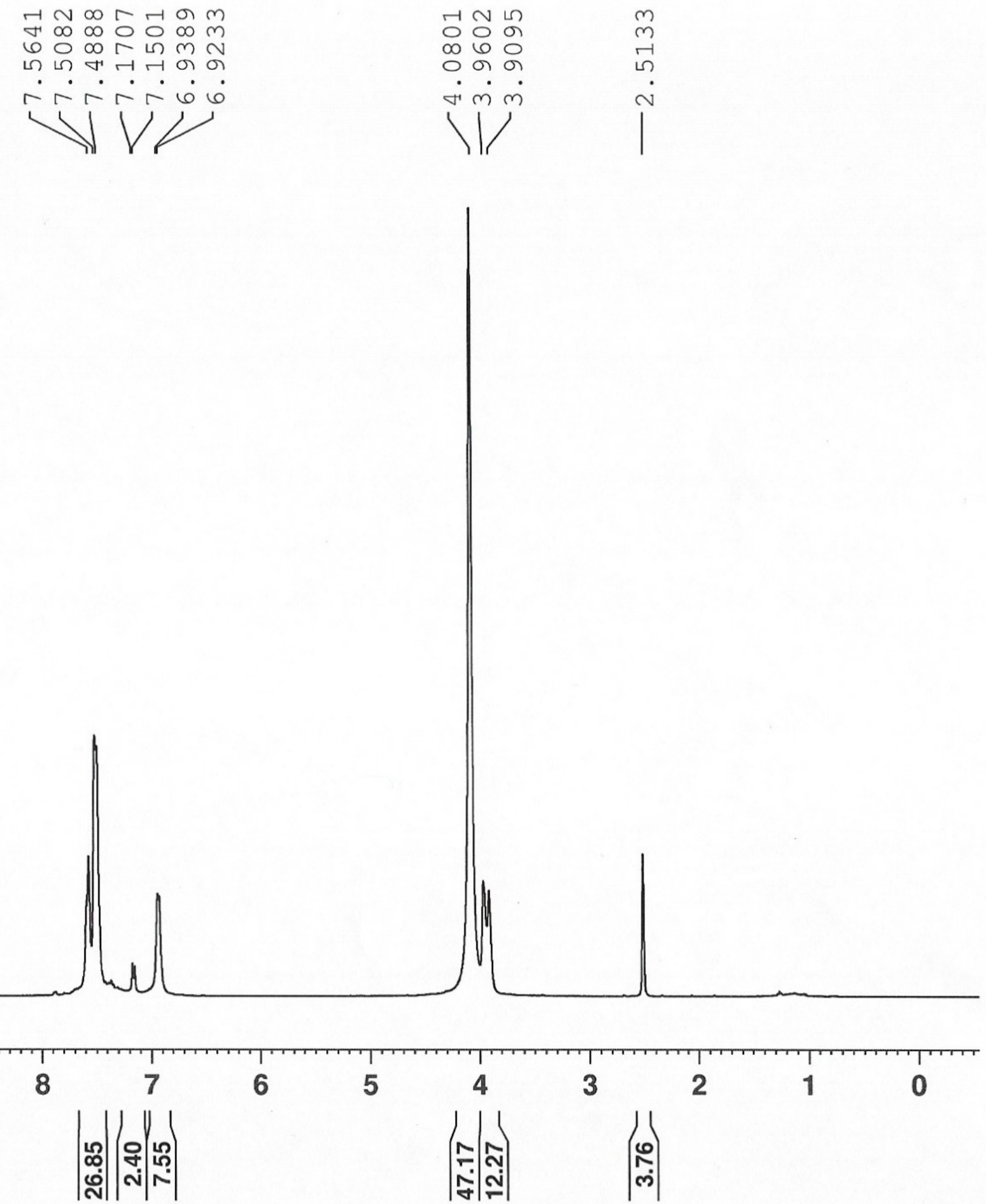

Khaled Omar_C_SL11

BI 11

Microanalytical Unit - FOPCU - NMR laboratory
www.pharma.cu.edu.eg dir-mau.fopcu@pharma.cu.edu.eg

BRU

^{13}C NMR

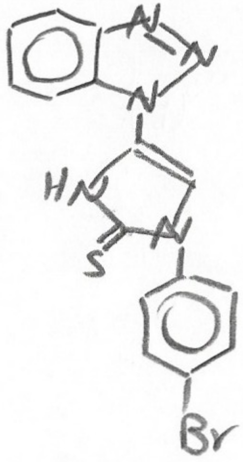


```
Current Data Parameters
NAME      Khaled Omar_C_SL11
EXPNO                   10
PROCNO                   1

F2 - Acquisition Parameters
Date_             20200824
Time                 23.51
INSTRUM              spect
PROBHD   5 mm PABBO BB/
PULPROG            zgpg30
TD                  65536
SOLVENT              DMSO
NS                   1200
DS                      4
SWH            24038.461 Hz
FIDRES          0.366798 Hz
AQ             1.3631488 sec
RG                 202.37
DW                 20.800 usec
DE                   6.50 usec
TE                  298.0 K
D1            2.00000000 sec
D11           0.03000000 sec
TD0                     1

======== CHANNEL f1 ========
SFO1         100.6379178 MHz
NUC1                 13C
P1                 10.00 usec
PLW1         45.00000000 W

======== CHANNEL f2 ========
SFO2         400.1916008 MHz
NUC2                  1H
CPDPRG[2         waltz16
```

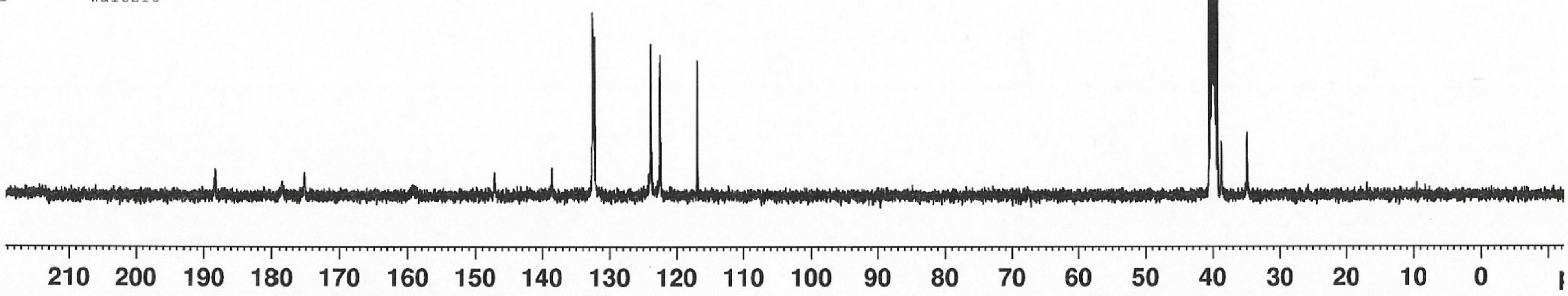

Khaled Omar_H_SL3

BI 12
^{1}HNMR

Microanalytical Unit - FOPCU - NMR laboratory
www.pharma.cu.edu.eg dir-mau.fopcu@pharma.cu.edu.eg

```
Current Data Parameters
NAME      Khaled Omar_H_SL3
EXPNO                    10
PROCNO                    1

F2 - Acquisition Parameters
Date_              20200817
Time                  19.36
INSTRUM               spect
PROBHD   5 mm PABBO BB/
PULPROG                zg30
TD                    65536
SOLVENT                DMSO
NS                       32
DS                        2
SWH                8012.820 Hz
FIDRES             0.122266 Hz
AQ                4.0894465 sec
RG                   146.06
DW                   62.400 usec
DE                     6.50 usec
TE                    298.0 K
D1              1.00000000 sec
TD0                       1

======== CHANNEL f1 ========
SFO1          400.1924713 MHz
NUC1                   1H
P1                  15.00 usec
PLW1          10.39999962 W

F2 - Processing parameters
SI                    65536
SF            400.1900000 MHz
WDW                      EM
SSB      0
LB                     0.30 Hz
GB       0
PC                     1.00
```

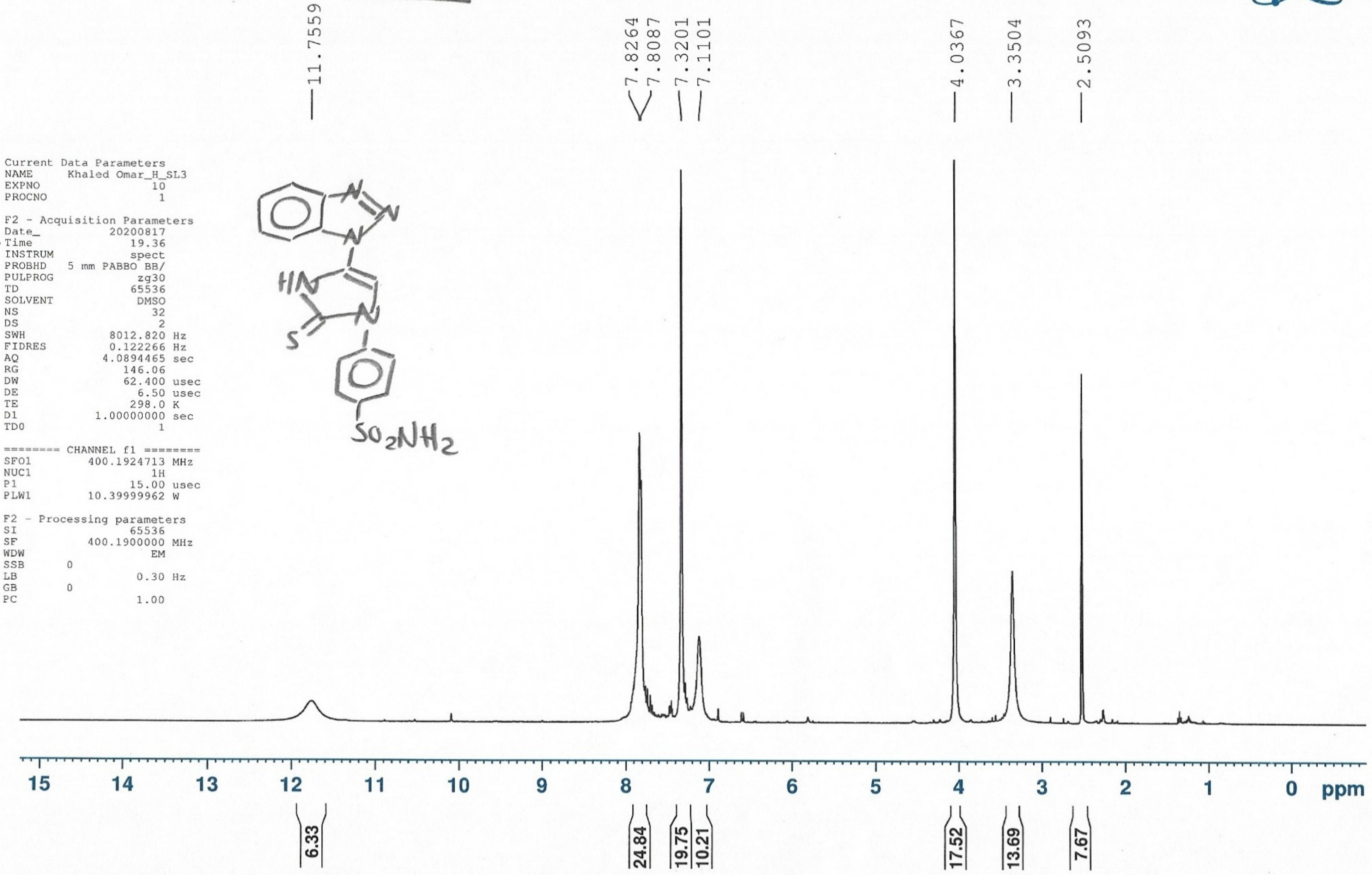

Khaled Omar_H_SL3_D2O

Microanalytical Unit - FOPCU - NMR laboratory
www.pharma.cu.edu.eg dir-mau.fopcu@pharma.cu.edu.eg

BRUKER

BI 12

1HNMR + D2O

```
Current Data Parameters
NAME      Khaled Omar_H_SL3_D2O
EXPNO                   10
PROCNO                   1

F2 - Acquisition Parameters
Date_             20200818
Time                 11.12
INSTRUM              spect
PROBHD    5 mm PABBO BB/
PULPROG               zg30
TD                   65536
SOLVENT               DMSO
NS                      32
DS                       2
SWH               8012.820 Hz
FIDRES            0.122266 Hz
AQ               4.0894465 sec
RG                  146.06
DW                  62.400 usec
DE                    6.50 usec
TE                   298.0 K
D1              1.00000000 sec
TD0                      1

======== CHANNEL f1 ========
SFO1          400.1924713 MHz
NUC1                   1H
P1                   15.00 usec
PLW1           10.39999962 W

F2 - Processing parameters
SI                   65536
SF             400.1900000 MHz
WDW                     EM
SSB      0
LB                    0.30 Hz
GB       0
PC                    1.00
```

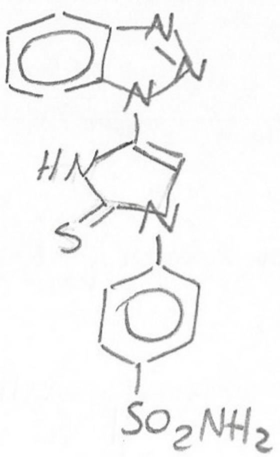

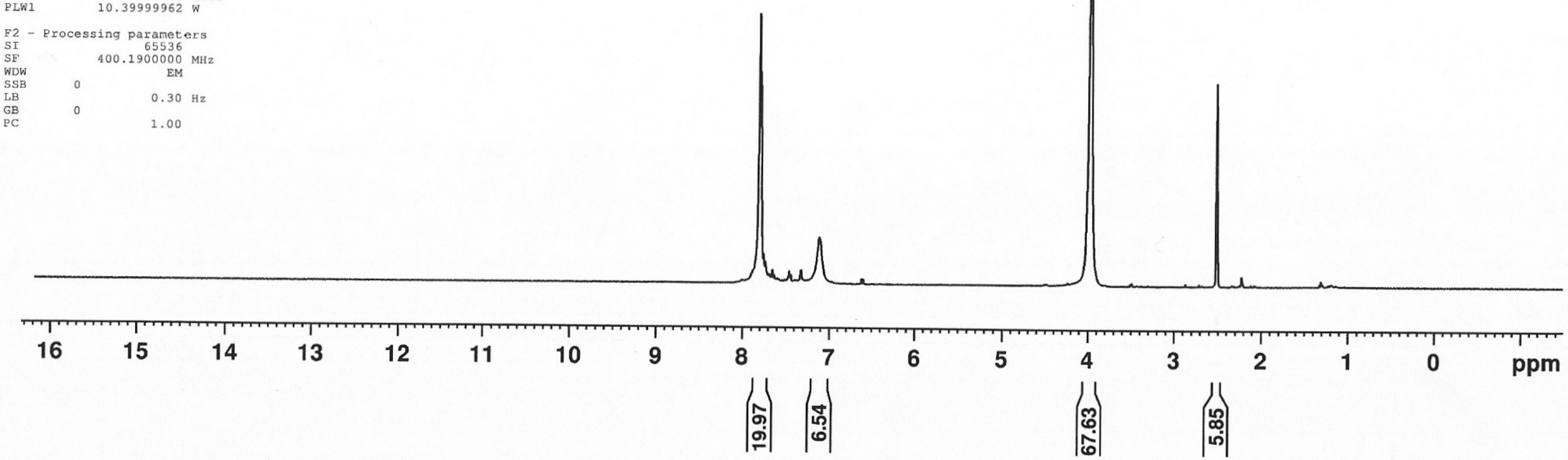

Khaled Omar_C_SL3

B12

^{13}C NMR

Microanalytical Unit - FOPCU - NMR laboratory
www.pharma.cu.edu.eg dir-mau.fopcu@pharma.cu.edu.eg

BRUKER

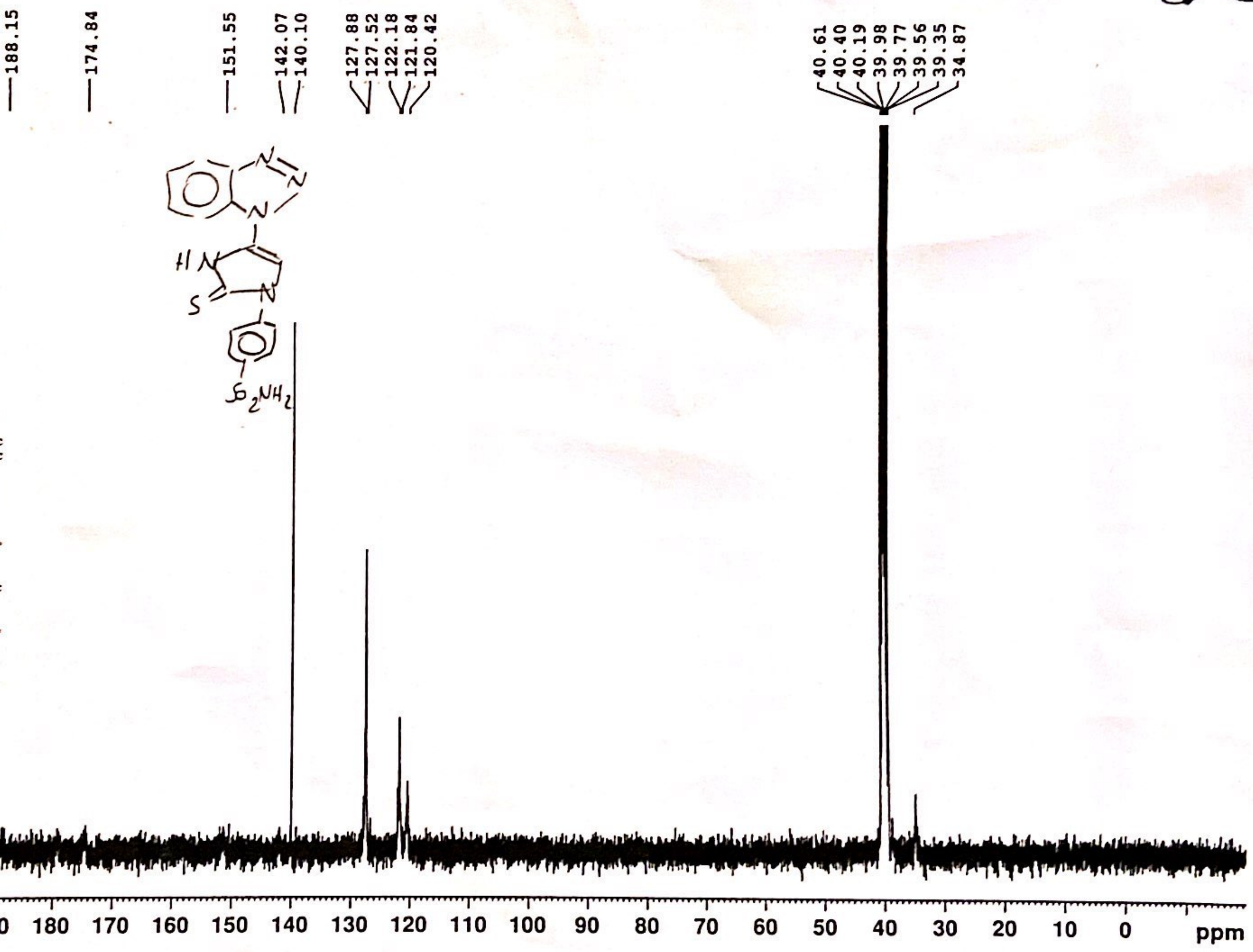


```
Current Data Parameters
NAME          Khaled Omar_C_SL3
EXPNO                    10
PROCNO                    1

F2 - Acquisition Parameters
Date_              20200817
Time                  21.03
INSTRUM               spect
PROBHD     5 mm PABBO BB/
PULPROG              zgpg30
TD                    65536
SOLVENT                DMSO
NS                     1500
DS                        4
SWH               24038.461 Hz
FIDRES             0.366798 Hz
AQ                1.3631488 sec
RG                   202.37
DW                   20.800 usec
DE                     6.50 usec
TE                    298.0 K
D1               2.00000000 sec
D11              0.03000000 sec
TD0                       1

======== CHANNEL f1 ========
SFO1            100.6379178 MHz
NUC1                    13C
P1                    10.00 usec
PLW1            45.00000000 W

======== CHANNEL f2 ========
SFO2            400.1916008 MHz
NUC2                     1H
CPDPRG[2            waltz16
```
